# Integrated proteogenomic characterization of urothelial carcinoma of the bladder

**DOI:** 10.1186/s13045-022-01291-7

**Published:** 2022-06-03

**Authors:** Ning Xu, Zhenmei Yao, Guoguo Shang, Dingwei Ye, Haixing Wang, Hailiang Zhang, Yuanyuan Qu, Fujiang Xu, Yunzhi Wang, Zhaoyu Qin, Jiajun Zhu, Fan Zhang, Jinwen Feng, Sha Tian, Yang Liu, Jianyuan Zhao, Jun Hou, Jianming Guo, Yingyong Hou, Chen Ding

**Affiliations:** 1 State Key Laboratory of Genetic Engineering and Collaborative Innovation Center for Genetics and Development, School of Life Sciences, Institute of Biomedical Sciences, Human Phenome Institute, Fudan University, Shanghai, 200433 China; 2 Department of Pathology, Zhongshan Hospital Fudan University, Shanghai, 200032 China; 3 Department of Urology, Fudan University Shanghai Cancer Center, Shanghai, 200032 China; 4 Department of Oncology, Shanghai Medical College, Fudan University, Shanghai, 200032 China; 5 Institute for Development and Regenerative Cardiovascular Medicine, MOE-Shanghai Key Laboratory of Children’s Environmental Health, Xinhua Hospital, Shanghai Jiao Tong University School of Medicine, Shanghai, 200092 China; 6 Department of Urology, Zhongshan Hospital Fudan University, Shanghai, 200032 China

**Keywords:** Urothelial carcinoma of the bladder, Proteomics, Phosphoproteomics, Genome, RNA-seq, Proteomic subtype, Immune clusters, GARS

## Abstract

**Background:**

Urothelial carcinoma (UC) is the most common pathological type of bladder cancer, a malignant tumor. However, an integrated multi-omics analysis of the Chinese UC patient cohort is lacking.

**Methods:**

We performed an integrated multi-omics analysis, including whole-exome sequencing, RNA-seq, proteomic, and phosphoproteomic analysis of 116 Chinese UC patients, comprising 45 non-muscle-invasive bladder cancer patients (NMIBCs) and 71 muscle-invasive bladder cancer patients (MIBCs).

**Result:**

Proteogenomic integration analysis indicated that *SND1* and *CDK5* amplifications on chromosome 7q were associated with the activation of STAT3, which was relevant to tumor proliferation. Chromosome 5p gain in NMIBC patients was a high-risk factor, through modulating actin cytoskeleton implicating in tumor cells invasion. Phosphoproteomic analysis of tumors and morphologically normal human urothelium produced UC-associated activated kinases, including CDK1 and PRKDC. Proteomic analysis identified three groups, U-I, U-II, and U-III, reflecting distinct clinical prognosis and molecular signatures. Immune subtypes of UC tumors revealed a complex immune landscape and suggested the amplification of *TRAF2* related to the increased expression of PD-L1. Additionally, increased GARS, related to subtype U-II, was validated to promote pentose phosphate pathway by inhibiting activities of PGK1 and PKM2.

**Conclusions:**

This study provides a valuable resource for researchers and clinicians to further identify molecular pathogenesis and therapeutic opportunities in urothelial carcinoma of the bladder.

**Supplementary Information:**

The online version contains supplementary material available at 10.1186/s13045-022-01291-7.

## Background

Bladder cancer is a malignant tumor, which is associated with high morbidity and high mortality rates. Globally, 573,278 new cases and 212,536 related deaths were reported in 2020 [1]. Bladder cancer is more commonly diagnosis at advanced age, with patients' median age at 73 years [2]. The most common pathological type of bladder cancer is urothelial carcinoma (UC), with 75% non-muscle-invasive bladder cancers (NMIBCs) and 25% muscle-invasive bladder cancers (MIBCs) [3]. NMIBCs frequently recur and progress to MIBCs [4], which are usually associated with lower 5-year survival rates, cancer progression, and metastasis [5, 6].

Although NMIBC and MIBC exhibited diverse clinical outcomes, current identified genomic hallmarks of UC, including DDR, MAPK/ERK, and ERBB family genes, were shared by both NMIBCs and MIBCs [7]. The diverse molecular features of MIBCs and NMIBCs have not been clarified. An improved understanding of relationship between NMBICs and MIBCs will be necessary as we evolve toward an objective molecular-based clinical classification.

Elucidation of molecular mechanisms underlying tumor evolution is important for UC biology. On the level of chromosomal alterations in the UC, the loss of 9q appears to occur early in tumor development, whereas the loss of 3p, 10q, 13q, 17p, and 18q is observed more frequently in high-grade tumors. Gains and amplifications are more frequent in patients with advanced tumors. Frequent mutations include *FGFR3, PIK3CA*, *STAG2*, and *RTK/RAS/RAF* pathway genes in NMIBCs and *ERBB2*, *RB1*, *MDM2*, *P53*, *CDKN2A*, *ARID1A*, and *KDM6A* in MIBCs [8]. However, the key drivers of UC tumorigenesis are poorly understood, and the mechanism by which genetic alterations drive cancer phenotypes remains unknown.

Various intrinsic subtypes of UC have been recognized. Researchers from the Lund University (Lund; identified five subtypes: urobasal A, urobasal B, genomically unstable, infiltrated, and SCC-like) [9], the MD Anderson Cancer Center (MDA; divided 73 MIBCs into luminal, p53-like, and basal-like subtypes) [10], the University of North Carolina (UNC; categorized 262 MIBCs into luminal and basal-like subtypes) [11], and the TCGA (clustered 408 MIBCs into five subtypes, luminal-papillary, luminal, luminal-infiltrated, basal–squamous, and neuronal) [12] have confirmed the existence of intrinsic subtypes of UC. Collectively, the molecular subtypes identified independently by different teams exhibited some degree of biological concordance and shared similar clinical characteristics. However, these classifications are mainly based on transcriptional data, whereas classifications based on UC proteome are less studied. With proteins being directly linked to phenotypes, protein-based molecular subtyping holds a promise to provide critical information on translating genome signals to cell function. A comprehensive proteomics profiling of UC is in urgent need.

Therapeutic options for UC include transurethral resection of bladder (TURB), radiotherapy, chemotherapy, targeted therapy, and immunotherapy, or a combination of these treatments [5, 13]. Pembrolizumab and nivolumab were the first two anti-PD-1 monoclonal antibodies to receive FDA approval for bladder cancer [3, 14]. Furthermore, currently reported potential therapeutic targets of UC include transcription factors (TP53, EP300, MDM2), gene integration-related molecules (ERCC2, STAG), RTK signaling pathway (FGFR3), the hedgehog pathway (GLI1, GLI2), etc. [15]. However, targetable mutations remain unknown for a substantial proportion of UCs, and many known drivers have been deemed undruggable [16]. An integrative analysis that harbored both genomics and proteomics can provide insights to nominate potential druggable candidates for therapeutic targets [17, 18].

In this study, comprehensive proteogenomic characterization of treatment-naïve tumors and ﻿morphologically normal human urothelium (MNU) tissues from 116 Chinese UC patients was performed to elucidate the association between genomic variation and phenotypic perturbations. Proteogenomic integration analysis indicated that chromosome 5p gain appears to be a risk factor for progression from NMIBCs to MIBCs. Comprehensive UC proteogenomic analysis exposes proteomic subtypes and immune clusters, which were associated with distinct features in prognosis, genomic alterations, and potential therapeutics. Collectively, our study can serve as an important resource for biological discoveries and therapeutic development of UC in the future.

## Results

### Comprehensive proteogenomic characterization of UC samples

**Fig. 1:**
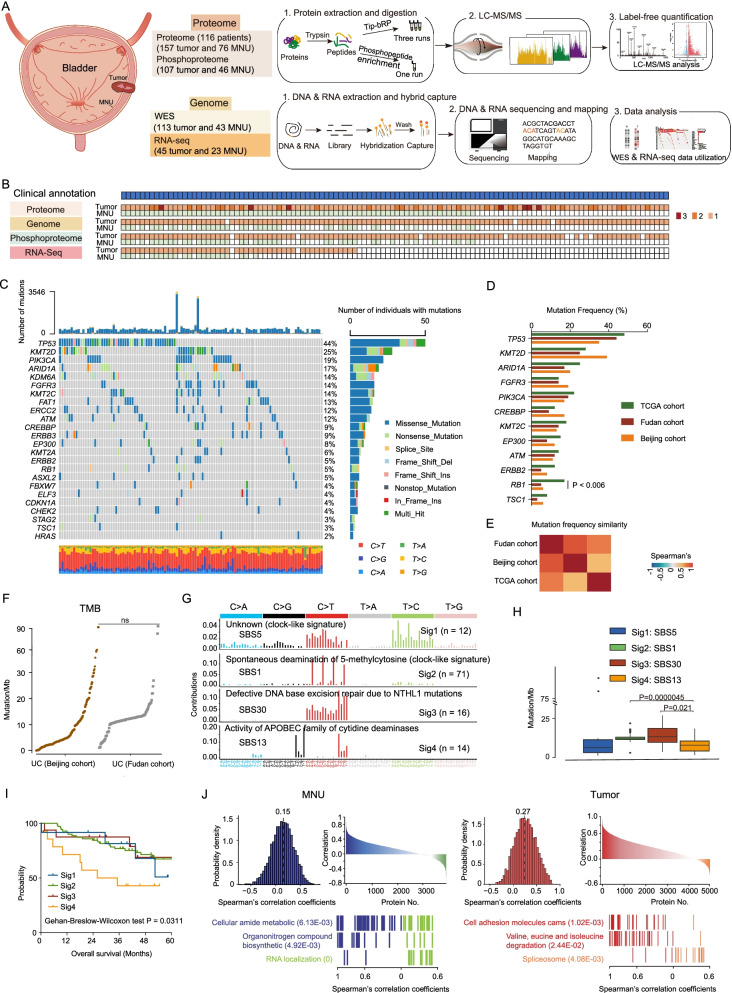
Multi-omics landscape of UC samples. **A** The workflow of the experiment. **B** The number of samples for proteomics, phosphoproteomics, WES, and RNA-seq analysis. **C** The genomic profiles. Top to bottom: synonymous and non-synonymous somatic mutation rates; somatic mutations for significantly mutated genes (SMGs); and potential SMG. Mutation types and their frequencies are depicted by a bar plot in the right panel. **D** Gene mutation frequency in our cohort compared with other cohorts. **E** Correlation plot of the mutation frequencies observed in Fudan cohort compared to TCGA cohort and Beijing cohort. **F** Comparison of TMB in the tumors of our cohort and the Beijing cohort. **G** Mutational spectrum of the four mutational signatures extracted by Sigminer analysis. Corresponding COSMIC signatures are labeled in parentheses. **H** Comparison of TMB in the tumors with different mutational signatures. **I** Kaplan–Meier curves (Gehan–Breslow–Wilcoxon test) for overall survival based on different mutational signatures. **J** Left panel: mRNA–protein correlation in MNUs. Blue: pathways in which positively correlated genes were involved; green: pathways in which negatively correlated genes were involved. Right panel: mRNA–protein correlation in tumors. Red: pathways in which positively correlated genes were involved; orange: pathways in which negatively correlated genes were involved

We collected formalin-fixed paraffin-embedded (FFPE) tumor samples and paired morphologically normal human urothelium (MNU) samples from 116 urothelial carcinoma of bladder (UC) patients, comprising 45 non-muscle-invasive bladder cancers (NMIBCs) and 71 muscle-invasive bladder cancers (MIBCs). The patients were recruited from Zhongshan Hospital (Shanghai, China), and none had a history of presurgical treatment. The samples were re-reviewed by three expert genitourinary pathologists, who classified these as pure urothelial and histological variants. Clinical data, including the gender, age at diagnosis, tumor grade, survival, etc., were summarized in Additional file 13: Table S1. Additionally, comparing to previous published UC dataset, Beijing Cohort [19] and TCGA Cohort [12] revealed the similarity of patients’ basic features including age, gender, history of treatment among the three cohorts, yet some distinctive features were also observed (Table 1). To be more specifical, at demographic level, all the patients in our cohort and Beijing cohort were from Asian, whereas only 7% patients in TCGA cohort were from Asian. Histologically, more early-stage patients (TCGA cohort: Ta-T1, *n* = 1, 0.1%; Beijing cohort: Ta-T1, *n* = 47, 39%; our cohort: Ta-T1, *n* = 45, 38%, chi-square test, *p* < 2.2E−16) were included in our cohort and Beijing cohort (Table 1). A schematic of the experimental design is shown in Fig. 1A. The samples were characterized using clinical data and four molecular profiling platforms (Fig. 1B; Additional file 1: Fig. S1A).

**Table 1:** The baseline characteristics of patients among different cohorts

Characteristic	TCGA cohort (*N* = 412)	Beijing cohort (*N* = 120)	Fudan cohort (*N* = 116)	Chi-square *p* value
Age—no. (%)				
≥ 70 yr	195 (47)	49 (41)	45 (39)	0.173
< 70 yr	217 (53)	71 (59)	71 (61)	
Gender—no. (%)				
Male	108 (26)	31 (26)	28 (24)	0.903
Female	304 (74)	89 (74)	88 (76)	
Smoking—no. (%)				
Yes	288 (70)	30 (25)	18 (15)	< 2.2E−16
No	124 (30)	90 (75)	98 (85)	
Geographical features—no. (%)				
Asian	31 (7)	120 (100)	116 (100)	< 2.2E−16
Others	381 (93)	0	0	
Histologic grade—no. (%)				
High	388 (94)	109 (91)	110 (95)	0.332
Low	21 (5)	11 (9)	6 (5)	
ND	3 (1)	0	0	
T stage—no. (%)				
Ta	0	7 (6)	11 (9)	< 2.2E−16
Tis	0	4 (3)	0	
T1	1 (< 1)	36 (30)	34 (29)	
T2	123 (30)	34 (28)	46 (40)	
T3	196 (48)	36 (30)	22 (19)	
T4	59 (14)	3 (3)	3 (3)	
TX	1 (< 1)	0	0	
ND	32 (8)	0	0	
History of treatment—no. (%)				
Yes	10 (2)	0	0	0.055
No	402 (98)	120 (100)	116 (100)

Using whole-exome sequencing data with a mean depth of 159X in 113 tumors and 128X in 43 paired MNUs (Additional file 1: Fig. S1B), we totally detected 106,622 mutations including 97,950 single-nucleotide variants (SNVs) and 8672 small insertion–deletions (indels) (Additional file 13: Table S1) (Methods). Twenty-four genes showed statistically significant levels of recurrent somatic mutation by analysis using MutSig (*q* < 0.1; Methods) (Additional file 13: Table S1), which included six well-known bladder cancer-related genes: *TP53* 44%, *KMT2D* 25%, *PIK3CA* 19%, *ARID1A* 17%, *RB1* 5%, and *ELF3* 4%. These six SMGs and 20 additional TCGA-reported potential UC driver mutations are shown in Fig. 1C. Correlation analysis was performed using mutational frequencies from other cohorts including Beijing cohort [19] and TCGA cohort [12]. As a result, the mutation frequencies of the hotspot genes detected in our cohort were more closely correlated with Beijing cohort than TCGA cohort (Fig. 1E). Moreover, there was no marked difference between the TMBs in our cohort and the Beijing cohort (Fig. 1F) [19]. This might be due to that both Beijing cohort and our cohort include only Asian patients. Additionally, among the hotspot mutations, the mutational frequency of *RB1* was significantly higher in TCGA cohort than that of in Beijing cohort and our cohort (Fig. 1D; Fisher’s exact test, *p* < 0.05). This finding was in consistent with previous papers that *RB1* displayed higher mutational rates in MIBC [12], since 99% patients in TCGA patients were MIBC, whereas 38% patients in our cohort were NMIBC, respectively.

We identified four mutational signatures by Sigminer (Fig. 1G; Additional file 13: Table S1; Methods). Signatures 1–4 corresponded to the known COSMIC (Catalog of Somatic Mutations in Cancer) signatures: SBS5, SBS1, SBS30, and SBS13. We compared tumor mutation burden (TMBs) of the four mutational signatures; the results showed that SBS30, which represents DNA-based excision repair, had the highest level of TMB (Fig. 1H). In addition, SBS13, representing the activity of APOBEC, was associated with the worst prognosis (Fig. 1I). For comparison, the same enrichment analysis was performed in the TCGA cohort [12], in which four mutational profiles (SBS5, SBS2, SBS13, and SBS10b) were identified (Additional file 1: Fig. S1C). The mutational signatures best matching to those in TCGA cohort were (1) SBS5; unknown (clocklike signature), and (2) SBS13; APOBEC cytidine deaminase (Additional file 1: Fig. S1D). In addition, SBS5 and SBS13 were also identified in Beijing cohort [19].

Label-free quantification measurement of all patient samples (157 tumors and paired 76 MNUs) resulted in a total of 16,440 protein groups with a 1% false discovery rate (FDR) at the protein and peptide levels (Additional file 1: Fig. S1E–G; Additional file 13: Table S1; Methods) [20, 21], and an average of 6990 protein groups per tumor sample and 5945 protein groups per MNU sample (Additional file 1: Fig. S1H). The tumor proteome and the MNU proteome exhibited a unimodal distribution, and the correlations among 157 tumor samples ranged between 0.52 and 0.87 (Additional file 1: Fig. S1I, J). Phosphoproteomic analysis was conducted on 111 tumor samples and 46 MNU samples which revealed 5789 phosphoproteins and 33,233 phosphosites in tumors, as opposed to 3246 phosphoproteins and 11,668 phosphosites identified and quantified in MNUs (Additional file 1: Fig. S1K; Additional file 13: Table S1; Methods). The average Spearman’s correlation coefficient, calculated for all quality control runs of HEK293T cell samples, was 0.9, showing that the MS data were of high quality (Additional file 1: Fig. S1L).

Transcriptional sequencing was carried out on forty-three tumors and paired 22 MNU samples; we identified 17,091 genes per tumor sample and 14,738 genes per MNU sample, with fragments per kilobase of transcript per million mapped reads (FPKM) of more than 1 (Additional file 1: Fig. S1M). The number of genes identified as corresponding to the proteome (unique peptide ≥ 2), phosphoproteome, and transcriptome was 2221 for MNC samples and 4344 for tumor samples (Additional file 1: Fig. S1N). In addition, we calculated the correlation between 5001 mRNA–protein pairs for UC tumors and 3983 mRNA–protein pairs for MNU samples (Fig. 1J; Additional file 13: Table S1). The median correlation value of MNU was 0.15, whereas tumors had a higher median value of 0.26. This result is similar to that of previous studies investigating ccRCC and higher-grade serous ovarian cancers [22, 23]. In MNU samples, 74.6% of mRNA–protein pairs showed positive Spearman correlation coefficients associated with pathways, such as the cellular amide metabolic pathway and organonitrogen compound biosynthetic pathway, whereas genes showing negative correlations were enriched in RNA localization. In tumor samples, 86.7% of mRNA–protein pairs showed positive Spearman correlations with pathways including the cell adhesion molecules cams and valine, leucine and isoleucine degradation, whereas genes with negative correlations were enriched in the spliceosome. The discordance between transcriptomics and proteomics suggests that proteomics data possessed unique oncogenic features that cannot be obtained from genomic and transcriptomic data.

### Effects of copy number alterations

**Fig. 2:**
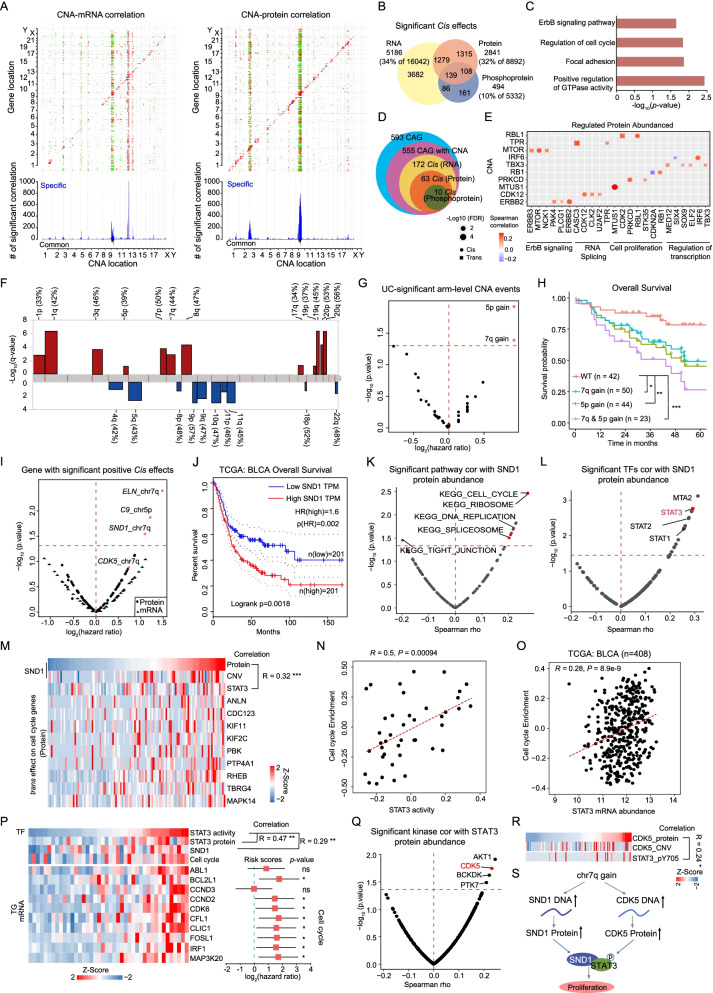
Effects of copy number alterations on mRNA and protein abundance. **A** Functional effects of CNAs on mRNA and proteins. Top panels: correlation of CNA to mRNA and protein abundance. Positive and negative correlations are indicated in red and blue, respectively. Genes were ordered by chromosomal location on the *x* and *y* axes. Diagonal lines indicate cis-effects of CNA on mRNA or proteins. Bottom panels: number of mRNAs or proteins that were significantly associated with a specific CNA. Gray bars indicate correlations specific to mRNA or proteins, and black bars indicate correlations with both mRNA and proteins. **B** Venn diagrams depicting the cascading effects of CNAs. It shows the overlap between significant cis events across the transcriptome, proteome, and phosphoproteome. **C** Pathways enriched for 139 significant cis-effect genes. **D** Venn diagram shows the significant cis events restricted to cancer-associated genes (CAGs) across multiple data types. **E** *Cis*- and *trans*-effects of 10 significant cis-effect CAGs. Affected proteins are grouped by pathway. **F** Arm-level CNAs. Red denotes amplification and blue denotes deletion. **G** Chromosomal alterations associated with prognosis (overall survival). Volcano plot showing log2-based hazard ratio for each alterative chromosome. **H** Overall survival analysis of patients with 5p or 7q gain versus WT (*p* value from log-rank test). **I** Volcano plot showing log2-based hazard ratio (overall survival) for significant positive cis-effect genes on chromosomes 5p and 7q, respectively. The dots represent proteins, and the triangles represent mRNA. **J** Overall survival analyses of BLCA TCGA patients with high or low levels of SND1 mRNA abundance (*p* value from log-rank test). **K** Volcano plot showing the correlation between enriched KEGG pathways scores (sample-specific gene set enrichment analysis (ssGSEA)) and SND1 protein abundance. **L** Volcano plot showing the correlation of transcription factors (TFs) with SND1 based on protein level. TF, highlighted in red, reportedly interacts with SND1. **M** Heatmap of SND1 protein abundance and trans-effect cell-cycle-related proteins. **N** Correlation of STAT3 activity with the cell cycle enrichment score by ssGSEA. **O** Correlation of STAT3 with the cell cycle enrichment score by ssGSEA in TCGA cohort. **P** Heatmap of STAT3 activity change and the target genes of STAT3 that participated in cell cycle. Confidence intervals (95%) of hazard ratio coefficients (overall survival) for each gene mRNA expression level were based on multivariate Cox regression models (tumor samples, *n* = 42). **Q** Volcano plot showing the correlation of kinase with STAT3 based on protein level. The kinase highlighted in red has been reported to be a STAT3 kinase. **R** Correlation and heatmap of CDK5 protein abundance with STAT3 phosphorylation change. **S** A model depicting the gain of chromosome 7q. The *p* values in **K**–**R** were calculated by Spearman's correlation test

We profiled 113 tumors for somatic copy number alteration (CNA) using whole-genome sequencing and examined the regulatory effects of 25,961 CNAs on mRNA, protein, and phosphoprotein expression. CNAs affect mRNA, protein, and phosphoprotein abundance in either “*cis*” or “*trans*” modes, corresponding to the diagonal and vertical patterns (Fig. 2A; Additional file 2: Fig. S2A). Interestingly, cis-regulatory effects of CNA (Fig. 2A; diagonal lines) on mRNAs and proteins were more prominent than those of phosphoproteins (Additional file 2: Fig. S2A). A total of 5186, 2841, and 494 significant correlations (cis-effects) were observed for mRNA, proteins, and phosphoproteins, respectively, with only 139 significant *cis*-effects overlapping among all three omics levels (Fig. 2B). These 139 overlapping genes were significantly enriched in positive regulation of GTPase activity, regulation of cell cycle, focal adhesion, and the ErbB signaling pathway (Fig. 2C), suggesting that core pathways were affected by genomic aberrations. Apart from the overlapped *cis* genes, the *cis*-effects on mRNA, protein, and phosphoprotein were enriched in different pathways. Specifically, mRNA-specific cis-effects (*n* = 3682) were enriched in RNA processing, whereas protein-specific cis-effects (*n* = 1315) were enriched in metabolic pathways, and phosphoprotein-specific *cis*-effects (*n* = 161) were enriched in certain signaling pathways (Additional file 2: Fig. S2B). To further nominate functionally important genes within CNA regions, we focus on the 593 cancer-associated genes (CAGs), in which 555 were identified in our cohort (Fig. 2D; Additional file 14: Table S2). A total of 10 significant positive correlations (*RBL1, TPR, MTOR, IRF6, TBX3, RB1, PRKCD, MTUS1, CDK12,* and *ERBB2*) were observed on mRNA, proteins, and phosphoprotein levels. Moreover, besides *cis-*effects, these ten genes also impacted the expression of proteins enriched in RNA splicing, cell proliferation through *trans*-effects (Fig. 2E). We further investigated the *cis*- and *trans*-effects of these ten CAGs in TCGA cohort [12]. As a result, in consistent with our findings, besides elevating their cognate proteins, these ten CAGs also impacted the expression of proteins participated in cell proliferation, RNA splicing, and transcription pathways through *trans*-effects (Fig. S2C).

CNA analysis showed the most frequent gains in chromosomes 1p, 1q, 3q, 5p, 7p, 7q, 8q, 17q, 19p, 19q, 20p, and 20q and losses in chromosomes 4q, 5q, 8p, 9p, 9q, 10q, 11p 11q, 18p, and 22q (Fig. 2F; Additional file 14: Table S2), and this result was consistent with those of previous studies [24–26]. To be more specific, ten out of twelve most frequent gains detected in our cohort (chromosome 3p, 5p, 8q, and 17q amplifications, etc.) were also identified in TCGA [12] and Beijing cohort [19] (Additional file 2: Fig. S2D, E). Meanwhile, seven out of ten most frequent loss (chromosome 8p and 9p deletions, etc.) detected in our cohort were also identified in TCGA and Beijing cohort (Additional file 2: Fig. S2D, E). In addition, we identified amplifications in driver oncogenes, including *MYCL* (1p34.3, 9 cases), *PPARG* (3p35.3, 13 cases), *ERBB2* (17q12, 10 cases), and *CCNE1* (19q12, 11 cases) (Additional file 2: Fig. S2F; Additional file 14: Table S2), and deletions in key tumor suppressors, such as *CDKN2A* (9p21.3, 26 cases), *RB1* (13q14.2, 13 cases), and *NCOR1* (17p12, 14 cases) (Additional file 2: Fig. S2F; Additional file 14: Table S2). We found that 5p gain and 7q gain were associated with both poor overall survival (OS) and inferior progressive-free survival (PFS) (Fig. 2G, H; Additional file 3: Fig. S3A). On chromosome 5p, 27 and 30 significant positive *cis*-effects were observed on mRNA and protein levels, respectively (Additional file 3: Fig. S3B; Additional file 14: Table S2). *RICTOR*, a *cis*-effect gene assigned to 5p at the protein level, is a critical regulator of cell migration and invasion in bladder cancer cell lines [27]. In chromosome 7q, which is associated with chromosomal instability and many types of neoplasia [28], 19 and 58 significant positive *cis*-effects were observed at mRNA and protein levels, respectively (Additional file 3: Fig. S3C; Additional file 14: Table S2). Proteins overrepresented due to 7q gain were significantly enriched in DNA replication and G1/S transition of the cell cycle (Wilcoxon rank-sum test, *p* < 0.05, 7q gain/WT ratio > 2).

The levels of proteins encoded by the genes linked to significantly positive *cis*-effects on chromosome 5p (*C9*) and 7q (*SND1* and *ELN*)*,* associated with poor prognoses, but the mRNA expression levels were not (Fig. 2I). SND1, a transcriptional co-activator overexpressed in tumors (Additional file 3: Fig. S3D; Wilcoxon rank-sum test, FDR < 0.01), is also associated with poor patient prognoses in the TCGA BLCA cohort (Fig. 2J). Overexpression of SND1 has been detected in various cancer types in TCGA (Additional file 3: Fig. S3E). Notably, SND1 expression significantly increased with pathological stage in both TCGA BLCA and our cohort (Additional file 3: Fig. S3F, G). SND1 is reported to interact with transcription factors, such as STATs and E2F1, modulating the expression of genes that promote carcinogenesis [29–31]. We found that the abundance of SND1 protein in urothelial bladder tumors positively correlated with the cell cycle KEGG (Kyoto Encyclopedia of Genes and Genomes) gene set (Fig. 2K; Additional file 3: Fig. S3H; Spearman’s *r* = 0.27, *p* = 3.5 × 10E−3) and the protein abundance of MCM2, a cell proliferation marker (Additional file 3: Fig. S3I; Spearman’s *r* = 0.3, *p* = 1 × 10E−3). STAT3, a transcription factor that interacts with SND1 [32], showed the highest correlation with the abundance of SND1 protein in tumors (Fig. 2L, M; Spearman’s *r* = 0.32, *p* = 7.3 × 10E−4). A significantly positive correlation between SND1 and STAT3 was also confirmed in the TCGA BLCA cohort (Additional file 3: Fig. S3J; Spearman’s *r* = 0.16, *p* = 1.0 × 10E−3). Furthermore, the predicted STAT3 activity, inferred via mRNA expression of its target genes (Methods), positively correlated with the protein abundance of SND1 (Additional file 3: Fig. S3K; Spearman’s *r* = 0.29, *p* = 6.7 × 10E−3), and patients showing higher expression of STAT3 protein in tumors appeared to have worse prognostic outcomes (Additional file 3: Fig. S3L; log-rank test, *p* = 8.9 × 10^−3^). Expression of many genes participating in the cell cycle, which are STAT3 targets, were upregulated along with increasing STAT3 activity (Fig. 2N, P; Spearman’s *r* = 0.50, *p* = 9.4 × 10E−4). We also confirmed the significantly positive correlation between STAT3 and the cell cycle KEGG gene set in the TCGA BLCA cohort (Fig. 2O; Spearman’s *r* = 0.28, *p* = 8.9 × 10E−9). In addition, phosphorylation of STAT3 also significantly correlated with the protein abundance of SND1 (Additional file 3: Fig. S3M; Spearman’s *r* = 0.26, *p* = 8.2 × 10E−3). STAT3 is reported to be phosphorylated by CDK5 [33]. Surprisingly, we found that CDK5 also exerted a cis-effect on chromosome 7 at the protein level (Fig. 2R; Spearman’s *r* = 0.24, *p* = 1.1 × 10E−2). Further analysis indicated that phospho-STAT3 was positively correlated with the protein abundance of CDK5 in tumors (Fig. 2Q, R; Spearman’s *r* = 0.24, *p* = 1.8 × 10E−2). Supporting our findings, STAT3 was also reported to promote cell proliferation in bladder cancer cell lines, WH, UMUC-3, and 253 J [34]. Our data identified two cis-effects on chromosome 7q, SND1 and CDK5. Upregulation of SND1 expression modulated STAT3 activity, while CDK5 further phosphorylated STAT3, which was related to cell proliferation (Fig. 2S).

### Integrated multi-omics analyses of tumors and MNUs

**Fig. 3:**
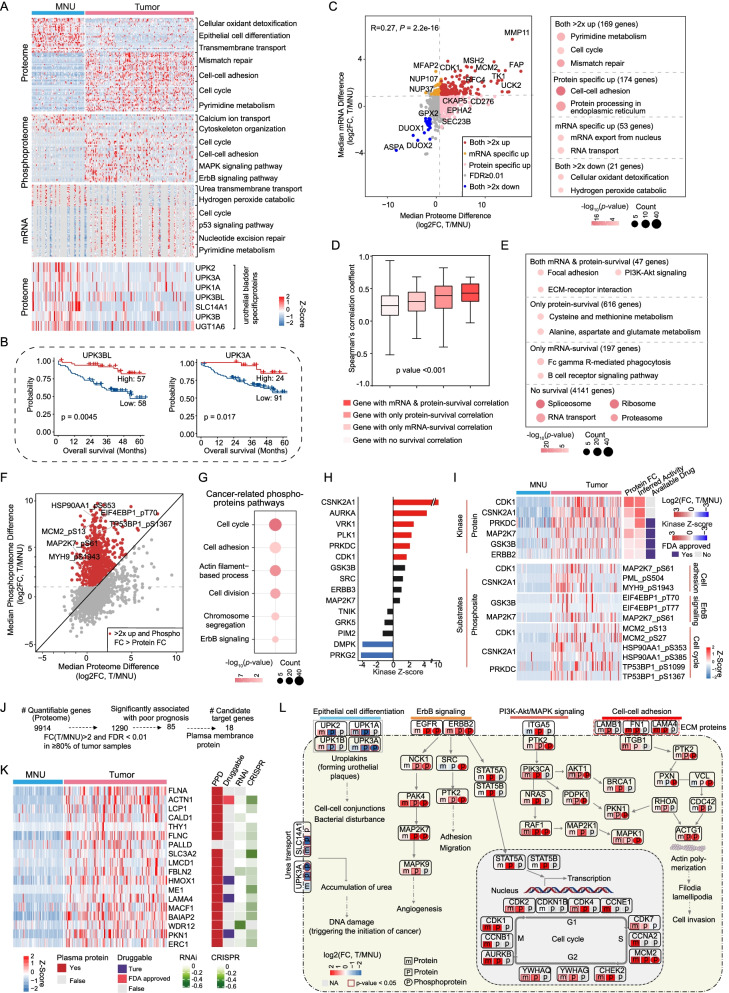
Integrated multi-omics analyses of tumor tissues compared with MNUs. **A** Differentially expressed genes, proteins and phosphoproteins in tumors and MNUs and their associated biological pathways (top panel). A list of urothelial bladder signature proteins that were differentially expressed in tumors and MNUs (*p* value from Wilcoxon rank-sum test) (bottom panel). **B** Two proteins (UPK3BL and UPK3A) were significantly associated with prognosis (overall survival) (p value from log-rank test). **C** Fold changes of genes and proteins in tumors and MNUs (Spearman's *r* = 0.26, *p* = 2.2E−16) (left) and pathways enriched for respective specific changed molecules (right). **D** Boxplot showing the mRNA–protein correlations for the genes associated with significant and nonsignificant differences in patient survival at the protein or mRNA level (*p* value from Kruskal–Wallis test). **E** Pathways enriched for genes with survival differences at protein or mRNA level. **F** Fold changes of proteins and phosphosites, and their correlations in tumors and MNUs. Red dots: phosphosites are greater than twofold changes in tumors compared to MNUs, and changes of phosphosites abundance are greater than changes of their corresponding protein abundance. **G** Pathways enriched with cancer-related phosphosites. **H** KSEA analyses of kinase activities in tumors and MNUs. **I** Heatmap of activated kinases in tumors and substrates corresponding to associated biological pathways (left). Inferred activity was calculated via KSEA analyses, and purple boxes indicate the existence of an FDA-approved drug. (**J** and **K**) Strategy for candidate target genes (**J**) and heatmap showing the proteins that meet the screening criteria (**K**). Cancer dependency map-supported (https://depmap.org) panels on the right show log2-transformed relative survival averaged across all available urinary tract cell lines after depletion of the indicated gene (rows) by RNAi or CRISPR. Their presence in serum was annotated from Plasma Proteome Database (PPD), and drug targets were based on the Drug Gene Interaction Database (http://www.dgidb.org/). **L** Overview of significantly enriched pathways in tumors and MNUs

Multi-omic profiles of both tumors and MNUs were derived, presenting a unique opportunity to explore multi-omic remodeling upon tumorigenesis. We compared differences between tumors and MNUs at different omics levels. Principal component analysis (PCA) of RNA-seq (27,828 genes), proteome data (5546 proteins), and phosphoproteome data (1672 phosphoproteins) showed a clear separation of tumors and MNUs at all three omics levels (Additional file 4: Fig. S4A–C; Additional file 15: Table S3). Differential gene analysis between tumors and MNUs resulted in 1726 mRNA, 2676 proteins, and 784 phosphoproteins (Additional file 4: Fig. S4D–F; Wilcoxon rank-sum test, FDR < 0.01, T/MNU ratio > 2 or < 1/2; Additional file 15: Table S3). For further comparison, we include data from recent publish proteomic landscape of 16 major types of human cancer [35]. By performing comparative analysis, we observed that 225 of the 288 urothelial cancer-type-specific proteins identified by Zhou Y. et al. were also identified in our proteomic data (Additional file 4: Fig. S4G). Further pathway enrichment analysis using these differential genes indicated that upregulation of the cell cycle pathway and downregulation of the transmembrane transport pathway in tumors occurred at all three omics levels (Fig. 3A; Additional file 4: Fig. S4H–J), suggesting that the differences between tumors and MNUs were reflected at all omics levels. Key factors in the cell cycle and transmembrane transport pathways, such as CDK1 and SLC14A1, were correlated with clinical outcomes (Additional file 4: Fig. S4K). Notably, the urothelial bladder-specific proteins nominated by Zhou et al. [35], such as those of the UPK family (uroplakins; UPK3A and UPK3BL among others), were observed to be downregulated in tumors (Fig. 3A; Wilcoxon rank-sum test, FDR < 0.01, T/MNU ratio < 0.5), and patients with higher expression of those uroplakin proteins in tumors appeared to have better prognostic outcomes (Fig. 3B; log-rank test, *p* < 0.05). These proteins are specifically expressed in the urothelial epithelium, necessary for urothelial bladder permeability barrier [36]. The loss of these proteins in tumors implied the cellular atypia due to initiation of urothelial bladder cancer in the urothelial epithelium.

Interestingly, we found divergence in tumors and MNUs differences among the three omics levels. For example, the 174 proteins (Wilcoxon rank-sum test, FDR < 0.01, T/MNU ratio > 2) that showed greater changes in protein abundance than in corresponding mRNA abundance (Wilcoxon rank-sum test, FDR < 0.01, T/MNU ratio < 2) were mainly involved in pathways related to protein processing in endoplasmic reticulum and cell–cell adhesion (Fig. 3C). The correlation between the differences in tumor-MNU protein and mRNA abundance was intermediate compared to previous findings (Fig. 3C, Spearman’s *r* = 0.27; *p* = 2.2 × 10^−16^) [37, 38]. To assess the potential clinical relevance of genes showing significant mRNA–protein correlations, we examined whether these were associated with patient survival. Interestingly, genes associated with significant differences in survival, especially at both protein and mRNA levels, showed the strongest mRNA–protein correlations (Fig. 3D; Additional file 15: Table S3). Further pathway enrichment analysis showed that genes with significant survival differences at both protein and mRNA levels participated in focal adhesion, ECM-receptor interaction, and PI3K-Akt signaling pathways (Fig. 3E), suggesting that alterations in key signaling, especially at all omics levels, were associated with tumorigenesis and tumor progression.

To further investigate the dominant signal transduction pathway, we studied the phosphoproteome. The results revealed that 991 phosphosites mapped to 379 phosphoproteins showing greater changes than corresponding protein abundance (Fig. 3F; Wilcoxon rank-sum test, FDR < 0.01, T/MNU ratio > 2) were significantly enriched in pathways, including the cell cycle and cell–cell adhesion (MCM2-S13, TP53BP1-S1367, and MYH9-S1943 among others) (Fig. 3G). Kinase substrate enrichment analysis (KSEA) of the phosphoproteome of tumors and MNUs revealed the dominant kinases that were activated in tumors, including CSNK2A1, AURKA, VRK1, PRKDC, MAP2K7, and ERBB2 (Fig. 3H; Methods). Among these, many activated kinases, such as PRKDC, MAP2K7, and ERBB2, are targets of approved inhibitors. Further investigation of differentially changed phosphosites showed that elevated substrates involved in the cell cycle, ErbB signaling, and cell–cell adhesion were observed in tumors (Fig. 3I). In addition to kinase targets, plasma membrane proteins are also attractive therapeutic targets in cancer treatment. Therefore, we further performed supervised analysis to filter out plasma membrane proteins (Fig. 3J; Methods). Eighteen proteins (FLNA, PKN1, LAMA4, etc.) met the screening criteria and were annotated for the degree to which short hairpin RNA (shRNA)-or CRISPR-mediated depletion reduced survival and proliferation in urothelial cancer cell lines [39, 40] (Fig. 3K; Additional file 15: Table S3). We further investigated these 18 proteins by assessing immunohistochemistry (IHC) expression in the Human Protein Atlas (HPA). Five of these 18 exhibited overrepresented tumor-specific staining in urothelial bladder cancer samples (Additional file 4: Fig. S4L, M), including PKN1, which had approved drug and were also associated with prognosis (Additional file 4: Fig. S4N). However, the other 13 proteins did not yield staining information or show low staining in HPA, and merit further investigation. An overview of significantly enriched pathways between the tumors and MNUs in urothelial bladder cancer served as guide for future studies and therapeutic opportunities (Fig. 3L).

### Proteogenomic profiles distinguished NMIBC from MIBC

**Fig. 4:**
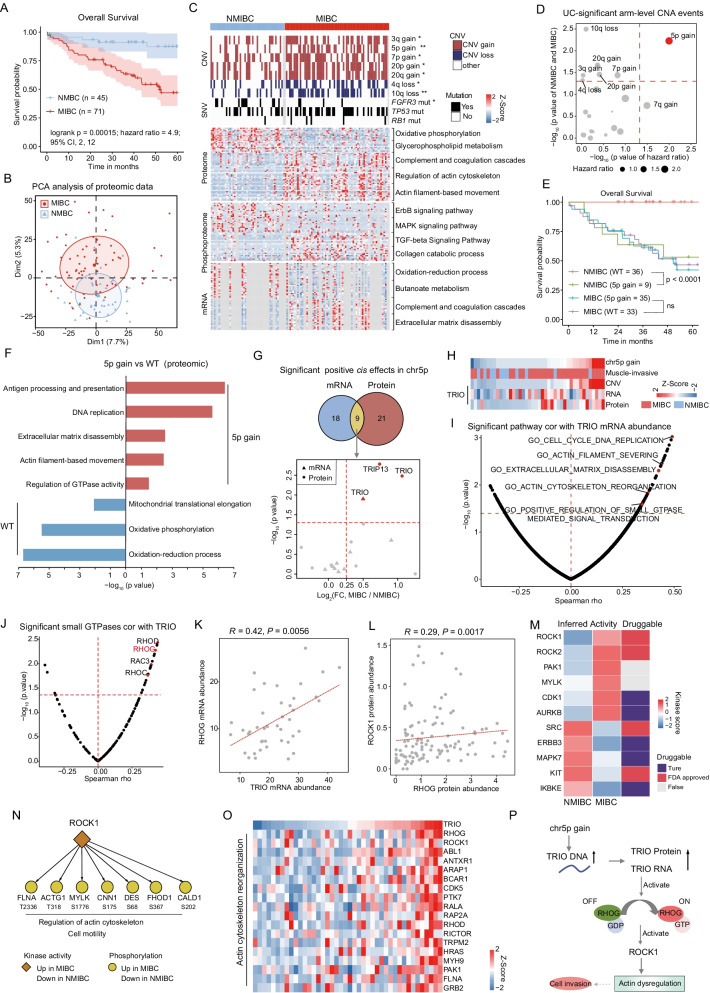
Proteogenomic profiles distinguished NMIBC from MIBC. **A** Overall survival analysis of NMIBC versus MIBC patients (p value from log-rank test). 95% confidence interval was also presented. **B** PCA analysis of proteomic data (5683 proteins) between MIBC and NMIBC. Red dots: MIBC; blue dots: NMIBC. **C** Differentially variational genomic events (top panel) and differentially expressed genes, proteins and phosphoproteins in MIBC and NMIBC and their associated biological pathways (bottom panel). Fisher’s exact test was used for arm-level can events and the status of genes mutation. The Wilcoxon rank-sum test was used for differential expression analysis. **D** Significantly different arm-level CNA events in MIBC and NMIBC and their association with prognosis. **E** Survival analysis of NMIBC and MIBC patients with chromosome 5p gain versus WT (*p* value from log-rank test). **F** Pathways enriched in differentially expressed proteins between 5p gain and WT. **G** Overlap of genes with significant positive cis-effect genes on 5p based on RNA-Seq or proteomic data (top panel) and log2-fold change between NMIBC and MIBC were shown for the nine overlapping genes (bottom panel). The dots represent proteins; the triangles represent mRNA. **H** Heatmap of copy number gain of 5p and the mRNA/protein abundance of TRIO. **I** Volcano plot showing the correlation between enriched Gene Ontology biological processes and TRIO mRNA abundance. **J** Volcano plot showing the correlation between small GTPases and TRIO based on mRNA level. The one highlighted in red is reportedly activated by TRIO. **K** Correlation of TRIO mRNA abundance with RHOG mRNA abundance. **L** Correlation of RHOG protein abundance with ROCK1 protein abundance. **M** Evaluation of kinase activities in tumors of NMIBC and MIBC via KSEA. Drug targets were based on the Drug Gene Interaction Database (http://www.dgidb.org/). **N** Diagram illustrating differences between NMIBC and MIBC tumors in terms of phosphorylation abundance and kinase activity for ROCK1. **O** Heatmap of the mRNA abundance of actin cytoskeleton reorganization related genes. **P** A brief model depicting the functional impact of chromosome 5p gain. The p values in **I**–**L** were calculated by Spearman's correlation test

According to the T-category and histologic grading, UC is divided into NMIBC and MIBC (T-category) or high-grade and low-grade (histologic grading) [41]. Our cohort contained 116 UC patients, comprising 45 NMIBCs (Ta [*n* = 11], T1 [*n* = 34]) and 71 MIBCs (T2 [*n* = 46], T3 [*n* = 22], T4 [*n* = 3]), that were mainly invasive (including propria membrane infiltration and muscle infiltration). As for the histologic grading, MIBCs were all high-grade, while 7 of NMIBCs were low-grade and 38 were high-grade. We showed representative hematoxylin and eosin (H&E)-stained slides of low-grade NMIBC (NMIBC LG)/high-grade NMIBC (NMIBC HG) and MIBC samples (Additional file 5: Fig. S5A–C). MIBC has a poor prognosis compared with NMIBC due to early occult metastatic dissemination [42], which was also observed in our data (Fig. 4A). PCA analysis of RNA-seq (27,752 genes), proteomic (5683 proteins), and phosphoproteomic (2014 phosphoproteins) data separated MIBC samples from NMIBCs (Fig. 4B; Additional file 5: Fig. S5D, E), revealing the molecular differences between MIBCs and NMIBCs. Pathway enrichment analysis of differentially expressed molecules (Additional file 5: Fig. S5F, G; Wilcoxon rank-sum test, *p* < 0.01, MIBC/NMIBC ratio > 2 or < 1/2) showed that NMIBC-enriched molecules were involved in oxidative phosphorylation and lipid metabolism, including glycerophospholipid and arachidonic acid metabolism, whereas molecules enriched in MIBC mainly participated in regulation of actin cytoskeleton and complement and coagulation cascades (Fig. 4C; Additional file 5: Fig. S5H–J). To further investigate the differences among low-grade NMIBC, high-grade NMIBC, and MIBC, we surveyed the differential expressed proteins in the featured pathways in the low-grade NMIBC, high-grade NMIBC, and MIBC. Interestingly, we found that the expression level of proteins participated in MIBCs-enriched pathways gradually increased from low-grade NMIBC, high-grade NMIBC to MIBC, while the expression level of proteins participated in NMIBCs-enriched pathways gradually decreased (Additional file 5: Fig. S5K, L; Kruskal–Wallis test, *p* < 0.0001). For example, proteins that function in regulation of actin cytoskeleton (EHD2, ELN, LCP1, etc.) showed a gradual increasing trend from low-grade NMIBC, high-grade NMIBC to MIBC and presented relative high-risk scores for a mortality prognosis of UC (Additional file 5: Fig. S5M). In contrast, proteins in oxidative phosphorylation and lipid metabolism (NDUFA7, CYP2J2, GPX2, etc.) presented a gradual decreasing trend from low-grade NMIBC, high-grade NMIBC to MIBC and showed low-risk scores for a mortality prognosis of UC (Additional file 5: Fig. S5N). This finding reveals that the differences identified in NMIBC and MIBC were also found in low-grade NMIBC and high-grade NMIBC and presented a gradual tendency from low-grade NMIBC, high-grade MIBC to MIBC.

To determine the divergence of genomic drivers in MIBC and NMIBC, we compared the differences in genomic variations between them. *FGFR3* mutations were observed more frequently in NMIBCs, whereas *TP53* and *RB1* displayed higher mutation rates in MIBCs (Fig. 4C), which was consistent with previous findings [42]. At the arm event level, chromosome gains, such as chromosome 5p, 7p, and 20q gains, were more predominant in MIBCs than in NMIBCs (Fig. 4C; Fisher’s exact test, *p* < 0.05). Among the significant differential arm-level CNA events, only 5p gain was associated with poor prognosis in the entire cohort (Fig. 4D; log-rank test, *p* < 0.05). Surprisingly, we found that the poor prognoses of 5p gains were observed only in NMIBC patients but not in MIBC patients (Fig. 4E; log-rank test, *p* < 1 × 10^−3^). We further investigated the proportion of 5p gains in NMIBC and MIBC patients and found that the percentage of patients with 5p gain in MIBCs (80%) was higher than that in NMIBCs (20%), implying the function of 5p gain in the progression from NMIBC to MIBC. Pathway enrichment analysis using proteomic data showed upregulation of proteins involved in antigen processing and presentation, actin filament-based movement, and regulation of GTPase activity in the 5p gain group, compared with the WT group (Fig. 4F; Additional file 16: Table S4), which was consistent with MIBC-enriched pathways.

To further investigate the potential mechanism, we focused on the *cis*-effects of chromosome 5p. A total of 27 and 30 significantly positive cis-effects were observed at the mRNA and protein levels, respectively, in which nine cis-effects overlapped between both (Fig. 4G). Among these nine cis-effects, only Trio Rho guanine nucleotide exchange factor (TRIO) was significantly upregulated in MIBC as compared to NMIBC at both the mRNA and protein levels (Fig. 4G, H; Wilcoxon rank-sum test, *p* < 0.05, MIBC/NMIBC ratio > 1.5; Additional file 16: Table S4). *TRIO* encodes a large protein that functions as a GDP to GTP exchange factor for Rho GTPases, which plays a role in cell invasion and growth by promoting actin remodeling [43, 44]. The abundance of TRIO at both mRNA and protein levels in urothelial bladder tumors was positively correlated with the reorganization of the actin cytoskeleton gene ontology (GO) biological processes (BP) gene set (Fig. 4I; Additional file 5: Fig. S5O). Expression of RHOG, a TRIO-activating Rho GTPase [45], positively correlated with TRIO at the mRNA level (Fig. 4J, K; Spearman’s *r* = 0.42, *p* = 5.6E−3). A significantly positive correlation between TRIO and RHOG was also observed in the TCGA BLCA cohort (Additional file 5: Fig. S5P; Spearman’s *r* = 0.25, *p* = 3.3E−7). In addition, Rho-associated protein kinases (ROCKs) are reported as the best-characterized downstream effectors of Rho GTPases [46]. The correlation between RHOG and ROCK1 was significantly positive at both mRNA and protein levels (Fig. 4L; Additional file 5: Fig. S5Q). We then performed kinase activity analysis based on the levels of substrate phosphorylation and compared specific activated kinases between MIBC and NMIBC (Methods). As a result, ROCK1 was found to specifically activate kinases in MIBC and was targeted by FDA-approved drugs (Fig. 4M). The expression of ROCK1 substrates (ACTG1 T318, MYLK S1776, CALD1 S202, etc.), which facilitate regulation of the actin cytoskeleton and cell motility, was also upregulated in MIBC (Fig. 4N). Therefore, TRIO activated RHOG and then RHOG activated downstream effectors ROCK1, thereby increasing the reorganization of the actin cytoskeleton (Fig. 4O, P). In sum, our data revealed the potential role of 5p gain in progression from NMIBC to MIBC, through mechanism of modulating actin cytoskeleton implicating in tumor cells invasion.

### Proteomic subtypes of UC and signature proteins

**Fig. 5:**
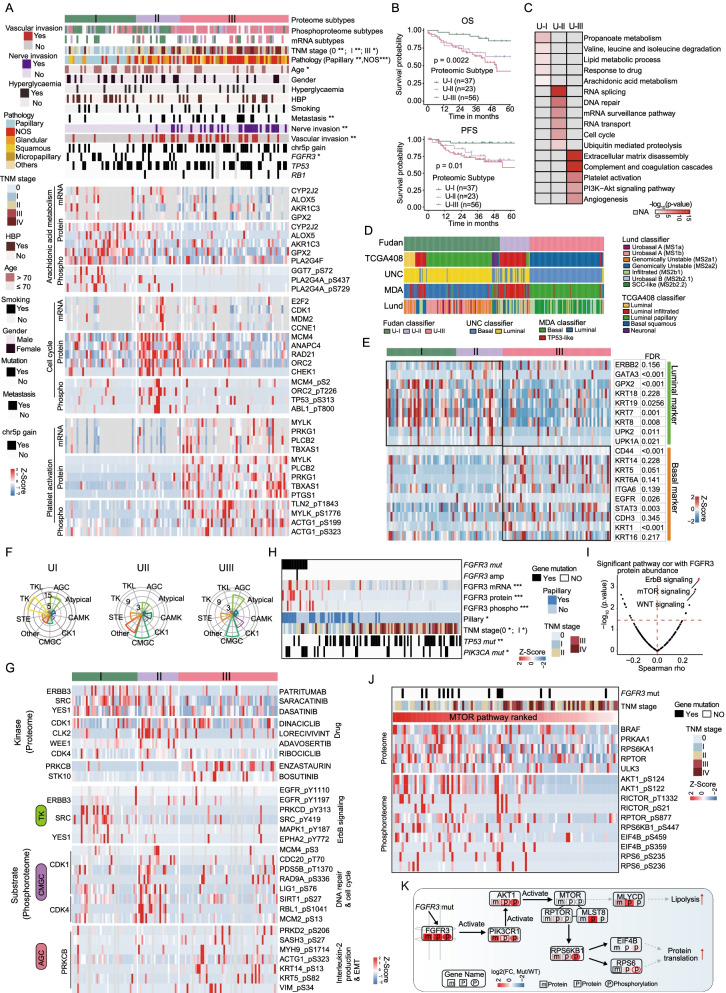
Proteomic subtypes of UC and signature proteins. **A** Heatmap of differentially regulated proteins among the proteomic subtypes (Kruskal–Wallis test, *p* < 0.05), annotated with clinical features. Fisher’s exact test was used for categorical variables: age, gender, hyperglycosemia, HBP, smoking status, metastasis status, status of FGFR3/TP53/RB1 mutation, pathological subtypes, differentiation, and TNM stage. **B** Kaplan–Meier curves for overall survival and progression-free survival of different proteomic subgroups (p value from log-rank test). **C** Pathways significantly enriched in the proteomic subtypes. **D** Comparisons between our classifier and other classifiers. **E** Luminal markers were enriched in the UI and UII subtypes, while basal markers were enriched in the UIII subtype (Wilcoxon rank-sum test, *p* < 0.05). **F** The kinase family was enriched in different proteomic subtypes (Kruskal–Wallis test, *p* < 0.05). **G** Representative kinase and its phosphorylation sites enriched in different proteomic subtypes (Kruskal–Wallis test, *p* < 0.05). **H** The expression of FGFR3 in patients with or without FGFR3 mutation. **I** The pathways correlated with FGFR3 protein abundance. **J** Ranked co-phosphorylation signature of the mTOR pathway aligned with clinical features. **K** Summary of key FGFR3 mutation associated

Consensus clustering identified three proteomic subtypes based on 5489 proteins present in more than 30% of 116 tumors (Fig. 5A; Additional file 6: Fig. S6A; Methods). They were designated U-I (*n* = 37), U-II (*n* = 23), and U-III (*n* = 56) with distinct molecular and clinical features (Fig. 5A–C; Additional file 17: Table S5). Patients in U-I had the best OS and PFS, whereas patients in U-III had the worst OS and PFS (Fig. 5B; log-rank test, *p* < 0.05). Combined with the clinical data, tumors with papillary and NMIBCs were mostly enriched in the U-I (Fig. 5A, Fisher’s exact test, *p* < 0.05), whereas patients in U-III had a higher degree of nerve invasion, metastasis, and vascular invasion (Fig. 5A, Fisher’s exact test, *p* < 0.05). Univariate Cox regression analysis of proteomic subtypes and clinical features is shown in Additional file 6: Fig. S6B. The results revealed that proteomic subtypes (Additional file 6: Fig. S6B; hazard ratio, 2.0; 95% confidence interval [Cl], 1.3–3.1; *p* < 0.001; Additional file 17: Table S5) were authenticated as an independent prognosticator, after controlling for stage, nerve invasion, and vascular invasion.

Subgroup-specific pathway enrichment analysis indicated different features among the three proteomic subgroups. Subgroup U-I was characterized by the highest level of metabolism-related pathways, such as propanoate metabolism, lipid metabolism process, and arachidonic acid metabolism (CYP2J2, ALOX5, AKR1C3, etc.) (Fig. 5C). Subgroup U-II was more related to tumor proliferation, including cell cycle (MCM2, ANAPC4, CHEK1, etc.), RNA splicing, and DNA repair. Subgroup U-III was characterized by pathways, such as those pertaining to extracellular matrix disassembly, complement and coagulation cascades, and PI3K-AKT signaling pathway; some of these were associated with tumor environment and immune response. Genes linked to representative pathways among different proteomic subgroups at different omics levels are shown in Fig. 5A. Notably, some genes were correlated well with the clinical outcomes (Additional file 6: Fig. S6C). Furthermore, multiple previously described UC subtype markers are shown in Additional file 6: Fig. S6D. FGFR3 signatures (IRS1, FGFR3, PTPN13, etc.) were highly expressed in U-I (Wilcoxon rank-sum test, *p* < 0.05), while differentiation signatures (PPARG, SPINK1, DHRS2, etc.) were expressed at higher levels in U-I and U-II. Interestingly, tRNA aminoacylation (GARS, RARS, and TARS) was highest expressed in U-II. EMT signatures (TGFBI, VIM, and CAV1), wild-type p53 signatures (DES, FLNC, CNN1, etc.), and CSC signatures (NES, CD47, and THY1) were overexpressed in U-III. These results emphasized the association between increased biosynthetic, translation, and turnover rates and rapid tumor proliferation.

To directly translate our findings into laboratory tests for tumor classification, we performed differential expression analysis and functional analysis and identified 24 proteins biomarkers that showed dominant expression in a specific proteomic subgroup and were functionally relevant to the main function of the distinctive subgroup (Additional file 6: Fig. S6E). We further performed survival analysis and found eight (CYP2J2, PRKCB, COL1A1, etc.) were correlated with poor prognosis (Additional file 17: Table S5). We then randomly selected three protein marker candidates (CYP2J2, MLH1, and PRKCB) to validate their expression in specific proteome subgroup (U-I, U-II, and U-III). As a result, in consistent with our proteomic data, PRKCB was confirmed to be overrepresented in U-III, MLH1 was overrepresented in U-II, and CYP2J2 was overrepresented in U-I (Additional file 6: Fig. S6F). These suggested that the panel of biomarker candidates could be used to distinguish different subtypes in clinic.

To explore the correlation between proteomic subtypes and mutational signatures, 12 main signatures were identified (Additional file 7: Fig. S7A; Additional file 17: Table S5; Methods). Signature 1 (aging-related) was a dominant identified in 82 patients (Additional file 7: Fig. S7A; Additional file 17: Table S5). The other major signatures were Signature 2 (APOBEC-a, *n* = 33), Signature 6 (defective mismatch repair, *n* = 66), Signature 13 (APOBEC-b, *n* = 23), and Signature 16 (strong transcriptional strand-bias for C>T, *n* = 8). The most dominant signature, Signature 1, was mostly identified in subgroup U-I (Additional file 7: Fig. S7A; Kruskal–Wallis test, *p* < 0.05). Signature 2 (APOBEC-a) and Signature 13 (APOBEC-b) were mainly identified in subgroups U-II and UI-III (Fisher’s exact test, *p* < 0.05). Four signatures were significantly associated with survival, nerve invasion, TNM stage, vascular invasion, and papillary (Additional file 7: Fig. S7B; Wilcoxon rank-sum test, *p* < 0.05). Signature 1 was mostly found in patients carried papillary carcinoma (Additional file 7: Fig. S7B; *p* < 0.05), Signature 6 was mostly observed in patients with lower TNM stage (Additional file 7: Fig. S7B; *p* < 0.05), and Signature 16 was mostly found in patients with vascular invasion (Additional file 7: Fig. S7B; *p* < 0.05).

Furthermore, we conducted clustering analyses on the tumor transcriptome (*n* = 43, consensus clustering) and phosphoproteome (*n* = 105, consensus clustering) (Methods; Additional file 6: Fig. S6A; Additional file 17: Table S5) and also identified three subtypes in each dataset. Moderate concordance among proteomic, transcriptomic, and phosphoproteomic subgroups was uncovered (59.0% between proteome and transcriptome and 39.5% between proteome and phosphoproteome). The phosphoproteomic subgroup with poor overall survival was consistent with the proteomic subgroup U-III. An analysis combining the mRNA, protein, and phosphoprotein helps to decipher the diverse biology and heterogeneity of the molecular processes within UC.

We compared the three clusters with the results obtained from earlier classifiers (Fig. 5D). Comparison between UNC classification [11] and ours indicated that U-I and U-II (as revealed by our classifier) matched well with the luminal subtype, while U-III matched well with the basal-like subtype. In detail, the luminal markers (KRT18, KRT7, GPX2, etc.) were overexpressed in U-I and U-II, whereas basal markers (KRT14, KRT5, STAT3, etc.) were overexpressed in subgroup U-III (Fig. 5E). Comparison between MDA classification and our results indicated that U-I matched well with the luminal subtype, U-II matched well with the TP53-like subtype, while U-III subgroup matched well with the basal subtype (Fig. 5D). Comparison between MDA classification [10] and ours indicated that urobasal A and genomically unstable subtypes were enriched in U-I, the infiltrated subtype was enriched in U-II, and SCC-like and urobasal B subtypes were enriched in U-III (Fig. 5D). Comparison between TCGA classification [8] and ours indicated that U-I agreed well with luminal-papillary and luminal (Fig. 5D), U-II agreed well with luminal-infiltrated, while U-III was consistent with basal–squamous types. These results revealed that our proteome subgrouping showed consistencies with transcriptome-based subgrouping. Since proteins are the major executors of biological functions, the proteomic subgrouping reinforces previous transcriptome data and facilitates the discovery of variant proteins, serving as the resource of the biomarker candidates and therapeutic targets.

Considering that protein kinases have been developed as viable drug targets of cancer therapy, we next inferred kinase activities based on differentially abundant phosphosites in each proteomic subtype, by performing kinase–substrate enrichment analysis (Methods). Significant differences between the inferred activated kinases were observed among the three proteomics subtypes, of which U-I was predominantly featured in the TK kinase group (ERBB3, SRC, YES1, etc.), U-II was characterized by CMGC (CDK1, CDK4, WEE1, etc.), and U-III was characterized by two major kinase groups, AGC (PRKCB, PRKCE, and STK10, etc.) and CMGC (GSK3A, GSK3B, CDK5, etc.) kinase groups (Fig. 5F; Additional file 7: Fig. S7C). Further investigation into the differentially altered phosphosites showed that elevated substrates involved in the ERBB pathway (EGFR-pY1110, SRC-pY149, MAPK1-pY187, etc.) were observed in U-I, DNA repair, and the cell cycle (LIG1-pS76, MCM4-pS3, MCM2-pS13, etc.) in U-II, and Interleukin-2 production and extracellular matrix organization (VIM-pS34, ACTG1-pS323, and MYH9-pS1714, etc.) in U-III (Fig. 5G). These findings suggest that different proteomics subtypes are featured with different kinase and could be treated with corresponding kinase inhibitors. For example, Patritumab, an ERBB3 inhibitor, and Saracatinib, a SRC inhibitor, have the potential to be utilized for patients in U-I, Dinaciclib, a CDK1 inhibitor, and Palbociclib, a CDK4 inhibitor, for patients in U-II, and Enzastaurin, a PRKCB inhibitor, for patients in U-III (Fig. 5G). Importantly, among the potential inhibitors nominated by us, Saracatinib was demonstrated potent antimigratory and anti-invasive effects in vitro and inhibited metastasis in a murine bladder cancer model [47]. CDK4/6 inhibitors (Palbociclib) have been tested in bladder cancer [48] and reported to be potential therapeutic agents for RB positive bladder cancer [49].

Genomic information showed that subgroup U-I and U-II carried a higher mutation rate of *FGFR3* (Fig. 5A; Fisher’s exact test, *p* < 0.05). The mutational hotspots in *FGFR3* in our cohort were similar to those in the TCGA BLCA cohort (Additional file 7: Fig. S7D, E). Notably, most tumors carrying *FGFR3* mutations also harbor *PIK3CA* mutations, while *TP53* and *FGFR3* mutations are mutually exclusive in bladder cancer. To investigate how mutations in *FGFR3* drive its clinical features, we examined the expression of FGFR3 at different omics levels in patients with or without *FGFR3* mutations. The results showed that FGFR3 expression was higher in patients carrying *FGFR3* mutations at mRNA, protein, and phosphoprotein levels (Fig. 5H; Wilcoxon rank-sum, *p* < 0.05; Fold change > 2). To further establish a connection between genetic alterations and corresponding downstream pathways, we explored the correlation between the protein abundance of FGFR3 and enriched pathways. It has been reported that FGFR3 regulates mTORC1/2-cSREBP1 through PI3K/AKT-dependent and PI3K/AKT-independent signaling [50]. Notably, we found that the protein abundance of FGFR3 was positively correlated with the mTOR pathway (Fig. 5I). Additionally, higher expression of FGFR3 was positively correlated with higher phosphorylation of putatively druggable kinases AKT, RICTOR, and RPS6KB1 (Fig. 5J). The summary of the *FGFR3* mutation associations is shown in Fig. 5K.

### Immune cell infiltration in UC tumors

**Fig. 6:**
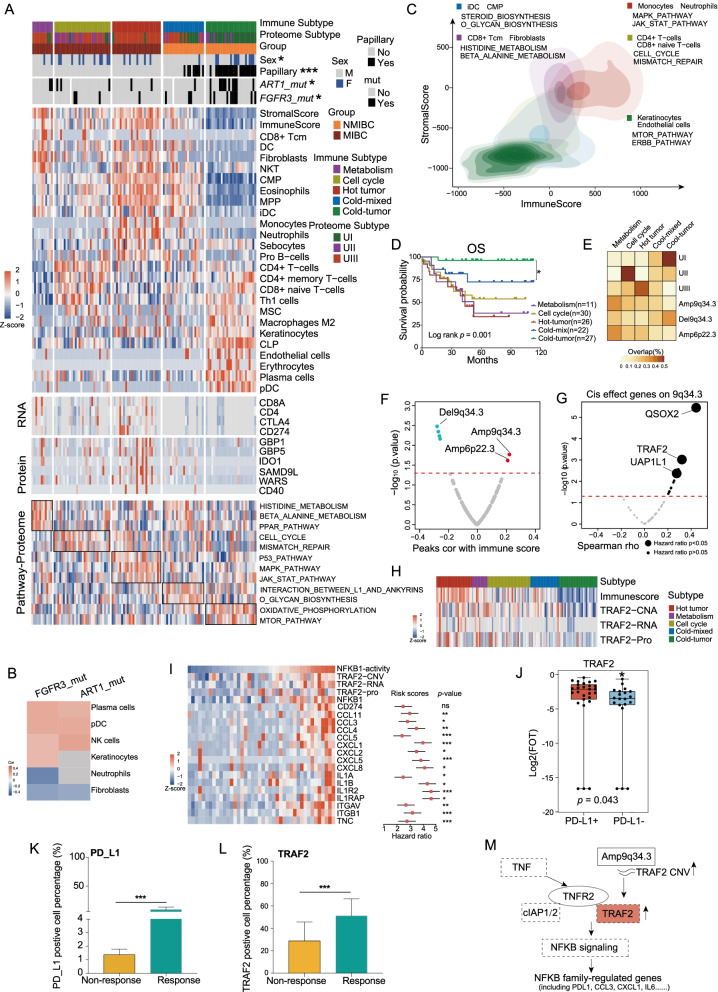
Immune cell infiltration in UC tumors. **A** Heatmap illustrating cell-type compositions and activities of selected individual gene/proteins and pathways across the five immune clusters. The heatmap in the first section illustrates the immune/stromal signatures from xCell. The mRNA and protein abundance of key immune-related markers and ssGSEA scores based on global proteomics data for biological pathways upregulated in different immune groups are illustrated in the remaining sections. **B** xCell immune/stromal signatures in *FGFR3* or *ART1* mutations compared with WT. **C** Contour plot of two-dimensional density based on immunes core (*y*-axis) and stromal scores (*x*-axis) for different immune clusters. For each immune cluster, key upregulated pathways are enriched based on global proteomics (Kruskal–Wallis test, *p* < 0.05). **D** Kaplan–Meier curves for overall survival of different immune clusters (*p* value from log-rank test). **E** Heatmap of the comparison between immune clusters (columns) with proteomic subtypes and different peak events. Each row sums to one, with different blocks showing the proportion of tumors belonging to different immune clusters. **F** Volcano plot showing the correlation between different peak events and immune score. **G** Volcano plot showing the cis-effect genes on 9q34.3 (Spearman’s correlation coefficients, *p* < 0.05). Bigger bubbles showing genes with significant hazard ratio. **H** Heatmap showing the copy number alter, mRNA abundance, protein abundance of TRAF2. **I** Heatmap showing the estimated NFKB1 activity and the mRNA abundance of the targets, the middle red points indicate hazard ratios for each protein, and the endpoints represent lower or upper 95% confidence intervals. **J** TRAF2 was differentially expressed in PD-L1+ group and PD-L1-group. **K**–**L** Boxplots show the quantification of the IHC results. **M** A model depicting the multi-level regulation of TRAF2 copy number alterations

The tumor microenvironment component in our cohort was studied using xCell based on proteomic data of 116 tumors, which had been used in proteomic consensus clustering (Methods; Additional file 18: Table S6). These molecularly based cell-type classifications were supported by ESTIMATE analysis [51] (Methods; Additional file 18: Table S6), yielding the Pearson correlation coefficients of 0.68 and 0.69 for protein and mRNA data of immune- and stromal-derived signatures, respectively (Additional file 8: Fig. S8A). Consensus clustering of the cell signatures identified two NMIBC subtypes and three MIBC subtypes as follows: *Cold-mixed* (*n* = 22); *Cold-tumor* (*n* = 27); *Metabolism* (*n* = 11); *Cell cycle* (*n* = 30); and *Hot-tumor* (*n* = 26) (Fig. 6A, C). Among these, *Cold-mixed* and *Cold-tumor* were obtained from NMIBC. Comparing this with proteomic subtypes, we observed that hot-tumor was enriched in U-III, while the *Cold-tumor* cluster was compatible with U-I (Fig. 6A, E). In addition, combined with clinical data, we observed the immune subgroups significantly differed in OS and PFS (Fig. 6D; Additional file 8: Fig. S8B; log-rank test, *p* < 0.05).

The *Cold-tumor* cluster tumors displayed low immune and stromal scores (Fig. 6A; *t* test, *p* < 0.05), higher frequencies of *FGFR3* mutations, *ART1* mutations, and 9q34.3 deletions (Fisher’s exact test, *p* < 0.05), increased mTOR signaling and ERBB signaling (Wilcoxon rank-sum, *p* < 0.05), as well as the enrichment of endothelial cells and keratinocytes (*t* test, *p* < 0.05). In particular, keratinocytes were upregulated in tumors carrying *FGFR3* mutations compared with the wild-type (Fig. 6B).

The *Hot-tumor* cluster was characterized by the highest immune score and multiple types of immune cells, including neutrophils, eosinophils, and multipotent progenitors (MPP) (Fig. 6A; *t* test, *p* < 0.05). Proteomic analysis has showed that upregulation of immune-related pathways, including that of JAK STAT signaling (Fig. 6A, C; Kruskal–Wallis test, *p* < 0.05), is involved in tumor cell recognition and tumor-driven immune escape [52]. Expression levels of the immune evasion markers, CD40, WARS, SAMD9L, GBP1, and GBP5, were upregulated in this cluster (Fig. 6A; Wilcoxon rank-sum, *p* < 0.05; Fold change > 2), as were the mRNA signatures (CD274 and CTLA4) associated with T cell exhaustion, implicating for immune checkpoint therapy.

The *Cold-mix* cluster was distinguished from the *Cold-tumor* cluster by stronger signatures for Pro B-cells, CMP, and pDC (Fig. 6A; *t* test, *p* < 0.05), upregulation of O-glycan biosynthesis, and interaction between L1 and ankyrins (Fig. 6A; Kruskal–Wallis test, *p* < 0.05), and by containing a higher degree of male patients (Fisher’s exact test, *p* < 0.05). The *Cell cycle* cluster was characterized by CD4+ T cells, CD4+ memory T cells, and Th1 cells (Fig. 6A; *t* test, *p* < 0.05), and regulation of the cell cycle and mismatch repair pathways (Fig. 6A; Kruskal–Wallis test, *p* < 0.05). The *Metabolism* cluster showed a certain degree of similarity with the *Hot-tumor* cluster. This cluster was characterized by fibroblast cells and NKT (Fig. 6A; *t* test, *p* < 0.05), and regulation of metabolism pathways, such as histidine metabolism and beta-alanine metabolism (Fig. 6A; Kruskal–Wallis test, *p* < 0.05). We also found that proliferator-activated receptor (PPAR) signaling pathway (Fig. 6A; Kruskal–Wallis test, *p* < 0.05) was regulated in this cluster.

Interestingly, we found that 9q34.3 amplification, which was significantly positively correlated with the immune score (Fig. 6F; Spearman’s *r* = 0.22, *p* = 0.016), was observed more frequently in the *Hot-tumor* cluster than in the *Cold-tumor* cluster (Fig. 6E; Fisher’s exact test, *p* < 0.05). To further investigate the potential mechanism by which 9q34.3 affects immune activity in UC, we focused on the effect of cis- on 9q34.3. Eleven significantly positive cis-effects were observed at protein level, and three of these (QSOX2, TRAF2, and UAP1L1) were associated with poor prognosis (Fig. 6G; hazard ratio > 2, *p* < 0.05). Tumor necrosis receptor-associated factor 2 (TRAF2) was overexpressed in the *Hot-tumor* cluster tumors, compared to the *Cold-tumor* cluster at both mRNA and protein levels (Fig. 6H; Fold change > 2; Wilcoxon rank-sum test, *p* ≤ 0.061). Next, we found that the protein abundance of TRAF2 was positively correlated with the TNFR2 non-canonical NF-κB pathway (Additional file 8: Fig. S8C; Spearman’s *r* = 0.32, *p* = 5e−4) [53], and the mRNA abundance of TRAF2 was positively correlated with the mRNA abundance of TNF (Additional file 8: Fig. S8D; Spearman’s *r* = 0.37, *p* = 0.016) and TNFR2 (Additional file 8: Fig. S8E; Spearman’s *r* = 0.54, *p* = 3.2e−4). These results indicate the activation of NF-κB1. We further found that the predicted NF-κB1 activities, inferred by the mRNA expression of its target genes (Methods), were positively correlated with the protein abundance of TRAF2 (Additional file 8: Fig. S8F; Spearman’s *r* = 0.32, *p* = 0.039). Many target genes of NF-κB1 were upregulated along with the increase in the NF-κB1 activity (Fig. 6I). Some studies had revealed that several signaling cross talk pathways, such as p53, STAT3, and NF-κB, regulate PD-L1 expression [54]. Surprisingly, the mRNA abundance of PD-L1, a target of NF-κB1, was positively correlated with the mRNA abundance of TRAF2 (Additional file 8: Fig. S8G; Spearman’s *r* = 0.39, *p* = 0.012). The significant correlation between TRAF2 and PD-L1 was also observed in the TCGA BLCA cohort (Additional file 8: Fig. S8H; Spearman’s *r* = 0.32, *p* = 2.2e−11). Furthermore, the mRNA abundance of TRAF2 was positively correlated with CD8 enrichment score (Additional file 8: Fig. S8I; Spearman’s *r* = 0.33, *p* = 0.033), indicating that TRAF2 plays a role in peripheral CD8+ T cell [55].

Based on the observation above, we performed further investigation on PD-L1 immunotherapy in our cohort. To be more specific, by surveying the clinical data of patients in our cohort, we found that 47 patients were detected for PD-L1 expression by IHC, in which 27 patients were defined as PD-L1 positive (TPS: > 1%), whereas 20 patients were defined as PD-L1 negative (TPS: < 1%). Further comparative analysis between PD-L1 positive and negative patients confirmed the positive association between the elevated expression of TRAF2 and increased PD-L1 expression (Fig. 6J; Additional file 8: Fig. S8J).

To further validate the positive association between TRAF2 and PD-L1, we collected FFPE samples from an independent validation cohort containing 14 UC patients treated with PD-L1 inhibitors (5 responders (PR, partial response), 9 non-responders (PD, progressive disease)). Clinical data are summarized in Additional file 18: Table S6. We examined the expression of TRAF2 and PD-L1 on the tissue level by immunohistochemistry of consecutive slides and observed significant elevated expression of both TRAF2 and PD-L1 in responders compared to non-responders (Fig. 6K, L; Additional file 8: Fig. S8K). This result further confirmed that the elevated expression TRAF2 is associated with the increased expression of PD-L1 and is related to patients’ responses. A summary of the TRAF2 amplification associations is shown in Fig. 6M.

### Clinical features associated with proteomic and phosphoproteomic profiles

**Fig. 7:**
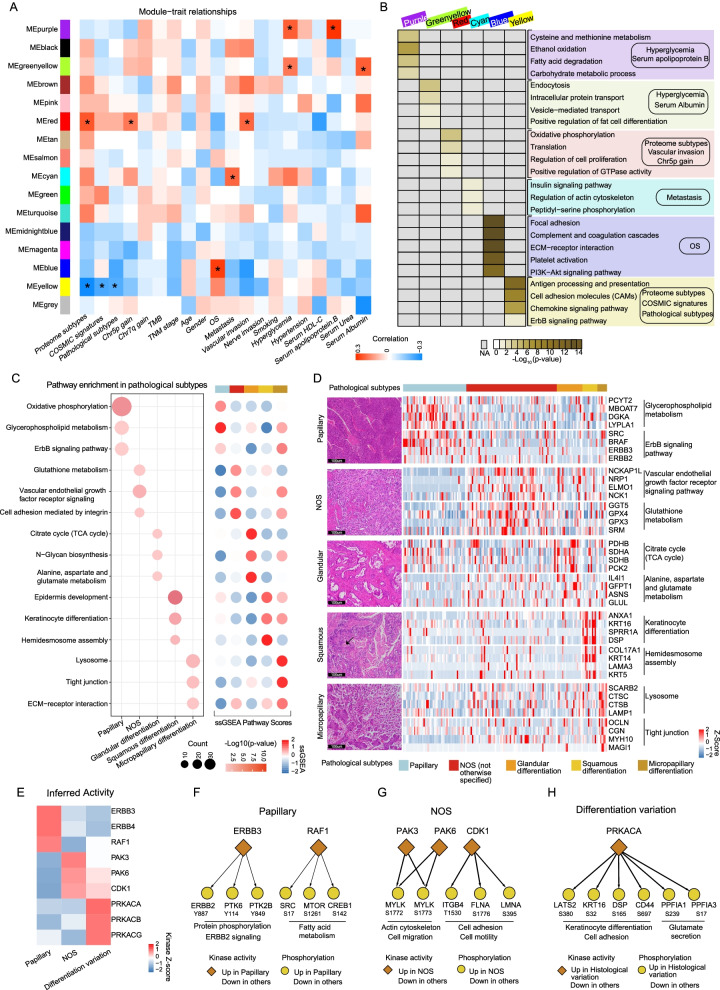
Clinical outcomes associated with proteomics and phosphoproteomic profiles. **A** Heatmap showing the correlation between modules obtained from WGCNA analysis and clinical outcomes. **B** Enrichment pathway of different modules (Wilcoxon rank-sum test, *p* < 0.05). The dot plot on the left summarizes ssGSEA pathway scores based on proteomics data among samples with different histological variation statuses. **C** The ssGSEA pathway analysis of different histological variations (Wilcoxon rank-sum test, *p* < 0.05). **D** Signature proteins of pathways associated with different histological variations (Wilcoxon rank-sum test, *p* < 0.05). **E** Evaluation of kinase activities in tumors across different histological variation via KSEA. **F**–**H** Diagram showing kinase–substrate associations among papillary, NOS, and differentiation variation tumors

To explore the biological characteristics of our cohort in an unbiased proteome-wide manner, weighted correlation network analysis (WGCNA) was performed using 6692 proteins present in more than 10% of the 116 tumors (Methods). The clustering dendrogram of the samples is shown (Additional file 9: Fig. S9A–C; Additional file 13: Table S1). Co-expression analysis yielded 15 consensus modules (Fig. 7A), ranging in size from 157 proteins (MEmidnightblue module) to 2257 proteins (MEturquoise module). The modules were subsequently analyzed by pathway enrichment to characterize the associated biology (Fig. 7B; Additional file 19: Table S7).

Among these modules, MEred was significantly correlated with chromosome 5p gain (Fig. 7A; *r* = 0.18, *p* < 0.05). The genes in this module were enriched in regulation of cell proliferation (Fig. 7B; *p* = 2.21E−04) and positive regulation of GTPase activity (*p* = 2.21E−04). In addition, we found that serum albumin value and serum urea levels were correlated with poor prognosis (Additional file 9: Fig. S9D–E; log-rank test, *p* < 0.05). It has been reported that serum albumin shows potential as a reliable biomarker of inflammation. We further found that serum albumin was significantly correlated with the MEgreenyellow module, where pathway analysis of the genes in this module showed that abnormal serum albumin was associated with endocytosis (Fig. 7B; *p* = 6.89E−04), intracellular protein transport (*p* = 7.5E−03) and vesicle-mediated transport (*p* = 0.015). Furthermore, the MEblue module was significantly correlated with OS, in which 211 out of 607 genes were significantly correlated with clinical outcomes. The genes in this module were enriched in focal adhesion (Fig. 7B; *p* = 2.85E−34), complement and coagulation cascades (*p* = 2.29E−26), and the PI3K-AKT signaling pathway (*p* = 8.09E−11). We further performed supervised analysis to filter potential drug targets (https://www.proteinatlas.org/), and 23 genes (GARS, CFI, MYLK, etc.) met the criteria, in which six (GARS, CAV1, P4HA2, etc.) were reportedly correlated with poor prognosis and overrepresented in the staining of urothelial bladder cancer samples in the HPA database (Additional file 9: Fig. S9F–G; Additional file 19: Table S7).

Heterogeneity of histopathological characteristics adds complexity to the diversity of bladder cancer. Histologically, UC is divided into papillary (papilloma, low malignant potential, and papillary carcinoma) and non-papillary (urothelial carcinoma in situ and invasive) categories [56]. The non-papillary category is further classified into several differentiation forms, such as glandular differentiation, squamous differentiation, micropapillary differentiation, and invasive urothelial carcinoma not otherwise specified (NOS). Papillary carcinoma, NOS, glandular differentiation, squamous differentiation, and micropapillary differentiation accounted for 29.3%, 42.0%, 11.5%, 7.0%, and 3.8% of all tumor samples, respectively (Additional file 9: Fig. S9H). These results are consistent with those of previous studies [57, 58].

To find the divergence of tumors with different histological variants at the molecular level, proteomic data were analyzed using gene set enrichment analysis (GSEA) (Fig. 7C–D; Additional file 19: Table S7). The results revealed that metabolism-related pathways, such as oxidative phosphorylation and glycerophospholipid metabolism (PCYT2, MBOAT7, DGKA, etc.), were enriched in papillary carcinoma. Vascular endothelial growth factor receptor signaling (NRP1, ELMO1, NCK1, etc.), as well as cell adhesion mediated by integrin, was enriched in NOS. Furthermore, different differentiation variants were correlated with different pathways. For example, glandular differentiation was characterized by the citrate cycle (TCA) (PDHB, SDHA, PCK2, etc.) and N-glycan biosynthesis and alanine, as well as by aspartate and glutamate metabolism (IL4I1, GFPT1, ASNS, etc.). Squamous differentiation was characterized by keratinocyte differentiation (ANXA1, KRT16, SPRR1A, etc.) and hemidesmosome assembly (KRT14, LAMA3, KRT5, etc.). Micropapillary differentiation was distinguished by lysosomes (CTSC, SCARB2, LAMP1, etc.) and tight junctions (OCLN, CGN, MYH10, etc.).

To systematically identify druggable targets specific to histological variants, we performed functional enrichment analysis using phosphoproteomic data. The results showed that phosphoproteins showing high expression in papillary carcinoma were enriched in ERBB signaling and MAPK signaling. Phosphoproteins upregulated in NOS were enriched in focal adhesion and muscle contraction (Additional file 9: Fig. S9I). In addition, we pooled the tumors showing different differentiations, such as glandular differentiation, squamous differentiation, and micropapillary differentiation, into one group and named this the differentiation variation. We found that keratinocyte differentiation and cell division were enriched in the differentiation variation group (Additional file 9: Fig. S9I). Various kinase activities in tumors with different histological variations were assessed. ERBB3/ERBB4/RAF1 kinases were activated in papillary carcinoma compared to other variants, PAK3/PAK6/CDK1 kinases were activated in NOS, and PRKACA/PRKACB/PRKACG kinases were activated in differentiation variants (Fig. 7E). These kinases and corresponding substrates are shown in Fig. 7F–H. Among these kinases, CDK1 is reportedly the only essential member of the CDK subfamily, which plays an important role in cell cycle progression [59]. In summary, different histological variants were characterized by different pathways and activated kinases, providing evidence for the need for personalized treatment.

### GARS promotes bladder cancer cell proliferation through non-canonical function

**Fig. 8:**
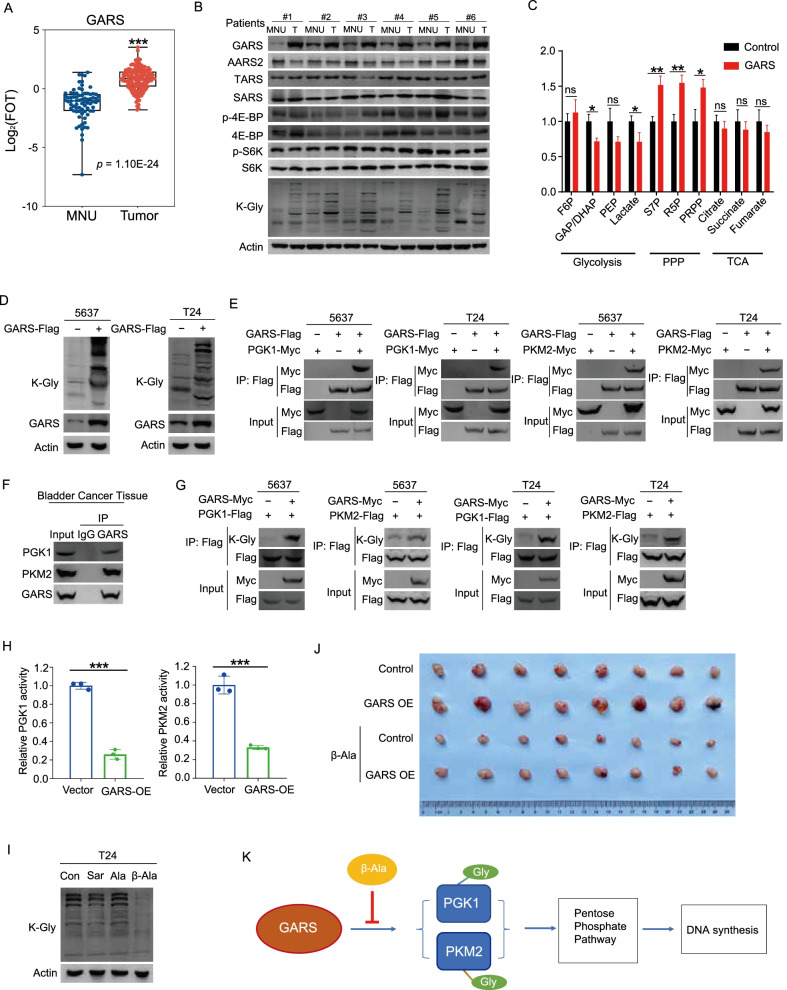
GARS promotes bladder cancer cell proliferation through non-canonical function. **A** GARS was differentially expressed in tumors and MNUs (*p* value from Wilcoxon rank-sum test). **B** The expression levels of indicated proteins and global K-Gly in tumor tissues compared with those of adjacent normal tissues. **C** The pentose phosphate pathway was activated, while glycolysis was downregulated in GARS-overexpressing cells. **D** Global K-Gly levels in T24 and 5637 GARS-overexpressing cell lines. **E** Interaction between GARS and PGK1, and interaction between GARS and PKM2, in both 5637 and T24 cell lines detected by co-immunoprecipitation assays. **F** Interaction of GARS with PGK1 and PKM2 in the bladder cancer tumor tissues, detected by co-immunoprecipitation assays. **G** K-Gly levels of PGK1 and PKM2 in both 5637 and T24 GARS-overexpressing cells. **H** Enzymatic activities of PGK1 and PKM2 in T24 GARS-overexpressing cells. **I** Beta-alanine inhibits K-Gly formation. **J** The effect of beta-alanine and GARS on T24 cells xenografts in nude mice. **K** A model depicting the regulation of GARS

We found that the expression levels of GARS, which is known to be significantly increased in tumor tissues compared to MNUs, were also increased during UC progression (Fig. 8A). Since GARS has not been reported as being associated with the onset of bladder cancer, we explored the role of GARS in bladder cancer progression. Using western blotting, we confirmed that GARS protein levels were profoundly upregulated in tumor tissues (Fig. 8B). Other kinds of aminoacyl-tRNA synthetases, including AARS, TARS, and SARS, which were used as controls, were not significantly altered (Fig. 8B). Overexpressing GARS in the human urinary bladder carcinoma cell lines, T24 and 5637, promoted DNA synthesis (Additional file 10: Fig. S10A–B), cell cycle progression (Additional file 10: Fig. S10C–D), and cell proliferation (Additional file 10: Fig. S10E–F), while knocking down GARS inhibited these three processes. Moreover, although induction of genotoxic stress by cisplatin led to cell cycle progression block and decreased cell proliferation in both T24 and 5637 cells, increase in GARS in cisplatin-treated cells rescued the blocked cell cycle and exhibited a stronger pro-proliferative effect, compared to GARS in normal cell cultures (Additional file 10: Fig. S10A–F). The levels of other types of aminoacyl-tRNA synthetases, as well as the levels of 4EBP and S6K, were not altered between tumors and MNUs in UC, suggesting that the oncogenic role of GARS was not due to protein translation (Fig. 8B). Measuring the metabolites profile using LC–MS revealed that the pentose phosphate pathway was activated, while glycolysis was downregulated in GARS-overexpressing cells (Fig. 8C). These results suggest that upregulated GARS enhances DNA synthesis and promotes cell proliferation by activating pentose phosphate pathway flux. However, the mechanism by which GARS regulates glucose metabolism remains unknown.

### GARS enhances metabolic flux in pentose phosphate pathway by inhibiting PGK1 and PKM2

In addition to mediating protein translation, GARS catalyzes the glycine modification of protein lysine residues (K-Gly) and transfers the glycine signal by altering the function of the modified protein K-Gly [60]. Accordingly, we found that K-Gly levels were significantly increased in tumor tissues, compared to MNUs, in UC patients (Fig. 8B). K-Gly levels were determined by the concentration levels of GARS and glycine. Nuclear magnetic resonance indicated that glycine levels between the tumor and MNUs of UC were not altered (Additional file 11: Fig. S11A), suggesting that increased K-Gly levels seen in UC were caused by upregulated GARS protein abundance. Furthermore, we validated that, in both cultured T24 and 5637 cells, overexpression of GARS led to increased global K-Gly levels in cells (Fig. 8D). In a previous study of our investigating protein lysine aminoacylation [60], we searched for lysine aminoacylation in a tryptic peptide library of human liver cancer and identified large numbers of proteins, as well as potential K-Gly modified proteins. The search results suggested that the enzymes in glycolysis were enriched, including PGK1 and PKM2 (Additional file 20: Table S8). To verify this, we first validated that the interaction between GARS and PGK1, as well as the interaction between GARS and PKM2, using co-immunoprecipitation assays with proteins exogenously expressed proteins in both 5637 and T24 cell lines (Fig. 8E). Moreover, in the tumor tissues, we used a co-immunoprecipitation assay to validate interactions between GARS and PGK1 as well as between GARS and PKM2 (Fig. 8F). Accordingly, we found that overexpression of GARS led to increased K-Gly levels of PGK1 and PKM2, in both 5637 and T24 cells (Fig. 8G). Although the abundances of PGK1 and PKM2 were not altered by GARS (Additional file 11: Fig. S11B), enzymatic activities of PGK1 and PKM2 were decreased in GARS-overexpressing T24 cells (Fig. 8H), suggesting that increased GARS levels enhanced the pentose phosphate pathway flux through attenuating the glycolysis flux by reducing the activities of PGK1 and PKM2. To inhibit the GARS-induced oncogenic effect on cells, we tested whether the structural analog of glycine was able to inhibit the K-Gly formation in cells, including alanine, beta-alanine, and sarcosine (Additional file 11: Fig. S11C). We found that only beta-alanine inhibited K-Gly formation in cultured cells (Fig. 8I). Furthermore, we found that beta-alanine inhibited the DNA synthesis (Additional file 11: Fig. S11D) and cell cycle progression (Additional file 11: Fig. S11E) and thus promoted the cell apoptosis (Additional file 11: Fig. S11F) and attenuated cell proliferation in both 5637 and T24 cells (Additional file 11: Fig. S11G). In addition, we noted that increased GARS abundance promoted the xenograft growth of T24 cells, whereas inhibition of K-Gly by beta-alanine delayed the xenograft growth in tumor cells, thereby validating the oncogenic role of K-Gly in the development of UC (Fig. 8J). Taken together, we found that upregulated expression levels of GARS promoted the UC progression through enhancing the pentose phosphate pathway by inhibiting activities of PGK1 and PKM2.

## Discussion

Here, we present a large-scale omics study on urothelial carcinoma of the bladder. Whole-genome sequencing, RNA sequencing, and proteomics, and phosphoproteomic data were generated as resource from a Chinese cohort of 116 patients. Our analysis provides a comprehensive insight into the molecular characterization of UC by encompassing somatic mutations, the mechanisms underlying NMIBC infiltrating into MIBC, proteomic subtypes, tumor microenvironment, and protein covariation networks capturing functional associations.

The genomic landscape revealed the consistency between our study and other studies, the frequent mutation rates of *TP53*, *ARID1A*, *FGFR3*, and *PIK3CA*, being a fine case in point. Copy number variations (CNVs) also act as an important driving force in several cancers. Proteomic characterization has provided valuable insight into CNA effects and their attenuation at the protein level, as CNA-mRNA correlations were significantly higher than CNA-protein correlations for genes. Notably, 139 significant cis-effects overlapping among mRNA, protein, and phosphoprotein levels were significantly enriched in positive regulation of GTPase activity, regulation of cell cycle, focal adhesion, and the ErbB signaling pathway, suggesting that core pathways activated in UC were affected by genomic aberrations. Furthermore, SND1 (chromosome 7) exerts a cis-effect on protein level and was associated with the activation of STAT3, which was relevant to tumor proliferation.

A deeper understanding of UC based on proteomics fills the gap between genome abnormalities and oncogenic protein machinery. Integrated proteogenomic characterization of tumors and MNUs revealed that some urothelium-specific proteins were less expressed in tumors than in MNUs, such as UPK family (UPK3A and UPK3BL among others). These proteins play an important role in urothelial bladder physiology functions [61], such as epithelial cell differentiation and urea transmembrane transport. Interestingly, phosphoproteomic analysis of tumors and morphologically normal human urothelium nominated UC-associated activated kinases, including CDK1 and PRKDC.

The two main categories of bladder cancer are NMIBC and MIBC. From a clinical standpoint, the progression from NMIBCs to MIBCs is the major determinant in the decision making leading to cystectomy [62]. Currently, markers that adequately predict the switch from NMIBCs to MIBCs are lacking. In this study, we found, for the first time, that 5p gain was associated with both poor prognosis (both OS and PFS). More importantly, when 5p gain occurs in NMIBCs, the survival rate dramatically decreases to a level that is comparable with that of MIBC, as opposed to WT NMIBC patients, who had a longer survival time. Therefore, our data revealed that 5p gain plays a role in the progression of NMIBC to MIBC, by modulating the actin cytoskeleton, which is implicated in tumor cell invasion.

Our study reports the first proteomic classification of UCs, and three proteomic subtypes that had distinct molecular features linked to the clinical, prognostic, and pathological features were identified. Comparisons between other classifications and our study showed a connection between proteomic and transcriptomic subgroups in the Eastern and Western countries. U-I and U-II, based on the proteomic subtypes, shared luminal features, whereas the U-III proteomic subtype presented with a more basal phenotype, as revealed by the transcriptomic classification. U-I and U-II contained the most NMIBCs, which is consistent with the results of another study, which characterized most NMIBC samples as luminal [63]. Furthermore, the phosphoproteome was applied in analyzing kinase features of proteomic subgroups. The results showed that ERBB3, SRC, and YES1 were activated in U-I, while CDK1 was activated in U-II, and PRKCB was activated in U-III. This observation suggests that ERBB3 and CDK1 inhibitors have the potential to be considered as therapeutical drugs for the luminal subtype, for which chemotherapy options are considered to be unsuitable. Taken together, these revealed that our proteome subgrouping was consistent with transcriptome-based subgrouping. Since proteins are the major executors of biological functions, the proteomic subgrouping reinforces previous transcriptome data and facilitates the discovery of variant proteins, which provide a resource for discovering potential biomarker candidates or therapeutic targets in the future.

Integrated proteogenomic analysis extensively characterized the immune landscapes of UCs. Further studies identified a number of potential therapeutic vulnerabilities, including IDO1 inhibition in immune-hot-tumors. We highlighted the specific association between 9q34.3 amplification and immune-hot phenotype, which suggested the activation of NF-κB1. Tumors showing high NF-κB1 activity are also enriched in a tumor-initiating cell expression signature [64]. In addition, the expression of PD-L1, an NF-κB1 target, was increased in patients carrying 9q34.3 amplification. These results suggest that patients with 9q34.3 amplification showed elevated expression level of PD-L1 and may benefit from therapy targeting PD-L1. Moreover, from follow-up, we found that two patients in our cohort were treated with Atezolizumab (a PD-L1 inhibitor) and showed favorable response (evaluated by clinical exports; Additional file 18: Table S6). Further investigation revealed that these two patients carried 9q34.3 amplification. Our data suggested the close relationship between PD-L1 and 9q34.3 amplification, which could be further verified in a larger cohort in the future.

PD-L1 expression, tumor mutational burden (TMB), and PD1 expression are known to be associated with the responses of immune checkpoint inhibitor (ICI) therapy, yet the relationship among them was found to be conflicting. Previous work conducted by Chen Y et al. has indicated that the relationship among the PD-L1 expression, TMB, and PD-1 expression showed demographically diversity. To be more specific, the significantly positive association between PD-L1 and PD-1 was only observed in Chinese patients [65]. In consistent with this previous research, in our study, we also did not observe significant correlation between the expression of PD-L1 or PD-1 with TMB (Spearman *R* = 0.0054, *P* = 0.97), while the significant positive correlation was found between the expression of PD-L1 and PD1. Our findings further confirm the diverse molecular features of Chinese population (Additional file 12: Fig. S12A–D).

Histological variants of UC are clinically significant at various levels, including diagnostic, prognostic, or therapeutic [56]. In our study, we explored the distinct features of histological variants in UC at protein and phosphoprotein levels which were not reported before. The results showed that papillary carcinoma was characterized by a low immune score, increased metabolism-related pathways, and the activated ERBB3/ERBB4 kinases. These results suggested that although immunotherapy is deemed unsuitable for patients with papillary carcinoma, they have the potential to be benefit from treatment with ERBB3/ERBB4 kinase inhibitors.

Our integrated analysis revealed that tRNA aminoacylation was higher in the subtype U-II. We found that GARS, not other types of aminoacyl-tRNA synthetases, was upregulated in tumor tissues. However, the mechanisms underlying the action of GARS in bladder cancer remain unclear. We found that GARS promote the bladder cancer progression in human urinary bladder carcinoma cell lines, T24 and 5637, through enhancing pentose phosphate pathway flux by inhibiting activities of PGK1 and PKM2. The enhanced pentose phosphate pathway supports DNA synthesis and rapid cell proliferation.

In summary, multi-omics integrative analysis is a valuable and powerful tool that provides a complementary and more comprehensive understanding of UC and offers an opportunity to expedite the translation of basic research to more precise diagnosis and treatment procedures at the clinical level. We believe that the broad usage of these data sets leads to new biological discoveries and generates useful therapeutic hypotheses.

## Conclusions

In summary, we firstly delineated the proteogenomic landscape of the Chinese UC. We found that 5p gain acts as a key factor participating in the progression from NMIBC to MIBC. Furthermore, proteomic-based classification of UC combined with multi-omics data revealed the molecular signatures of each subgroup and the subgroup-specific kinase. Analysis of the immune subtypes of UC revealed that 9q34.3 amplification was associated with the higher PD-L1 expression. Finally, we found that GARS promotes the bladder cancer progression in human urinary bladder carcinoma cell lines, T24 and 5637, through enhancing pentose phosphate pathway flux by inhibiting activities of PGK1 and PKM2. These results suggested that GARS could be a new therapeutic target in UC.

## Methods

### Sample selection

The UC samples and morphologically normal adjacent urothelium tissue samples used in this study were obtained from the Zhongshan Hospital, Fudan University. Patients, who did not undergo any anticancer treatments prior to surgery, were randomly selected from January 2011 to December 2017 upon their first visit. Primary tumor tissues and morphologically normal adjacent urothelium (MNU) tissues were surgically resected and formalin-fixed paraffin-embedded (FFPE). A total of 116 patients were collected based on the clinical information including gender, age, smoking status, nerve or vascular invasion, metastasis, hyperglycemia, hypertension, histological subtype, TNM staging (AJCC cancer staging system 8th edition), tumor purity, date of surgical resection, patients’ overall survival, and progressive-free survival time. All the clinical information is summarized in Additional file 13: Table S1 (Table 1).

FFPE tissue samples from 15 UC patients treated with anti-PD-L1 immunotherapy were included in the anti-PD-L1 cohort. The patients were categorized into 3 responders and 12 non-responders according to multidisciplinary radiologic evaluations. Samples were taken shortly before the initiation of the indicated treatment. All the clinical information is summarized in Additional file 18: Table S6. The study was approved by the Research Ethics Committees of Zhongshan Hospital (B2019-200R), and written, informed consent was provided by all patients.

### Sample preparation

The tissue specimens used were FFPE. The sample preparation followed FFomic strategy. Accurate evaluation of tumor cellularity was determined using the middle section of each tumor tissue block, which was resected and subjected to hematoxylin and eosin (H&E) staining. For proteomic, genomic, and phosphoproteomic sample preparation, slides (10 μm thick) were sectioned, deparaffinized with xylene, and washed in an ethanol gradient. Specimens selected according to H&E staining were scraped using a dissecting microscope and then stored at − 80 °C until needed. For RNA sample preparation, slides (10 μm thick) were sectioned, were not dewaxed, and stored at room temperature for further progressing. In addition, divergent histological variant tumors of one patient were scraped according to H&E staining. Tumor sections were required to contain an average of 70% tumor cell nuclei with equal to or less than 20% necrosis for inclusion in the study. Each sample was assigned a new research ID, and the patient’s name or medical record number used during hospitalization was de-identified.

### Pathology review

All samples were systematically evaluated to confirm the histopathological diagnosis and any variant histology according to the World Health Organization (WHO) classification by three expert genitourinary pathologists. Additionally, all tumor samples were assessed for tumor content, the presence and extent of tumor necrosis, and signs of invasion into the muscularis propria. Tumor samples were also evaluated for the presence and extent of inflammatory infiltrates, as well as for the type of the infiltrating cells (lymphocytes, neutrophils, eosinophils, histiocytes, plasma cells) in the tumor microenvironment. Any non-concordant diagnoses among the three pathologists were re-reviewed, and a resolution was reached following discussion. All the information is included in Additional file 13: Table S1.

### Whole-exome sequencing

#### DNA extraction

DNA from the tumor tissues and MNU tissues was extracted according to the manufacturer’s instructions of a QIAamp DNA Mini Kit (QIAGEN, Hilden, Germany). The quality of isolated and contaminated samples was verified using the following methods: (i) DNA degradation and contamination were monitored on 1% agarose gels; and (ii) DNA concentration was measured using Qubit® DNA Assay Kit in a Qubit® 2.0 Fluorimeter (Invitrogen, CA, USA).

#### Library preparation

An amount of 0.6 µg genomic DNA per sample was used as input material for the DNA preparation. Sequencing libraries were generated using an Agilent SureSelect Human All Exon kit (Agilent Technologies, CA, USA) following the manufacturer’s recommendations, following which index codes were added to each sample. Briefly, fragmentation was carried out using a hydrodynamic shearing system (Covaris, Massachusetts, USA) to generate 180–280 bp fragments. The remaining overhangs were converted into blunt ends via exonuclease/polymerase activities. Following adenylation of the 3’ ends of DNA fragments, adapter oligonucleotides were ligated. DNA fragments with ligated adapter molecules at both ends were selectively enriched in a PCR reaction. Following the PCR reaction, libraries were hybridized with the liquid phase via a biotin-labeled probe following which magnetic beads with streptomycin were utilized to capture the exons of genes. Captured libraries were enriched via a PCR reaction to add index tags in preparation for sequencing. Products were purified using an AMPure XP system (Beckman Coulter, Beverly, USA) and quantified using the Agilent high sensitivity DNA assay on the Agilent Bioanalyzer 2100 system.

#### Clustering and sequencing

Clustering of index-coded samples was performed on a cBot Cluster Generation System using a HiSeq PE Cluster Kit (Illumina) according to the manufacturer’s instructions. After cluster generation, the DNA libraries were sequenced on the Illumina HiSeq platform and 150 bp paired-end reads were generated.

### Whole-exome sequencing data analysis

#### Quality control

The original fluorescence image files obtained from the HiSeq platform were transformed to short reads (raw data) by base calling, following which these short reads were recorded in FASTQ format, which contains sequence information and corresponding sequencing quality information. Sequence artifacts, including reads containing adapter contamination, low-quality nucleotides, and unrecognizable nucleotides (N), undoubtedly set the barrier for the subsequent reliable bioinformatics analysis. Hence, quality control is an essential step that must be applied to guarantee meaningful downstream analysis.

The data processing steps were as follows:

- Paired reads were discarded if either read contained adapter contamination (> 10 nucleotides aligned to the adapter, allowing ≤ 10% mismatches).
- Paired reads were discarded if more than 10% of bases are uncertain.
- Paired reads were discarded if the proportion of low-quality (Phred quality < 5) bases is either read was over 50%.

All downstream bioinformatics analyses were based on high-quality clean data, which were retained after these steps. At the same time, QC statistics including total read number, raw data, raw depth, sequencing error rate, percentage of reads with Q30 (the percentage of bases with Phred-scaled quality scores greater than 30), and GC content distribution were calculated and summarized.

#### Reads mapping to reference sequence

Valid sequencing data were mapped to the reference human genome (UCSC hg19) using Burrows–Wheeler aligner (BWA) software [66] to obtain the original mapping results stored in BAM format. If one read, or one paired read, was mapped to multiple positions, the strategy adopted by the BWA was to choose the most likely placement. If two or more most likely placements were present, the BWA picked one randomly. Then, SAMtools [67] and Picard (http://broadinstitute.github.io/picard/) were used to sort BAM files and perform duplicate marking, local realignment, and base quality recalibration to generate final BAM files for computation of the sequence coverage and depth. The mapping step was very difficult due to mismatches, including true mutations and sequencing errors, and duplicates resulting from PCR amplification. These duplicate reads were uninformative and should not be considered as evidence for variants. We used Picard to mark these duplicates for the follow-up analysis.

#### Variant calling

Samtools mpileup and bcftools were used to perform variant calling and identify SNPs and InDels. Somatic SNP variant calls were assessed using MuTect [68], and the Indels variant calls were assessed using Strelka [69] with default options. The resulting somatic mutations were annotated using the ANNOVAR RefSeq gene-based annotation.

#### Copy number analysis

Copy number alterations (CNAs) were called by following the somatic CNA calling pipeline in GATK’s (GATK 4) Best Practice. The results of this pipeline and segment files of every 1000 were input in GISTIC2 [70], to identify significantly amplified or deleted focal-level and arm-level events, with a *Q* value < 0.1 considered significant. A log2 ratio cutoff 1 was used to define SCNA amplification and deletion. We further summarize the arm-level copy number change based on a weighted sum approach [71], in which the segment-level log2 copy ratios for all the segments located in the given arm were added up with the length of each segment being weighted. To exclude false positives as much as possible, relatively stringent cutoff thresholds were used with the following parameters: -ta 0.1 -tb 0.1 -brlen 0.98 -conf 0.9. Other parameters were the same as default values.

#### Co-occurrence and mutual exclusivity analysis of mutations

Co-occurrence and mutually exclusive mutated genes were detected using Fisher’s exact test in order to determine the co-occurrence and mutually exclusively of significantly mutated genes in our mutational dataset.

#### Analysis of significantly mutated genes

Filtered mutations (including SNV and indel) were further used to identify significantly mutated genes by MutSigCV (https://software.broadinstitute.org/cancer/cga/mutsig, version 1.4) with default parameters. Final MutSigCV P values were converted to *q* values using the method of Benjamini and Hochberg [72], and genes with *q* ≤ 0.1 were declared to be significantly mutated.

#### Mutation frequency in the Fudan cohort and previous UC studies

Mutation frequencies for previous UC studies in the TCGA cohort [73] were downloaded from the cBioPortal [74], while those in the Beijing cohort [19] were downloaded from the Beijing Institute of Genomics Data Center (https://bigd.big.ac.cn). The frequencies of all genes were compared with those from the Fudan cohort using Spearman’s correlation similarity matrix.

#### Mutational signature analysis using the Sigminer approach

Mutation signatures were jointly inferred for 113 tumors using the R package sigminer [75]. The sigminer approach (https://github.com/ShixiangWang/sigminer) was used to extract the underlying mutational signatures. The 96 mutation vectors (or contexts) generated by somatic SNVs based on six base substitutions (C>A, C>G, C>T, T>A, T>C, and T>G) within 16 possible combinations of neighboring bases for each substitution were used as input data to infer their contributions to the observed mutations. Sigminer using a nonnegative matrix factorization (NMF) approach was applied to decipher the 96 × 113 (i.e., mutational context-by-sample) matrix for the 30 known COSMIC cancer signatures (https://cancer.sanger.ac.uk/cosmic/signatures) and infer their exposure contributions.

#### Mutational signature analysis using the deconstruct Sigs approach

The mutational signature of each sample was deconstructed using the deconstructSigs approach [76] and its R package (deconstructSigs v1.8.0) with default parameters. Thirty COSMIC cancer signatures were considered, and their contributions (weights) in each patient were normalized between 0 and 1, and signatures with a weight below 0.08 were filtered out.

#### Functional annotation

Functional annotation is vital because the link between genetic variations and diseases is clarified by this process. ANNOVAR was performed to annotate the variant call format (VCF) obtained in a previous study [77]. The dbSNP, 1000 Genome, and other related databases were used to characterize the detected variants. Given the significance of exonic variants, gene transcript annotation databases, such as Consensus CDS, RefSeq, Ensembl, and UCSC, were also included in the determination of amino acid alterations. Annotation content contained the variant position, variant type, and conservative prediction, among others. These annotation results would help locate disease-causal mutants. The details of the annotation are provided in the supplementary material.

#### Tumor mutational burden

TMB was defined as the number of somatic mutations (including base substitutions and indels) in the coding region. Synonymous alterations were also counted [78]. To calculate the TMB, the total number of mutations counted was divided by the size of the coding sequence region of the Agilent SureSelect Human All Exon V6.

### Proteomic and phosphoproteomic analysis

#### FFPE protein extraction and trypsin digestion

Samples were lysed in TCEP buffer (2% deoxycholic acid sodium salt, 40 mM 2-chloroacetamide, 100 mM Tris–HCl, 10 mM Tris(2-chloroethyl) phosphate, 1 mM PFSM, pH 8.5) supplemented with protease inhibitors and phosphatase at 99 °C for 30 min. After cooling to room temperature, trypsin (Promega, Madison, WI, USA, #V5280) was added and digested for 18 h at 37 °C. 10% formic acid was added and vortexed for 3 min, followed by sedimentation for 5 min (12,000*g*). Next, a new 1.5-mL tube with extraction buffer (0.1% formic acid in 50% acetonitrile) was used to extract the supernatant (vortex for 3 min, followed by 12,000*g* of sedimentation for 5 min). Collected supernatant was transferred into a new tube for drying using a SpeedVac.

#### First dimensional reversed-phase separation for proteome

The dried tryptic peptides were re-dissolved in 10 mM NH_4_HCO_3_ (pH 10), vortexed for 3 min, and then centrifuged at 12,000*g* for 3 min. Peptides were separated in a home-made reverse-phase C18 column in a pipet tip with nine fractions using an increasing gradient of increasing acetonitrile (6%, 9%, 12%, 15%, 18%, 21%, 25%, 30%, and 35%) under basic conditions (pH 10). The nine fractions were combined into three fractions (6% + 15% + 25%, 9% + 18% + 30%, 12% + 21% + 35%), dried in a vacuum concentrator (Thermo Scientific), and then analyzed by mass spectrometry for proteomic profiling.

#### The enrichment of phosphorylated peptides

For phosphoproteomic analysis, slides (10 μm thick) from FFPE blocks were macro-dissected, deparaffinized with xylene, and washed with ethanol. Extracted tissues were lysed and digested with trypsin following the same protocol as previously described for “FFPE protein extraction and trypsin digestion.” Tryptic peptides were used for phosphopeptide enrichment using a High-Select Fe-NTA kit (Thermo Fisher Scientific, Rockford, IL, USA, #A32992) according to the kit manual and a previous report [79] with some modifications. In brief, peptides were suspended in binding/wash buffer (contained in the enrichment kit) and mixed with the equilibrated resins. The peptide–resin mixture was incubated for 30 min with three gentle blows at room temperature. Following incubation, the resins were washed thrice with binding/wash buffer and twice with water. The enriched peptides were eluted with elution buffer (contained in the enrichment kit) and immediately dried using a SpeedVac at 45 °C for mass spectrometry analysis.

#### Nano-LC–MS/MS analysis

For the proteome profiling samples, peptides were analyzed on a Q Exactive HF-X Hybrid Quadrupole-Orbitrap Mass Spectrometer (Thermo Fisher Scientific) coupled with a high-performance liquid chromatography system (EASY nLC 1200, Thermo Fisher Scientific). Dried peptide samples re-dissolved in Solvent A (0.1% formic acid in water) were loaded onto a 2-cm self-packed trap column (100 μm inner diameter, 3 μm ReproSil-Pur C18-AQ beads, Dr. Maisch GmbH) using Solvent A and separated on a 150-μm-inner-diameter column with a length of 15 cm (1.9 μm ReproSil-Pur C18-AQ beads, Dr. Maisch GmbH) over a 75-min gradient (Solvent A: 0.1% formic acid in water; Solvent B: 0.1% formic acid in 80% ACN) at a constant flow rate of 600 nL/min (0–75 min, 0 min, 4% B; 0–10 min, 4–15% B; 10–60 min, 15–30% B; 60–69 min, 30–50% B; 69–70 min, 50–100% B; 70–75 min, 100% B). Eluted peptides were ionized at 2 kV and introduced into the mass spectrometer. Mass spectrometry was performed in data-dependent acquisition mode. For the MS1 Spectra full scan, ions with m/z ranging from 300 to 1400 were acquired by an Orbitrap mass analyzer at a high resolution of 120,000. The automatic gain control (AGC) target value was set to 3E+06. The maximal ion injection time was 80 ms. MS2 spectral acquisition was performed in the ion trap in a rapid speed mode. Precursor ions were selected and fragmented with higher energy collision dissociation (HCD) with a normalized collision energy of 27%. Fragment ions were analyzed by an ion trap mass analyzer with an AGC target at 5E+04. The maximal ion injection time of MS2 was 20 ms. Peptides that triggered MS/MS scans were dynamically excluded from further MS/MS scans for 12 s.

For the phosphoproteomic samples, peptides were analyzed on a Q Exactive HF-X Hybrid Quadrupole-Orbitrap Mass Spectrometer (Thermo Fisher Scientific) coupled with a high-performance liquid chromatography system (EASY nLC 1200, Thermo Fisher Scientific). Dried peptide samples re-dissolved in Solvent A (0.1% formic acid in water) were loaded onto a 2-cm self-packed trap column (100 μm inner diameter, 3 μm ReproSil-Pur C18-AQ beads, Dr. Maisch GmbH) using Solvent A and separated on a 150-μm-inner-diameter column with a length of 30 cm (1.9 μm ReproSil-Pur C18-AQ beads, Dr. Maisch GmbH) over a 150-min gradient (buffer A: 0.1% formic acid in water; buffer B: 0.1% formic acid in 80% ACN) at a constant flow rate of 600 nL/min (0–150 min, 0 min, 4% B; 0–10 min, 4–15% B; 10–125 min, 15–30% B; 125–140 min, 30–50% B; 140–141 min, 50–100% B; 141–150 min, 100% B). The eluted phosphopeptides were ionized and detected by a Q Exactive HF-X Hybrid Quadrupole-Orbitrap mass spectrometry. Mass spectra were acquired over the scan range of m/z 300–1400 at a resolution of 120,000 (AUG target value of 3E+06 and maximum injection time 80 ms). For the MS2 scan, higher energy collision dissociation fragmentation was performed at a normalized collision energy of 30%. The MS2 AGC target was set to 5E+04 with a maximum injection time of 100 ms. The peptide mode was selected for monoisotopic precursor scan, and charge state screening was enabled to reject unassigned 1+, 7+, 8+, and > 8+ ions with a dynamic exclusion time of 40 s to discriminate against previously analyzed ions between ± 10 ppm.

### MS database searching

#### Peptide and protein identification

MS raw files were processed with a “Firmiana” (a one-stop proteomic cloud platform) [20] against the human National Center for Biotechnology Information (NCBI) RefSeq protein database (updated on 04-07-2013, 32,015 entries) using Mascot 2.4 (Matrix Science Inc., London, UK). The maximum number of missed cleavages was set to two. Mass tolerances of 20 ppm for the precursor and 50 mmu for production were allowed for Q Exactive HFX. The fixed modification was cysteine carbamidomethylation, while the variable modifications were N-acetylation and methionine oxidation. For the quality control of protein identification, the target-decoy-based strategy was applied to confirm that the false discovery rate (FDR) of both peptides and proteins was lower than 1%. The program percolator was used to obtain the probability value (*q* value) and showed that the FDR (measured by the decoy hits) of every peptide–spectrum match (PSM) was lower than 1%. All peptides shorter than seven amino acids were removed. The cutoff ion score for peptide identification was set at 20. All PSMs in all fractions were combined for protein quality control, which was a stringent quality control strategy. The *q* values of both target and decoy peptide sequences were dynamically increased employing the parsimony principle until the corresponding protein FDR was less than 1%. Finally, to reduce the false positive rate, proteins with at least two unique peptides were selected for further investigation.

#### Label-free-based MS quantification of proteins

The one-stop proteomic cloud platform, “Firmiana,” was further employed for protein quantification. The identification results and the raw data from the mzXML files were loaded. Then, for each identified peptide, the extracted-ion chromatogram (XIC) was extracted by searching against MS1 based on its identification information, and the abundance was estimated by calculating the area under the extracted XIC curve. For protein abundance calculation, the non-redundant peptide list was used to assemble proteins following the parsimony principle. Protein abundance was then estimated by a traditional label-free, intensity-based absolute quantification (iBAQ) algorithm, which divided protein abundance (derived from identified peptide intensities) by the number of theoretically observable peptides. Match between runs [80] was used to improve parallelism between tumor and MNU tissues from 116 patients. We built a dynamic regression function based on commonly identified peptides in tumor and non-tumor tissues. According to the correlation value, R2, Firmiana chooses a linear or quadratic function for regression to calculate the RT of the corresponding hidden peptides and check the existence of the XIC based on the m/z and calculated RT. The program evaluated the peak area values of the existing XICs. The peak area values were calculated as parts of the corresponding proteins. Proteins with at least two unique peptides with a 1% FDR at the peptide level were selected for further analysis. Then, the fraction of total (FOT), a relative quantification value that was defined as a protein’s iBAQ divided by the total iBAQ of all identified proteins in one experiment, was calculated as the normalized abundance of a particular protein among experiments. Finally, the FOT was further multiplied by 1E−5 for ease of presentation and FOTs less than 1E5 were replaced with 1E5 to adjust extremely small values.

#### Batch effect analysis

Hierarchical clustering, dip statistic test, and principal component analyses were implemented in R v.3.4.1 to assess batch effects in our proteome dataset with respect to the following two variables: batch identity and sample type (tumors and MNUs). For hierarchical clustering analysis, pairwise Spearman’s correlation coefficients of the 157 tumor samples that passed quality control were investigated. Samples of the same type exhibited high similarity, whereas samples of different types clearly differed. There was no clear association between batch identity and correlation coefficients. The density plot of the normalized intensities of the proteins identified in each sample showed that all samples passed quality control with an expected unimodal distribution (dip statistic test). The results of principal component analysis showed that batch effects were negligible for batch identity but significant for the sample types.

#### Quality control of the mass spectrometry data

For quality control of performance of mass spectrometry, the HEK293T cell (National Infrastructure Cell Line Resource) lysates were measured every 3 days to set the quality control standard. The quality control standard was digested and analyzed using the same method and conditions as the 20 samples. A pairwise Spearman’s correlation coefficient was calculated for all quality control runs in a statistical analysis environment R v.3.2.129, and the results are shown in Additional file 1: Fig. S1F. The average correlation coefficient among the standards was 0.90, while the maximum and minimum values were 0.93 and 0.82, respectively.

### RNA-Seq

#### RNA extraction

RNA was extracted from tissues by using the RNAstorm™ FFPE kit (CELLDATA, USA, #CA94538) according to the manufacturer’s protocol. RNA integrity and concentration were determined using ﻿a NanoDrop 8000 spectrophotometer (Thermo Fisher Scientific). For library preparation of RNA sequencing, a total amount of 500 ng RNA per sample was used as input material for RNA sample preparations. Sequencing libraries were generated using a Ribo-off® rRNA Depletion Kit (H/M/R) (Vazyme, Nanjing, China, #N406) and a VAHTS® Universal V6 RNA-seq Library Prep Kit for Illumina (#N401-NR604) following the manufacturer’s recommendations. Index codes were added to attribute sequences to each sample. The libraries were sequenced on an Illumina platform and 150 bp paired-end reads were generated.

#### RNA-Seq data analysis

RNA-seq raw data quality was assessed using FastQC (v0.11.9), and the adaptor was trimmed with Trim_Galore (version 0.6.6) before any data filtering criteria were applied. Reads were mapped onto the human reference genome (GRCh38.p13 assembly) using STAR software (v2.7.7a). The mapped reads were assembled into transcripts or genes by using StringTie software (v2.1.4) and the genome annotation file (hg38_ucsc.annotated.gtf). For quantification purpose, the relative abundance of the transcript/gene was measured using the normalized metrics, FPKM (fragments per kilobase of transcript per million mapped reads). Transcripts with an FPKM score above one were retained, resulting in a total of 32,873 gene IDs. All known exons in the annotated files were 100% covered.

### Quantification and statistical analysis

#### Missing value imputation

For the proteomic and phosphoproteomic data, FOTs multiplied by 1E5 were used for quantification, and missing values were imputed with 1E−5 and finally, log2-transformed, if necessary.

#### Differential protein analysis

Proteins that were expressed in more than 30% of the samples were selected for differential expression analysis. The Wilcoxon rank-sum test was used to examine whether proteins were differentially expressed between tumors (*n* = 157 samples) and MNUs (*n* = 75 samples), NMIBCs (*n* = 45 samples) and MIBCs (*n* = 71 samples), or patients with different mutation statuses and CNA of statuses. Upregulated or downregulated proteins are defined as proteins differentially expressed in one group compared with the other group (Wilcoxon rank-sum test, BH *p* < 0.05, T/N > 2 or < 1/2). The same strategy was applied to the differential expression analysis of phosphoproteomic data and RNA-seq data.

#### Pathway enrichment analysis

Differentially expressed genes were subjected to gene ontology and KEGG pathway enrichment analysis in DAVID [81] with a *p* value/FDR < 0.05. We used gene sets of molecular pathways from the KEGG [82]/Hallmark [83]/Reactome [84]/GO [85] databases to compute pathways.

#### Pathway scores and correlation analysis

Single-sample gene set enrichment analysis (ssGSEA) [86] was utilized to obtain pathway scores for each sample based on RNA-seq, proteomic, and phosphoproteomic data using the R package GSVA [87]. Correlations between the pathway scores and other features were determined using Spearman’s correlation. Inferred activity was performed using ssGSEA implemented in the R package GSVA with a minimum gene set size of 10. The transcriptional targets of STAT3 transcription factors were collected from the ENCODE Project Consortium [88] and used to infer STAT3 activity via ssGSEA. Transcriptional targets of NFKB1 transcription factors were collected from the ENCODE Project Consortium [88] and used to infer NFKB1 activity by using ssGSEA.

#### Candidate target of plasma membrane protein

The following three criteria were used to identify plasma membrane protein with prognostic power: 1) The candidate proteins were expressed in more than 80% of the 157 tumor samples; 2) the candidates were expressed at least twofold higher in tumors than the MNUs (Wilcoxon rank-sum test, BH *p* < 0.01); and 3) the high expressions of candidates were negatively correlated with the overall survival (Kaplan–Meier analysis, log-rank test, *p* < 0.05).

## Phosphoproteomic data analysis

### Database searching of MS phosphoproteomic data

Phosphoproteome MS raw files were searched against the human RefSeq protein database (27,414 proteins, version 04/07/2013) using Proteome Discoverer (version 2.3.0.523) with a Mascot [89] (version 2.3.01) engine with a percolator [90]. Carbamidomethyl cysteine was used as a fixed modification, and oxidized methionine, protein N-term acetylation, and phospho (S/T/Y) were set as variable modifications. The false discovery rate (FDR) of peptides and proteins was set at 1%. The tolerance for spectral searches a mass tolerance of 20 ppm for the precursor. The maximum number of missing cleavage site was set at 2. For phosphosite localization, ptmRS [91] was used to determine phosphosite confidence and a phosphosite probability > 0.75 was used for further analysis.

#### Kinase activity prediction

To estimate changes in kinase activity, we performed kinase enrichment analysis on significantly differentiated phosphosites in tumors compared to MNUs, for MIBC and NMIBC or each subtype via kinase–substrate enrichment analysis (KSEA) [92]. Known kinase–substrate site relationships from PhosphoSitePlus (PSP) [93] or NetworKIN 3.0 [94] with scores greater than 1 were used for kinase–substrate analysis. A kinase score was given for each kinase based exclusively on the collective phosphorylation status of its substrates and transformed into a z-score. For kinase enrichment analysis, the threshold used for significantly enriched kinases was *p* < 0.05.

## mRNA, proteomic, and phosphoproteomic subgrouping analysis

### Consensus clustering analysis

Prior to clustering analysis, proteins that were expressed in more than 30% of patient samples were selected (*n* = 5489). To identify new proteomic subtypes of UC, consensus clustering (R package ConsensusClusterPlus v.1.48.0) [95, 96], and a reconciling clustering algorithm with a resampling technique, was conducted on 5489 proteins. The clustering algorithm was k-means using Euclidean distance. Consensus Cluster Plus parameters were reps = 1000, pItem = 0.8, pFeature = 1, clusterAlg = “pam,” distance = “spearman,” plot = “PDF” Euclidean distance and 1.00 resampling repetitions in the range of 2–10 clusters. The consensus matrices for *k* = 3, 4, and 5 clusters are shown in Additional file 5: Fig. S5A. A consensus matrix with *k* = 3 appeared to yield the clearest cut between clusters and showed a significant association with the patient survival. The same strategy was applied to the phosphoproteomic data and RNA-seq data.

#### Subtype-specific expressed proteins analysis

The 5489 proteins used for consensus clustering were subjected to differential expression analysis among the three subtypes using the Kruskal–Wallis test. Upregulated or downregulated proteins were defined as subtype-specific expressed proteins (Kruskal–Wallis test, *p* < 0.05; one subtype/other subtype > 1.5 or < 2/3) and used for subgroup-specific pathway analysis. The same strategy was applied to the phosphoproteomic data and RNA-seq data for subtype-specific expression analysis.

#### Comparison of the UC-proteomic subgrouping with previous UC subgroupings

We applied our subtype classifier to an mRNA data set from TCGA, resulting in three clusters (U-I, U-II, and U-III) being obtained from the TCGA cohort.

#### Correlation between proteomic subtypes and clinical features

For the purpose of measuring correlations between proteomic subtypes and clinical features, Fisher’s exact test was performed on categorical variables, including driver gene mutations, chromosome 5p gain, gender, age-group, smoke status, nerve invasion, vascular invasion, metastasis, hyperglycemia, hypertension, TNM stage, and histological type.

#### WGCNA analysis

Weighted gene correlation network analysis (WGCNA) [97] was used to identify groups of co-regulated genes in an unsupervised manner. We input 6692 proteins present in more than 10% of the 116 patients into WGCNA. A sample network was constructed to identify outlying samples with a standardized connectivity score of less than − 2.5 [98]. A signed gene co-expression network was constructed with a soft threshold power of 10. Groups of co-regulated genes (modules) correlated with each other with a Pearson correlation coefficient of 0.9, or better, were merged. Pathway enrichment analysis was used for the functional annotation of the identified modules (*n* = 16). The eigengenes of each module were used to measure the association between modules and clinical information.

#### Survival analysis

Kaplan–Meier survival curves (log-rank test) were used to determine the overall survival (OS) and progression-free survival (PFS) of proteomic subtypes and patients. The coefficient value, which is equal to ln (HR), was calculated using Cox proportional hazards regression analysis. *p* values less than 0.05 were considered significantly different and selected for Cox regression multivariate analysis. Prior to the log-rank test of a given protein, phosphoprotein, or phosphosite, survminer (version 0.2.4, R package) with maxstat (maximally selected rank statistics; http://r-addict.com/2016/11/21/Optimal-Cutpoint-maxstat.html) was used to determine the optimal cutoff point for the selected samples according to a previous study [99]. OS curves were then calculated (Kaplan–Meier analysis, log-rank test) based on the optimal cutoff point.

## Multi-omics data analysis

### Effects of copy number alterations

SCNAs affecting mRNA and protein/phosphoprotein abundance in either “cis” (within the same aberrant locus) or “trans” (remote locus) mode were visualized by multiOmicsViz (R package) [100]. Spearman’s correlation coefficients and associated multiple-test adjusted *p* values were calculated for all CNA–mRNA pairs for 16,274 genes, resulting in CNA-protein pairs for 8987 genes and CNA-phosphoprotein pairs for 5147 genes, respectively.

#### mRNA–protein correlation in tumors and MNUs

The Spearman correlation coefficients of genes/proteins were calculated for those that were detected in more than 30% of the tumors (5001 genes in 43 samples) or MNUs (3983 genes in 22 samples) in both RNA-Seq and MS data. Functional pathways were enriched by gene set enrichment analysis (GSEA) enrichment analysis using the correlation-ranked list of proteins.

#### Defining cancer-associated genes

Cancer-associated genes (CAG) were compiled from genes defined by Bailey et al. [101] and cancer-associated genes listed in Mertins et al. [102] and adapted from Vogelstein et al. [103].

#### Gene Set Enrichment Analysis (GSEA)

GSEA was performed by the GSEA software (http://software.broadinstitute.org/gsea/index.jsp). Gene sets including KEGG, GO Biological Process (BP), Reactome, and HALLMARK downloaded from the Molecular Signatures Database (MSigDB v7.1, http://software.broadinstitute.org/gsea/msigdb/index.jsp) were used.

#### Immune subtype analysis

The abundances of 64 different cell types for UCs were computed via xCell using protein expression values [104]. Additional file 18: Table S6 contains the final score computed by xCell of different cell types for tumor samples. Consensus clustering on xCell signatures was performed in order to identify groups of samples with the same immune/stromal characteristics. Consensus clustering was performed using the R package ConsensusClusterPlus [95]. For estimating tumor purity, immune and stromal scores were determined based on proteomic data using the R package GSVA [87]. ssGSEA was utilized to obtain pathway score based on proteomic data using the R package GSVA. A one-versus-all test was used to select cell types in different immune cluster, and a Wilcoxon rank-sum test was performed subsequently to find pathways differentially expressed between cold-tumor- and hot-tumor-enriched subgroup. Additional file 18: Table S6 shows genes/proteins and pathways differentially expressed based on RNA-seq and proteomic abundance.

#### Immunohistochemistry (IHC)

Formalin-fixed, paraffin-embedded tissue sections of 10 µM thickness were stained in batches for detecting CYP2J2, MLH1, PRKCB, TRAF2, PD-L1 in a central laboratory at the Zhongshan Hospital according to standard automated protocols. Deparaffinization and rehydration were performed, followed by antigen retrieval and antibody staining. CYP2J2, MLH1, PRKCB, TRAF2, and PD-L1 IHC were performed using the Leica BOND-MAX auto staining system (Roche). Rabbit monoclonal anti-CYP2J2 antibody (Proteintech Group, #13562-1-AP), anti-MLH1 antibody (Proteintech Group, #11697-1-AP), anti-PRKCB (Proteintech Group, #12919-1-AP), anti-TRAF2 (Proteintech Group, #26846-AP), and anti-PD-L1 antibody (Abcam ab205921) were introduced, followed by detection with a Bond Polymer Refine Detection DS9800 (Bond). Slides were imaged using an OLYMPUS BX43 microscope (OLYMPUS) and processed using a ScanScope (Leica).

## Functional experiments

### Antibodies and reagents

Primary antibodies used in this study included GARS (Proteintech Group, Rosemont, USA, #15831-1-AP), AARS2(Proteintech Group, #22696-1-AP), TARS (Proteintech Group, #67828-1-Ig), SARS (Proteintech Group, #15162-1-AP), p-4E-BP (Thr37/46, Cell Signaling Technology, Danvers, USA, #2855), 4E-BP (Cell Signaling Technology, #9644), p-S6K (Cell Signaling Technology, #9202), S6K (phospho T389 + T412, Abcam, Cambridge, UK, #ab60948), K-Gly, Actin (GenScript, New Jersey, USA, #A00702), Flag (Abmart, #M20008), Myc (Abmart, Shanghai, China, #M20003), PGK1(Wuhan Fine Biotech, Wuhan, China, #FNab06354), and PKM2(Cell Signaling Technology, #4053).

#### Cell culture

Human T24 cells and 5637 cells were kindly provided by the Cell Bank of the Chinese Academy of Science (Shanghai, China). Human T24 cells were cultured in McCoy's 5a medium (Invitrogen) containing 10% fetal bovine serum (Invitrogen, Carlsbad, CA, USA), 100 units/mL penicillin (Invitrogen), and 100 μg/mL streptomycin (Invitrogen). The 5637 cells were cultured in RPMI-1640 medium (Invitrogen) containing 10% fetal bovine serum (Invitrogen, Carlsbad, CA, USA), 100 ug/mL penicillin (Invitrogen), and 100 μg/mL streptomycin (Invitrogen). The cells were incubated in a 5% CO_2_ atmosphere at 37 °C. Cell transfection was performed using Lipofectamine 3000 (Invitrogen).

#### RNA interference

Synthetic oligos were used for siRNA-mediated silencing of GARS, and scrambled siRNA was used as a control. Cells were transfected with siRNAs using Lipofectamine 3000 according to the manufacturer’s protocol. Knockdown efficiency was verified by western blotting. The siRNA sequences were as follows: GARS, 5′-CTTGAGACCAGAAACTGCA-3′.

#### Proliferation assay

Cell proliferation was assessed using a Cell Counting Kit-8 (Dojindo Laboratories, Kumamoto, Japan). In brief, cells were seeded in a 96-well plate at a density of 4 × 10^3^ cells per well and allowed to adhere. Cell Counting Kit-8 solution (10 μL) was added to each well, and the cells were cultured in 5% CO_2_ at 37 °C for 2 h. Cell proliferation was determined by measuring the absorbance at 450 nm.

#### In vivo xenograft studies

Four- to six-week-old BALB/c nude mice were obtained (Shanghai SLAC Laboratory Animal Co., Ltd., Shanghai, China) for in vivo xenografts. Control cells and T24/5637 cell lines stably overexpressing GARS shRNA were subcutaneously heterotransplanted into the left and right flanks of each mouse, respectively. For beta-alanine treatment, 100 mg/kg beta-alanine (Cat no. 107959, Sigma, Inc.) was intraperitoneally injected into the abdominal cavities of the animals twice a week. The mice were maintained under the specified conditions. At the end of the experiment, the tumors were excised, weighed, and imaged. All procedures were performed with the approval of the Animal Care Committee of the Fudan University.

#### NMR measurement

Dried extracts were reconstituted in 570 μL of phosphate buffer (0.15 M, K_2_HPO_4_-NaH_2_PO_4_, pH 7.43) containing 80% D_2_O (v/v) and TSP (0.2915 mM). The mixture was then centrifuged at 16,099*g* for 10 min at 4 °C. Next, 530 μL of each supernatant was transferred into a standard 5 mm NMR tube for analysis. All the one-dimensional 1H NMR spectra were acquired at 298 K on a Bruker Advance III 600 MHz NMR spectrometer (600.13 MHz for proton frequency) equipped with a quaternary cryogenic inverse probe (Bruker Biospin, Germany) using the first increment of the gradient selected NOESY pulse sequence (NOESYGPPR1D). Then, 64 transients were collected into 32 k data points with a spectral width of 20 ppm for each sample. The total relaxation delay time to obtain completely relaxed NMR spectra was 26 s. All NMR spectra were processed using the TOPSPIN software package (version 3.6.0, Bruker Biospin, Germany). For 1H NMR spectra, an exponential window function was employed with a line broadening factor of 1 Hz and zero-filled to 128 K prior to Fourier transformation. Each spectrum was then phase- and baseline-corrected manually with the chemical shift referenced to TSP (δ 0.00). The spectral regions were then integrated into bins with a width of 0.002 ppm (1.2 Hz) using the AMIX software package (version 3.8.3, Bruker Biospin). The absolute concentration of metabolites was calculated using the known concentration of TSP.

#### PKM2 and PGK1 enzymatic activity assays

After transfection, cells were cultured for 48 h. Then, the cells were collected in a centrifuge tube and the supernatant was discarded after centrifugation. Next, 1 mL of extracting solution was added to 5 × 106 cells, and the cells were destroyed using ultrasound (ice bath; 200 W; ultrasound 3 s per time; 10 s interval; repeated 30 times). After centrifugation at 8000*g* at 4 °C for 10 min, the supernatant was collected and placed on ice for measurement. A pyruvate kinase assay kit (BC0540, Solarbio, China) was used to determine PKM2 enzymatic activity. The working solution was real-time-prepared according to manufactures’ protocol. To detect PGK1 enzymatic activity, a reaction buffer containing 50 mM Tris–HCl (pH 7.6), 8 mM MgCl_2_, 4 mM ATP, 0.2 mM NADH, 12 mM 3-phosphoglycerate, and 8 U GAPDH at room temperature was used. An aliquot of 10 μL of the cell lysate was used for these assays. The change in absorbance was measured at 340 nm wavelength.

### Statistical analysis

Standard statistical tests were used to analyze the clinical data, including but not limited to Student’s *t* test, Wilcoxon rank-sum test, chi-square test, Fisher’s exact test, Kruskal–Wallis test, and log-rank test. For categorical variables versus categorical variables (including driver gene mutations, chromosome 5p gain, gender, age-group, smoke status, nerve invasion, vascular invasion, metastasis, hyperglycemia, hypertension, TNM stage, and histological type), Fisher’s exact test was used in a 2 × 2 table, otherwise chi-square test was used. The Wilcoxon rank-sum test was used to examine whether genes were differentially expressed between tumors (*n* = 157 samples) and MNUs (*n* = 75 samples), NMIBCs (*n* = 45 samples), and MIBCs (*n* = 71 samples), or patients with different mutation statuses and CNA of statuses. The Kruskal–Wallis test was used to test whether genes were differentially expressed among the three proteomic subtypes or other subgroups. A one-versus-all test was used to select cell types in different immune cluster. To account for multiple-testing, the P values were adjusted using the Benjamini–Hochberg FDR correction. Kaplan–Meier plots (log-rank test) were used to describe overall survival. Variables associated with overall survival and progression-free survival were identified using univariate Cox proportional hazards regression models. Significant factors in univariate analysis were further subjected to a multivariate Cox regression analysis in a forward LR manner. All the analyses of clinical data were performed in R (version 3.4.3). For functional experiments, three biological repeats were performed independently, and results were expressed as mean ± standard error of the mean (SEM). The statistical significance of differences was determined by two-way ANOVA. Statistical analysis was performed using GraphPad Prism (version 5.01). The p values less than 0.05, 0.01, 0.001, and 0.0001 were marked with *, **, ***, and ****, respectively. All the statistical analysis had been checked by two statisticians.

## Supplementary Information

**Additional file 1. Fig. S1**: Multi-omics landscape of UC samples. Related to Figure 1. (A) Clinical data of our cohort. (B) Left: Sequencing depths of WES for tumors tissues and MNU tissues. Right: All input reads in RNA-seq for tumors tissues and MNU tissues. (C) The four mutational signatures identified in TCGA cohort by Sigminer analysis. (D) VENN plot of signatures identified among different cohort. (E) Proteins identified in tumor tissues and MNU tissues. (F) Cumulative number of protein identifications. Blue presents MNU samples; red presents tumor tissues. (G) Dynamic ranges of identified protein abundances identified in tumor tissues and MNU tissues. (H) The numbers of proteins identified in MNU tissues (blue) and tumor tissues (red). (I) Correlated matrix of 233 sample proteomes (Spearman’s correlation coefficients). Red color indicates high correlation; blue color indicates low correlation. (J) Distribution of protein abundances in tumors and MNUs by density plot. A unimodal distribution (dip test) was observed. All samples passed proteomic quality control. (K) Number of phosphoproteins (left) and phosphosites identified in tumors and MNUs. (L) Quality control of mass spectrometry using tryptic digest of 293T cells. The top-left half of the panel represents the pairwise Spearman’s correlation coefficients of samples and the bottom-right half of the panel depicts the pairwise scatter plots from sample comparison. (M) Number of genes identified in tumors and MNUs using RNA-seq. (N) The overlap of genes identified in the proteome, phosphoproteome and transcriptome. Top: MNU tissue, bottom: tumor tissue.

**Additional file 2. Fig. S2**: Impact of copy number alteration on mRNA and protein expression. Related to Figure 2. (A) Functional impacts of CNA on phosphoprotein. Top panel: positive and negative correlations were indicated by red and blue colors, respectively. Bottom panel: number of phosphoproteins that were significantly associated with a specific CNA. (B) Pathways enriched for respective specific *cis*-effects. (C) 10 CAGs and pathways they affected in TCGA cohort. (D) Arm-level CNAs in TCGA cohort and Beijing cohort. Red denotes amplification and blue denotes deletion. (E) Compared arm-level CNAs among different cohorts. (F) Genome-wide focal amplifications and deletions. Chromosomal locations of peaks of significantly recurring focal amplifications (red) and deletions (blue) were filtered by FDRs. Peaks were annotated with candidate driver oncogenes or tumor suppressors.

**Additional file 3. Fig. S3**: Impact of copy number alteration on mRNA and protein expression. Related to Figure 2. (A) Left: Kaplan–Meier curves for progression-free survival based on the chromosome 5p gain and WT groups. Right: Kaplan–Meier curves for progression-free survival based on the chromosome 7q gain and WT groups. The p value was calculated by log-rank test. 95% confidence interval was also presented. (B–C) Volcano plot showing the cis-effect genes on chromosome 5p (B) and 7q (C). The dots represent proteins; the triangles represent mRNA. The p values were calculated by Spearman's correlation test. (D–E) The expression status of the SND1 gene in different cancer subtypes from the TCGA dataset using TIMER2 (@@@timer.cistrome.org/) (E) and protein abundance of SND1 in tumor and MNU groups (D). The p values were calculated by Wilcoxon rank-sum test. * P < .05; ** P < .01; *** P < .001. (F–G) Expression levels of the SND1 were analyzed by the main pathological stages of the BLCA TCGA cohort (F) and our cohort (G) Log2 (TPM + 1) was applied for log-scale (p value from Kruskal–Wallis test). (H) the correlation between enriched cell cycle pathway and SND1 protein abundance. (I–J) Correlation of SND1 protein abundance with MCM2 protein change (I) and the correlation between SND1 and STAT3 in TCGA BLCA cohort (J) (p value from Spearman's correlation test). (K) Correlation between SND1 protein abundance and estimated STAT3 activity (p value from Spearman's correlation test). (L) Overall survival analyses of UC patients with high or low levels of STAT3 protein abundance (p value from log-rank test). (M) Correlation of SND1 protein abundance with STAT3 phosphorylation change (phosphoprotein).

**Additional file 4. Fig. S4**: Integrative analyses of transcriptomic, proteomic, and phosphoproteomic Data in UC Samples. Related to Figure 3. (A–C) Principal component analysis (PCA) of proteomic data (5,546 proteins) (A), phosphoproteomics (1,672 phosphoproteins) (B), and RNA-Seq (27,829 genes) (C) between tumors and MNUs. Red dots: tumors; blue dots: MNUs. (D–F) Proteins (D), genes (RNA-Seq) (E), and phosphoproteins (F) abundance differences between tumors or MNUs (p from Wilcoxon rank-sum test). (G) VENN plot of urothelial cancer-type-specific proteins identified between Zhou's cohort and Fudan cohort. (H–J) Pathways enriched for differentially expressed genes (H), proteins (I), and phosphoproteins (J) in tumors and MNUs. (K) Representative proteins from one of the four biological pathways and their association with prognosis (overall survival) (p value from log-rank test). (L–M) Proportions of urothelial bladder tumors with high, medium, or low staining, or not detected (ND) as reported by the Human Protein Atlas (HPA) (L) and tumor-cell specific immunohistochemistry (IHC) staining scores defined by the HPA (M). (N) IHC images for PKN1 proteins from HPA (left) and their association with clinical outcomes (overall survival) in all patients (*p* value from log-rank test).

**Additional file 5. Fig. S5**: Proteogenomic analysis between NMIBC and MIBC. Related to Figure 4. (A–C) H&E-stained slides of low-grade NMIBC (A), high-grade NIMBC (B), and MIBC tissues (C). (D–E) PCA of RNA-Seq (27,752 genes) (D) and phosphoproteomics (2,014 phosphoproteins) (E) between NMIBC and MIBC. (F–G) Genes (RNA-Seq) (F) and proteins (G) abundance differences between NMIBC and MIBC (Wilcoxon rank-sum test). (H–J) Pathways enriched for differentially expressed proteins (H) genes (RNA-Seq) (I) and phosphoproteins (J) in NMIBC and MIBC. (K–L) Line plots and boxplots of proteins involved in the crucial pathways from low-grade NMIBC, high-grade MIBC to MIBC. (M–N) Left, heatmap of protein abundance among low-grade NMIBC, high-grade MIBC, and MIBC. Middle, prognostic risk scores (overall survival) of each protein. The middle red points indicate log2-based hazard ratio for each protein; endpoints represent lower or upper 95% confidence intervals. Right, biological function category of the proteins. (O) Volcano plot showing the correlation between enriched Gene Ontology biological processes and TRIO protein abundance. (P) Correlation between TRIO and RHOG in the TCGA BLCA cohort. (Q) Correlation between RHOG mRNA abundance and ROCK1 mRNA abundance. The p values in (O)–(Q) were calculated by Spearman's correlation test.

**Additional file 6. Fig. S6**: Proteomic subtypes of UC and signature proteins. Related to Figure 5. (A) Consensus matrices of identified clusters (*K*=3), top: Clusters based on proteomic analysis data; median: clusters based on RNA data; bottom: clusters based on phosphoproteomic data. (B) Forest plot for multivariate Cox regressions for papillary, stage, never invasion, vascular invasion, gender HBP, hyperglycemia, smoking, age, and proteomic subtype, with the reference variable for each covariate set to the best-survival subtype. The main effects are presented as hazard ratios with 95% confidence intervals. (C) Signature proteins of each proteomic subtype and their association with prognosis (overall survival) (p value from log-rank test). (D) Gene expression signature scores. (Kruskal–Wallis test, p < 0.05). (E) Proteins that were differentially expressed in the three proteomic subtypes. (F) IHC profiling of proteomic subtype markers in UC. FFPE sections were stained for CYP2J2, MLH1 and PRKCB protein markers in UC tumor tissues. The scale bar indicates 100 μm.

**Additional file 7. Fig. S7**: Proteomic subtypes of UC and signature proteins. Related to Figure 5. (A) Distributions of main COSMIC signatures in the proteomic subtypes across 116 UC patients. Right: Heatmap of the comparison between different proteomic subtypes. (B) Association of mutational signatures with clinical features (Wilcoxon rank-sum test, p < 0.05). (C) Evaluation of kinase activities by KSEA in tumors across the three proteomic subtypes. (D) Mutational hotspots of FGFR3 in this study. (E) Mutational hotspots of FGFR3 in TCGA cohort.

**Additional file 8. Fig. S8**: Immune cell infiltration in UC tumors. Related to Figure 6. (A) Comparison between RNA-seq and global proteomics for estimating immunity (right) and stromal cell (left) infiltration based on 43 UC tumor samples. (B) Kaplan–Meier curves for progression-free survival of different immune clusters (p value from log-rank test). (C) Volcano plot showing the correlation between enrichment pathway and TRAF2 protein Abundance. (D) Scatter plot showing the correlation between the mRNA abundance change of TRAF2 and the mRNA abundance change of TNF. (E) Scatter plot showing the correlation between the mRNA abundance changes in TRAF2 and TNFRSF1B. (F) Correlation between average mRNA abundance and estimated NFKB1 activity change. (G) Scatter plot showing the correlation between the mRNA abundance changes in TRAF2 and CD274. (H) Correlation between TRAF2 mRNA abundance and CD274 protein change changes in the TCGA BLCA cohort. (I) Scatter plot showing the correlated between the mRNA abundance change of TRAF2 and CD8 enrichment score. (J) TRAF2 identified in patients with PD-L1-positive by IHC in Fudan cohort. The scale bar indicates 100 μm. (K) IHC of TRAF2 and PD-L1 in PD-L1-response and non-response. The scale bar indicates 100 µm.

**Additional file 9. Fig. S9**: Clinical outcomes associated with proteomic and phosphoproteomic profiles. Related to Figure 7. (A) Clustering dendrogram of samples based on their Euclidean distance. All samples are located in the clusters and also passed the cutoff thresholds. (B) Clustering dendrogram of genes, with dissimilarities based on topological overlap, together with assigned module colors. (C) left: the scale-free fit index (y-axis) as a function of the soft-thresholding power (x-axis); right: mean connectivity (degree, y-axis) as a function of soft-thresholding power (x-axis). (D–E) Clinical features (Serum albumin value and urea value) associated with prognosis (overall survival) (p value from log-rank test). (F) Representative proteins from the blue module and their association with prognosis (overall survival) (p value from log-rank test). (G) IHC images for GARS, CAV1, PH4A2, PLOD1, FLNA, and PRKG1 proteins in HPA database. (H) The distribution of different histological variations. (I) Pathways were enriched in different histological variations, based on phosphoproteome (Wilcoxon rank-sum p < 0.05).

**Additional file 10. Fig. S10**: GARS promotes bladder cancer cell proliferation through non-canonical function. Related to Figure 8. (A)–(B) DNA synthesis. Scale bar=100 µm. (C)–(D) Cell cycle progression. (E)–(F) in T24 and 5637 cell lines subjected to GARS-overexpressing or knocking down. Scale bar=100 µm.

**Additional file 11. Fig. S11**: GARS promotes bladder cancer cell proliferation through non-canonical function. Related to Figure 8. (A) Metabolite levels of tumor and normal tissues of bladder cancer, as detected by NMR. (B) Levels of PGK1 and PKM2 in both 5637 and T24 GARS-overexpressing cells. (C) The structural analogs of glycine. (D–G) The effects of beta-alanine on DNA synthesis (D), cell cycle progression (E), cell apoptosis (F), and slowed down the cell proliferation (G), in both 5637 and T24 cells; Scale bar=100 µm.

**Additional file 12. Fig. S12**: Relationships among PD-L1 expression, PD-1 expression, and TMB. (A) Correlation analysis between PD-L1 expression and TMB in proteomic data. (B) Correlation analysis between PD-L1 expression and TMB in mRNA data. (C) Correlation analysis between PD-1 expression and TMB in mRNA data. (D) Correlation analysis between the expression of PD-L1 and PD-1 in mRNA data. The p values (A)–(D) were calculated by Spearman's correlation test.

**Additional file 13. Table S1**: Clinicopathologic information and multi-omics data in UC cohort. Related to Figure 1, Additional file 1: Fig. S1. A. Clinical data of UC samples. B. The sheet contains information of mutations identified by WES in the 116 UC. C. Average sequencing depth on target. D. Significantly mutated genes identified by MutSig. E. Genome signature analysis. F. RNA clean reads. G. Genes identified at protein level (unique peptide ≥2). H. RNA-protein pairs. I. Phosphosites identified in UC samples. J. HEK293T used for QC.

**Additional file 14. Table S2**: CNV information in UC cohort. Related to Figure 2, Additional file 2: Fig. S2. A. This sheet contains somatic copy number gains and the relevant genes. B. This sheet contains somatic copy number losses and the relevant genes. C. List of cancer-associated genes (CAG), see Methods “Defining cancer-associated genes (CAG).” D. Lists of significant positive cis-effects in CNA-Protein and CAN-mRNA of chromosome 5p. E. Lists of significant positive cis-effects in CNA-Protein and CAN-mRNA of chromosome 7q.

**Additional file 15. Table S3**: Differentially expressed mRNAs, proteins, and phosphoproteins between tumors and MNUs. Related to Figure 3, Additional file 3: Fig. S3. A. Lists of mRNAs, proteins, and phosphoproteins used for principal component analysis (PCA). B. Lists of differentially expressed mRNAs, proteins and phosphoproteins (Wilcoxon rank-sum test, FDR < 0.01, Tumors/MNUs ratio > 2 or < 1/2) in tumors and MNUs. C. A list of genes with significant patient survival difference on both protein and mRNA level. D. A list of candidate target genes of UC.

**Additional file 16. Table S4**: Differentially expressed proteins between MIBCs and NMIBCs. Related to Figure 4, Additional file 4: Fig. S4. A. Lists of differentially expressed proteins in MIBCs and NMIBCs. B. Lists of differentially expressed proteins in chromosome 5p gain compared with WT. C. The list of 9 significant positive cis-effects on chromosome 5p.

**Additional file 17. Table S5**: Multi-omics characteristics of proteomic subtypes in 116 UC samples. Related to Figure 5, Additional file 5: Fig. S5. A. Information of immune clusters. B. Genes upregulated in each proteomic subtype. C. Lists of upregulated proteins in one of the three proteomic subtypes. D. Contributions of 30 COSMIC signatures in 113 UC patients. E. Genes of each Signatures used in this figure. F. Survival analysis of selected factors.

**Additional file 18. Table S6**: Differentially expressed among immune clusters. Related to Figure 6, Additional file 6: Fig. S6. A. Data analysis by xCell. B. Information of Immune cluster. C. Data analysis by estimate. D. Peaks correlated with immune score. E. Expression of immune checkpoint. F. Pathways upregulated in each immune cluster. G. Targets of NFKB1. H. Information of patients received PD-1 immunotherapy. I. TRAF2 identified in patients.

**Additional file 19. Table S7**: Differentially expressed among immune clusters. Related to Figure 7, Additional file 7: Fig. S7. A. Gene analysis by WGCNA. B. Pathways upregulated in modules. C. Genes were screened from the MEblue module. D. Genes upregulated in each histologic group. E. Pathways upregulated in each histologic group.

**Additional file 20. Table S8**: Modification identified in PGK1 and PKM2. Related to Figure 8. A. Modification identified in PGK1 and PKM2.

## Data Availability

The mass spectrometry proteomics and phosphoproteomic data were deposited in the ProteomeXchange Consortium (http://proteomecentralproteomexchange.org) via the iProX partner repository with the dataset identifier IPX0003522000. The transcriptomic data are accessible in NODE (https://www.biosino.org/node) under the accession number OEP002774. As publicly sharing of the raw genomic data is restricted by the regulation of the Human Genetic Resources Administration of China, detailed results of whole-exome sequencing were included in Additional file 13: Table S1. The raw sequencing data are available for non-commercial purposes under controlled access because of data privacy laws, and access can be obtained by request to the corresponding authors. The request will be passed within 1 week and then the users will be given a download link valid for 1 years to download the raw data.

## Abbreviations

FFPE: Formalin-fixed paraffin-embedded
FOT: Fraction of total
FDR: False discovery rate
CNA: Copy number alteration
FPKM: Fragments per kilobase of transcript per million mapped reads
HPLC: High-performance liquid chromatography
iBAQ: Intensity-based absolute quantification
IHC: Immunohistochemistry
KSEA: Kinase substrate enrichment analysis
MS: Mass spectrometry
OS: Overall survival
SMGs: Significantly mutated genes
ssGSEA: Single sample gene set enrichment analysis
TCGA: The Cancer Genome Atlas
TF: Transcription factor
TG: Target gene
WES: Whole-exome sequencing
XIC: Extracted-ion chromatogram
ACN: Acetonitrile
AJCC: American Joint Committee on Cancer
