# Supplementary figures and images for: Integrated proteogenomic characterization of urothelial carcinoma of the bladder
Source: J Hematol Oncol. 2022 Jun 3;15:76. doi: 10.1186/s13045-022-01291-7 (PMC9164575; doi:10.1186/s13045-022-01291-7)

# Supplementary Figure 1

A

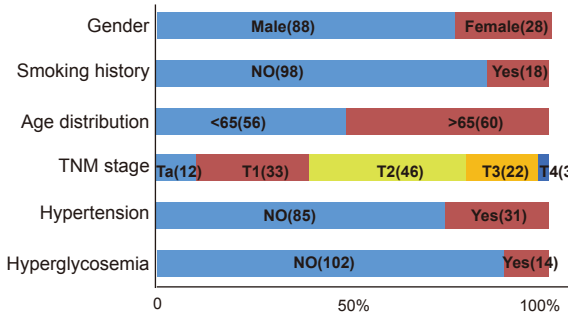

B

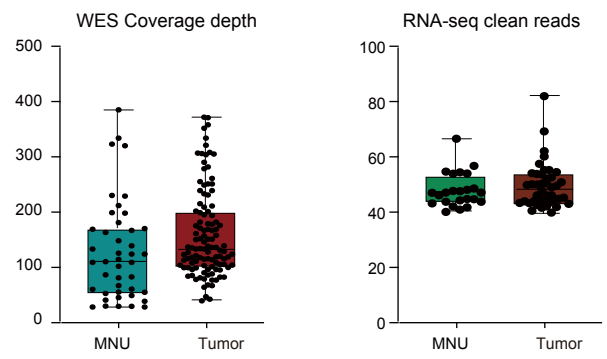

C

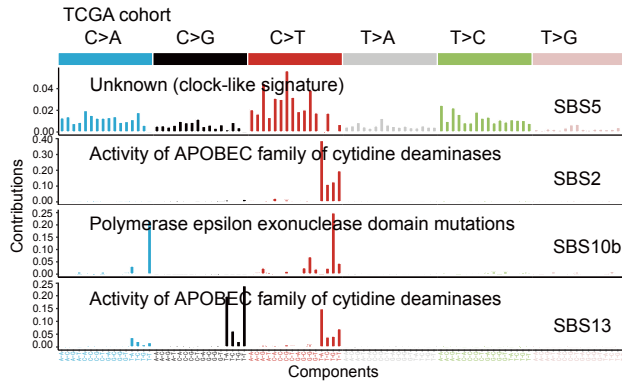

D

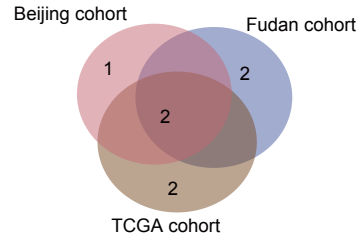

E

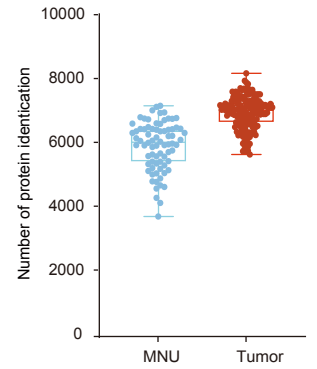

F

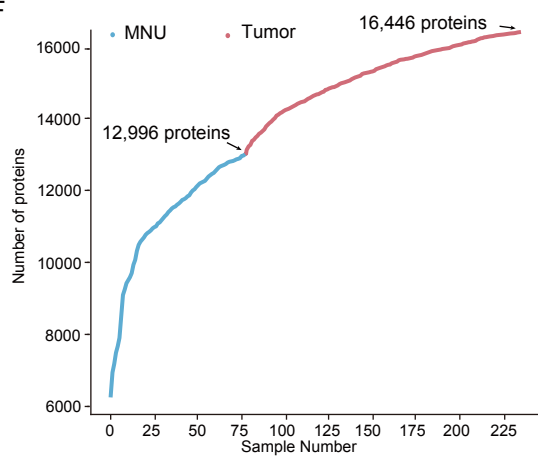

G

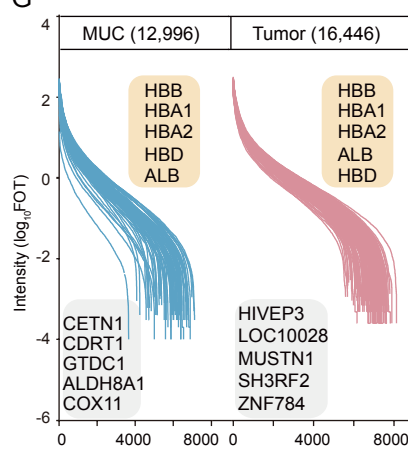

H

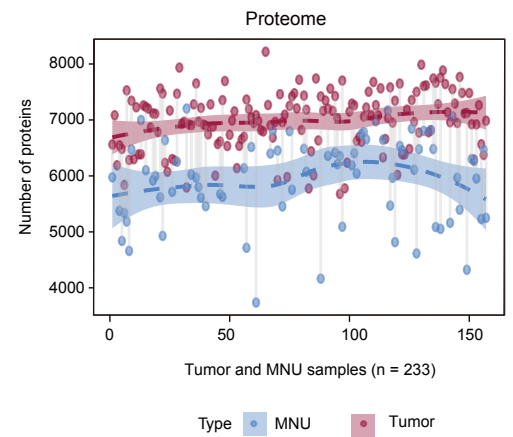

I

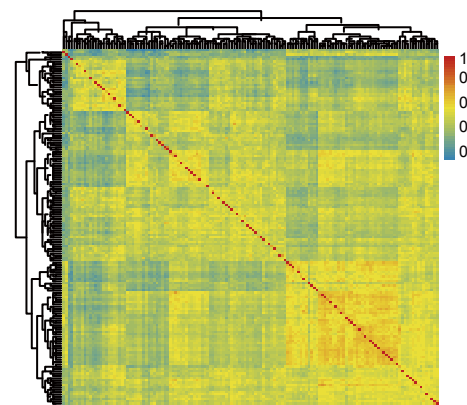

J

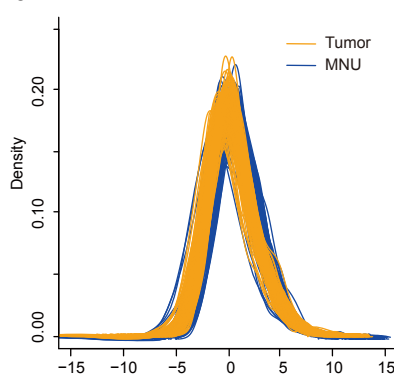

K

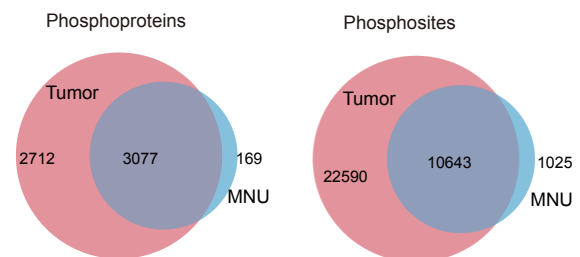

L

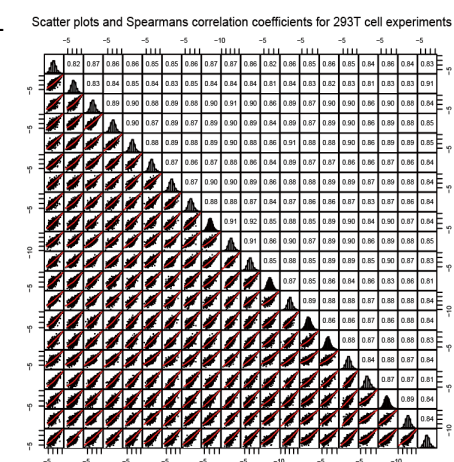

M

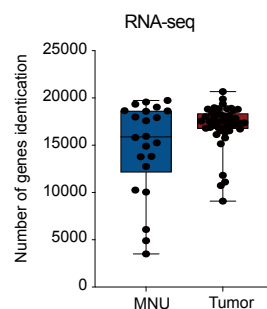

N

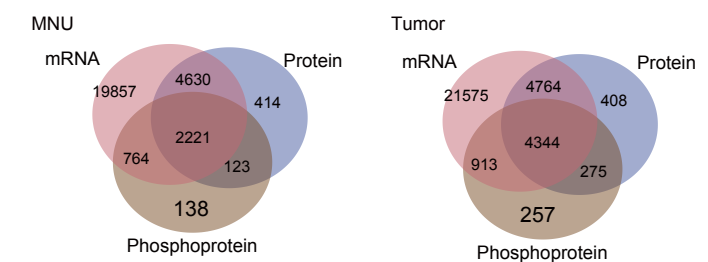

Supplement: Supplementary file 1 — Additional file 1. Fig. S1: Multi-omics landscape of UC samples. Related to Figure 1. (A) Clinical data of our cohort. (B) Left: Sequencing depths of WES for tumors tissues and MNU tissues. Right: All input reads in RNA-seq for tumors tissues and MNU tissues. (C) The four mutational signatures identified in TCGA cohort by Sigminer analysis. (D) VENN plot of signatures identified among different cohort. (E) Proteins identified in tumor tissues and MNU tissues. (F) Cumulative number of protein identifications. Blue presents MNU samples; red presents tumor tissues. (G) Dynamic ranges of identified protein abundances identified in tumor tissues and MNU tissues. (H) The numbers of proteins identified in MNU tissues (blue) and tumor tissues (red). (I) Correlated matrix of 233 sample proteomes (Spearman’s correlation coefficients). Red color indicates high correlation; blue color indicates low correlation. (J) Distribution of protein abundances in tumors and MNUs by density plot. A unimodal distribution (dip test) was observed. All samples passed proteomic quality control. (K) Number of phosphoproteins (left) and phosphosites identified in tumors and MNUs. (L) Quality control of mass spectrometry using tryptic digest of 293T cells. The top-left half of the panel represents the pairwise Spearman’s correlation coefficients of samples and the bottom-right half of the panel depicts the pairwise scatter plots from sample comparison. (M) Number of genes identified in tumors and MNUs using RNA-seq. (N) The overlap of genes identified in the proteome, phosphoproteome and transcriptome. Top: MNU tissue, bottom: tumor tissue. [file 13045_2022_1291_MOESM1_ESM.pdf]

Supplementary Figure 2

A

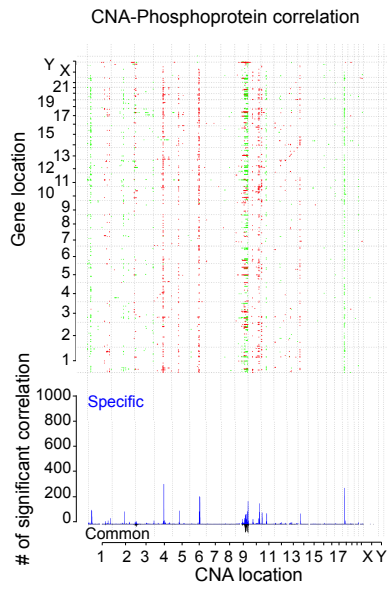

D

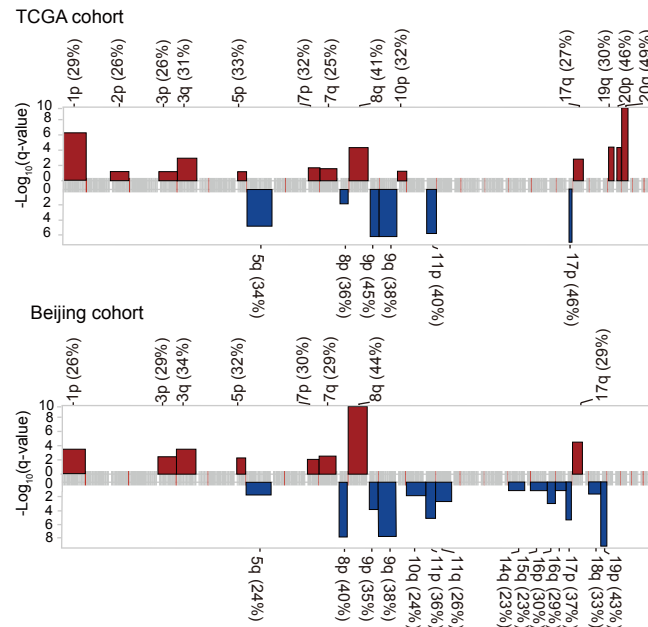

E

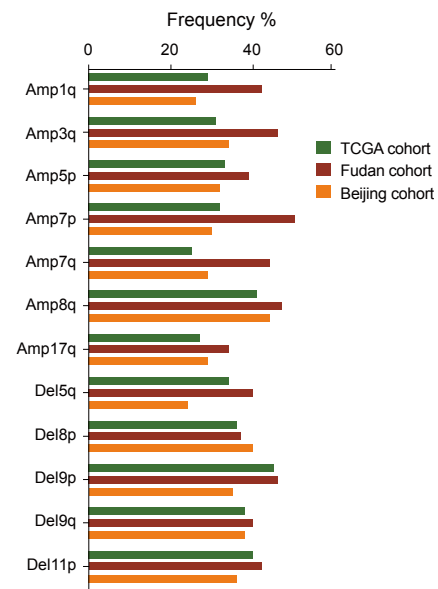

B

Specific significant *Cis* effects gene with pathway enrichment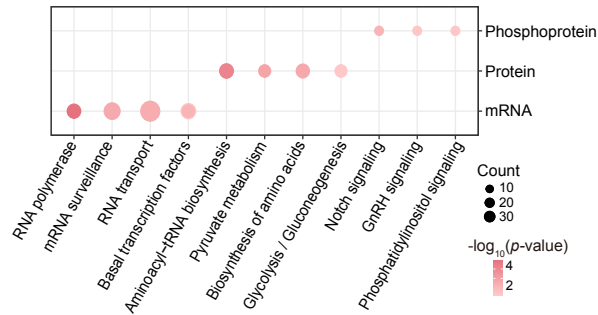

C

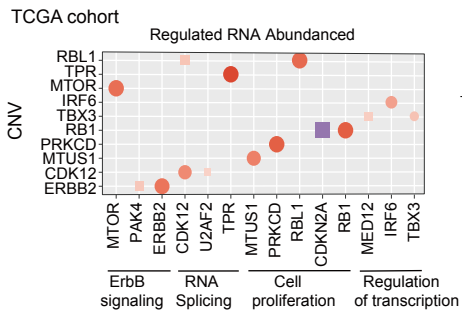

F

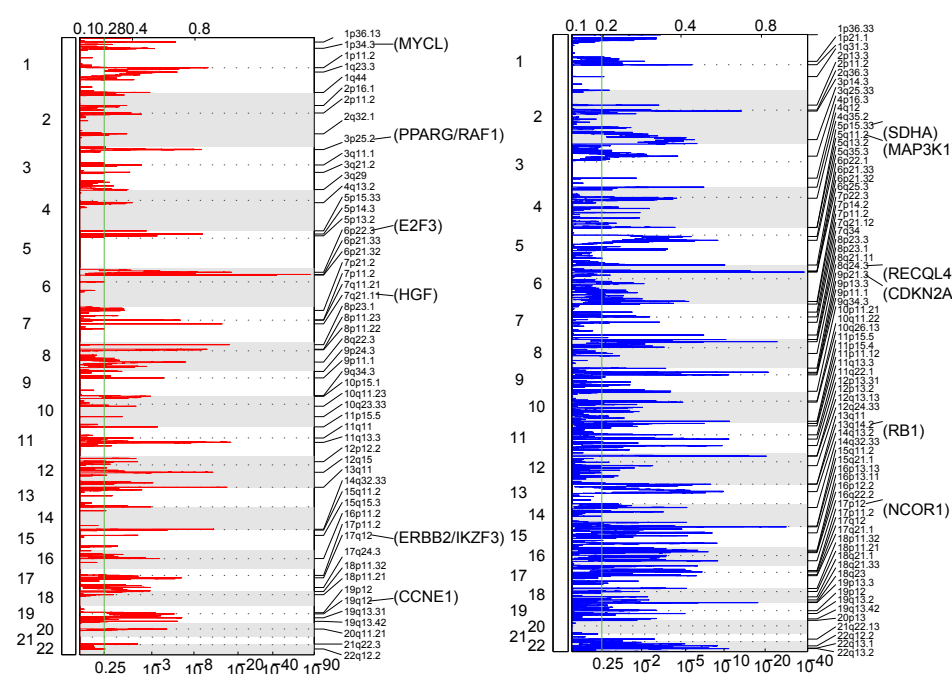

Supplement: Supplementary file 2 — Additional file 2. Fig. S2: Impact of copy number alteration on mRNA and protein expression. Related to Figure 2. (A) Functional impacts of CNA on phosphoprotein. Top panel: positive and negative correlations were indicated by red and blue colors, respectively. Bottom panel: number of phosphoproteins that were significantly associated with a specific CNA. (B) Pathways enriched for respective specific cis-effects. (C) 10 CAGs and pathways they affected in TCGA cohort. (D) Arm-level CNAs in TCGA cohort and Beijing cohort. Red denotes amplification and blue denotes deletion. (E) Compared arm-level CNAs among different cohorts. (F) Genome-wide focal amplifications and deletions. Chromosomal locations of peaks of significantly recurring focal amplifications (red) and deletions (blue) were filtered by FDRs. Peaks were annotated with candidate driver oncogenes or tumor suppressors. [file 13045_2022_1291_MOESM2_ESM.pdf]

# Supplementary Figure 3

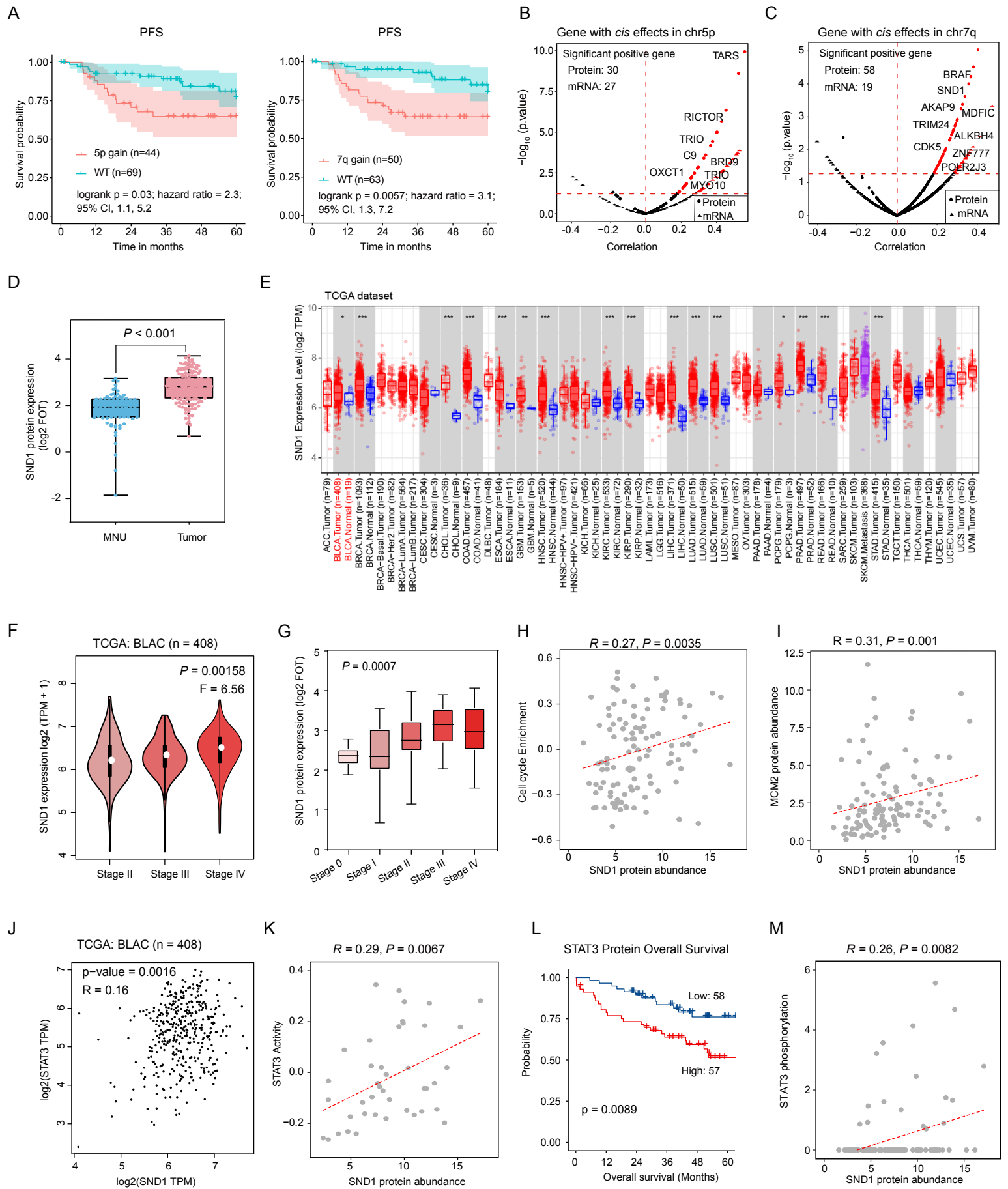

Supplement: Supplementary file 3 — Additional file 3. Fig. S3: Impact of copy number alteration on mRNA and protein expression. Related to Figure 2. (A) Left: Kaplan–Meier curves for progression-free survival based on the chromosome 5p gain and WT groups. Right: Kaplan–Meier curves for progression-free survival based on the chromosome 7q gain and WT groups. The p value was calculated by log-rank test. 95% confidence interval was also presented. (B–C) Volcano plot showing the cis-effect genes on chromosome 5p (B) and 7q (C). The dots represent proteins; the triangles represent mRNA. The p values were calculated by Spearman's correlation test. (D–E) The expression status of the SND1 gene in different cancer subtypes from the TCGA dataset using TIMER2 (@@@timer.cistrome.org/) (E) and protein abundance of SND1 in tumor and MNU groups (D). The p values were calculated by Wilcoxon rank-sum test. * P < .05; ** P < .01; *** P < .001. (F–G) Expression levels of the SND1 were analyzed by the main pathological stages of the BLCA TCGA cohort (F) and our cohort (G) Log2 (TPM + 1) was applied for log-scale (p value from Kruskal–Wallis test). (H) the correlation between enriched cell cycle pathway and SND1 protein abundance. (I–J) Correlation of SND1 protein abundance with MCM2 protein change (I) and the correlation between SND1 and STAT3 in TCGA BLCA cohort (J) (p value from Spearman's correlation test). (K) Correlation between SND1 protein abundance and estimated STAT3 activity (p value from Spearman's correlation test). (L) Overall survival analyses of UC patients with high or low levels of STAT3 protein abundance (p value from log-rank test). (M) Correlation of SND1 protein abundance with STAT3 phosphorylation change (phosphoprotein). [file 13045_2022_1291_MOESM3_ESM.pdf]

# Supplementary Figure 4

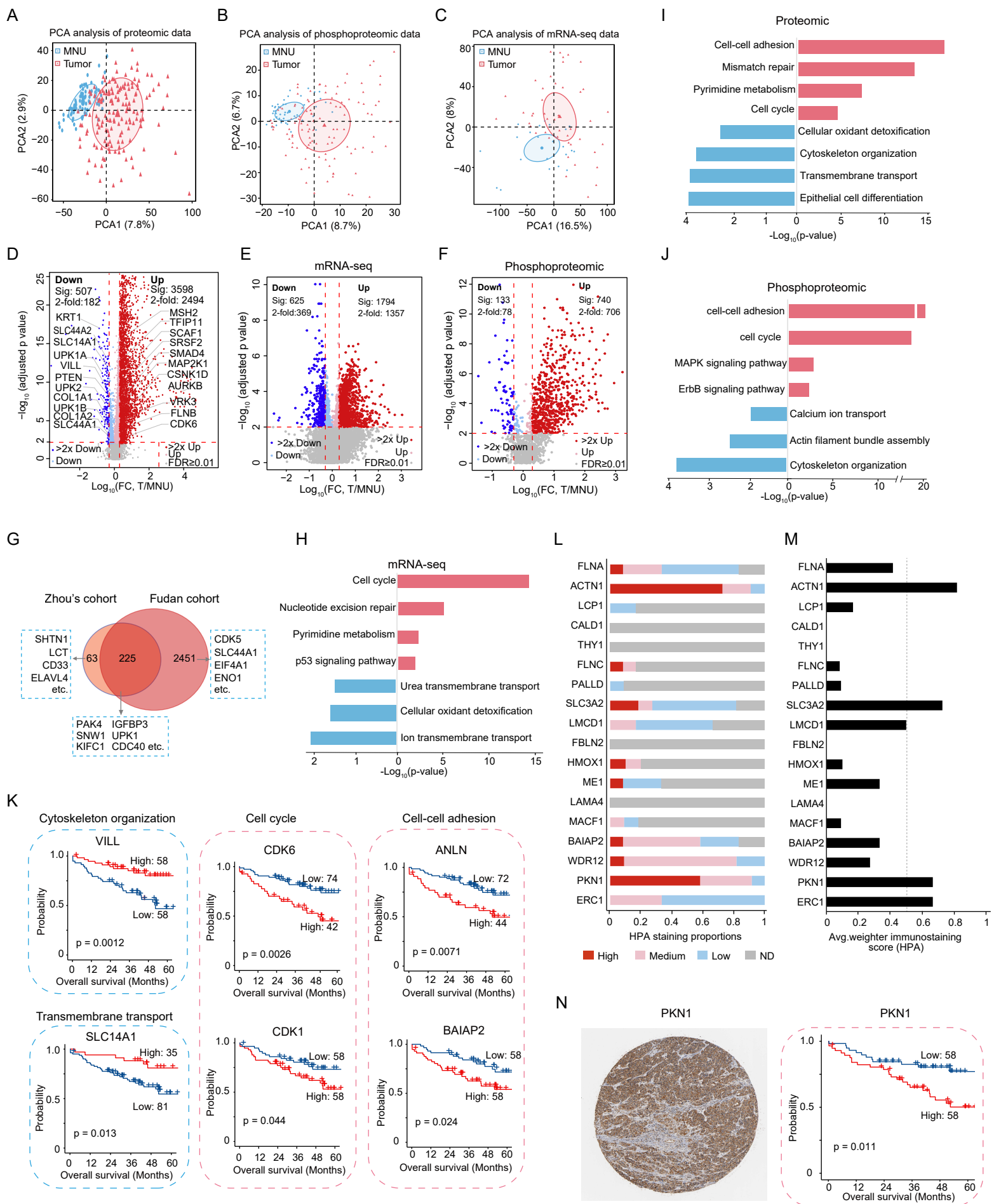

Supplement: Supplementary file 4 — Additional file 4. Fig. S4: Integrative analyses of transcriptomic, proteomic, and phosphoproteomic Data in UC Samples. Related to Figure 3. (A–C) Principal component analysis (PCA) of proteomic data (5,546 proteins) (A), phosphoproteomics (1,672 phosphoproteins) (B), and RNA-Seq (27,829 genes) (C) between tumors and MNUs. Red dots: tumors; blue dots: MNUs. (D–F) Proteins (D), genes (RNA-Seq) (E), and phosphoproteins (F) abundance differences between tumors or MNUs (p from Wilcoxon rank-sum test). (G) VENN plot of urothelial cancer-type-specific proteins identified between Zhou's cohort and Fudan cohort. (H–J) Pathways enriched for differentially expressed genes (H), proteins (I), and phosphoproteins (J) in tumors and MNUs. (K) Representative proteins from one of the four biological pathways and their association with prognosis (overall survival) (p value from log-rank test). (L–M) Proportions of urothelial bladder tumors with high, medium, or low staining, or not detected (ND) as reported by the Human Protein Atlas (HPA) (L) and tumor-cell specific immunohistochemistry (IHC) staining scores defined by the HPA (M). (N) IHC images for PKN1 proteins from HPA (left) and their association with clinical outcomes (overall survival) in all patients (p value from log-rank test). [file 13045_2022_1291_MOESM4_ESM.pdf]

# Supplementary Figure 5

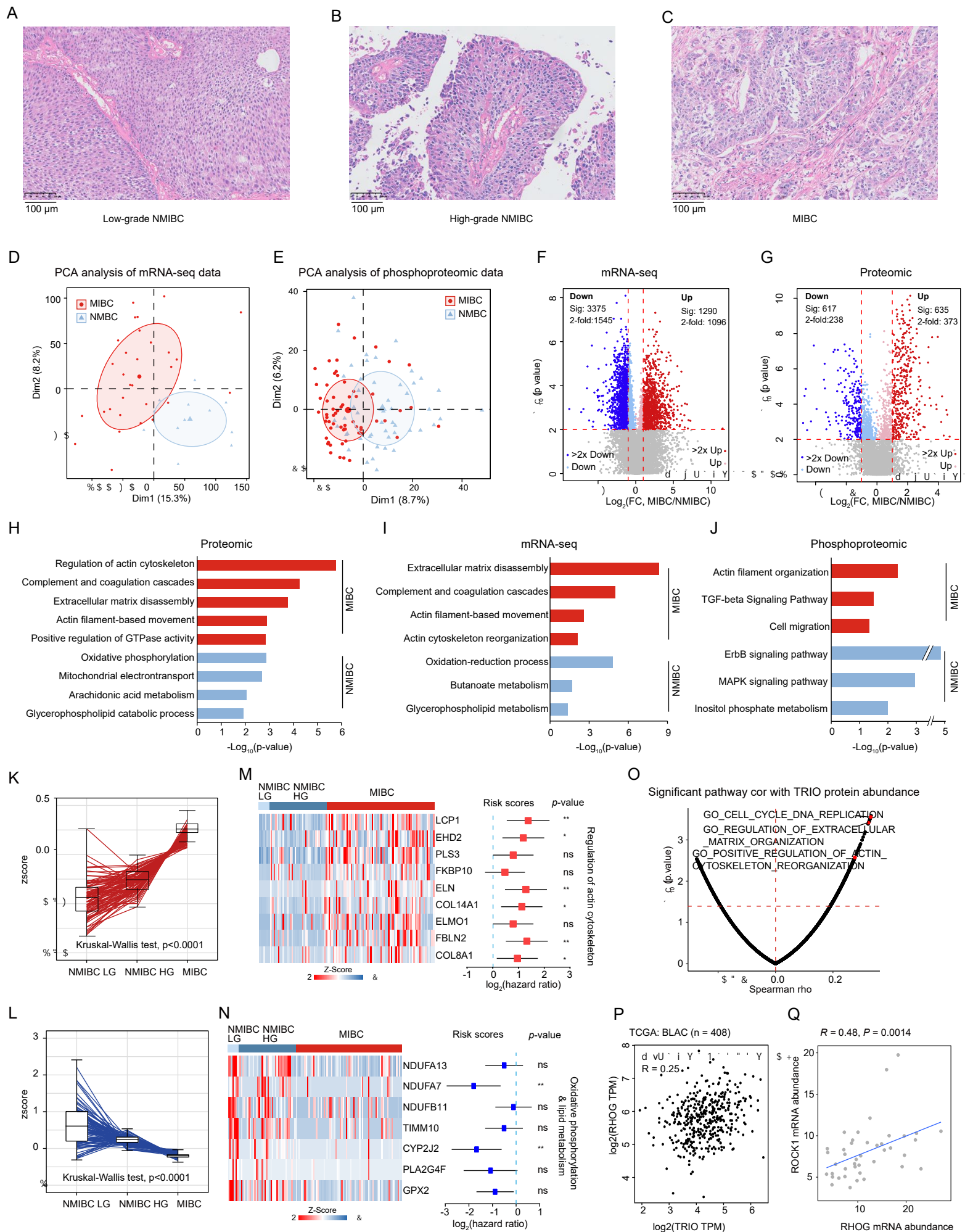

Supplement: Supplementary file 5 — Additional file 5. Fig. S5: Proteogenomic analysis between NMIBC and MIBC. Related to Figure 4. (A–C) H&E-stained slides of low-grade NMIBC (A), high-grade NIMBC (B), and MIBC tissues (C). (D–E) PCA of RNA-Seq (27,752 genes) (D) and phosphoproteomics (2,014 phosphoproteins) (E) between NMIBC and MIBC. (F–G) Genes (RNA-Seq) (F) and proteins (G) abundance differences between NMIBC and MIBC (Wilcoxon rank-sum test). (H–J) Pathways enriched for differentially expressed proteins (H) genes (RNA-Seq) (I) and phosphoproteins (J) in NMIBC and MIBC. (K–L) Line plots and boxplots of proteins involved in the crucial pathways from low-grade NMIBC, high-grade MIBC to MIBC. (M–N) Left, heatmap of protein abundance among low-grade NMIBC, high-grade MIBC, and MIBC. Middle, prognostic risk scores (overall survival) of each protein. The middle red points indicate log2-based hazard ratio for each protein; endpoints represent lower or upper 95% confidence intervals. Right, biological function category of the proteins. (O) Volcano plot showing the correlation between enriched Gene Ontology biological processes and TRIO protein abundance. (P) Correlation between TRIO and RHOG in the TCGA BLCA cohort. (Q) Correlation between RHOG mRNA abundance and ROCK1 mRNA abundance. The p values in (O)–(Q) were calculated by Spearman's correlation test. [file 13045_2022_1291_MOESM5_ESM.pdf]

Supplementary Figure 6

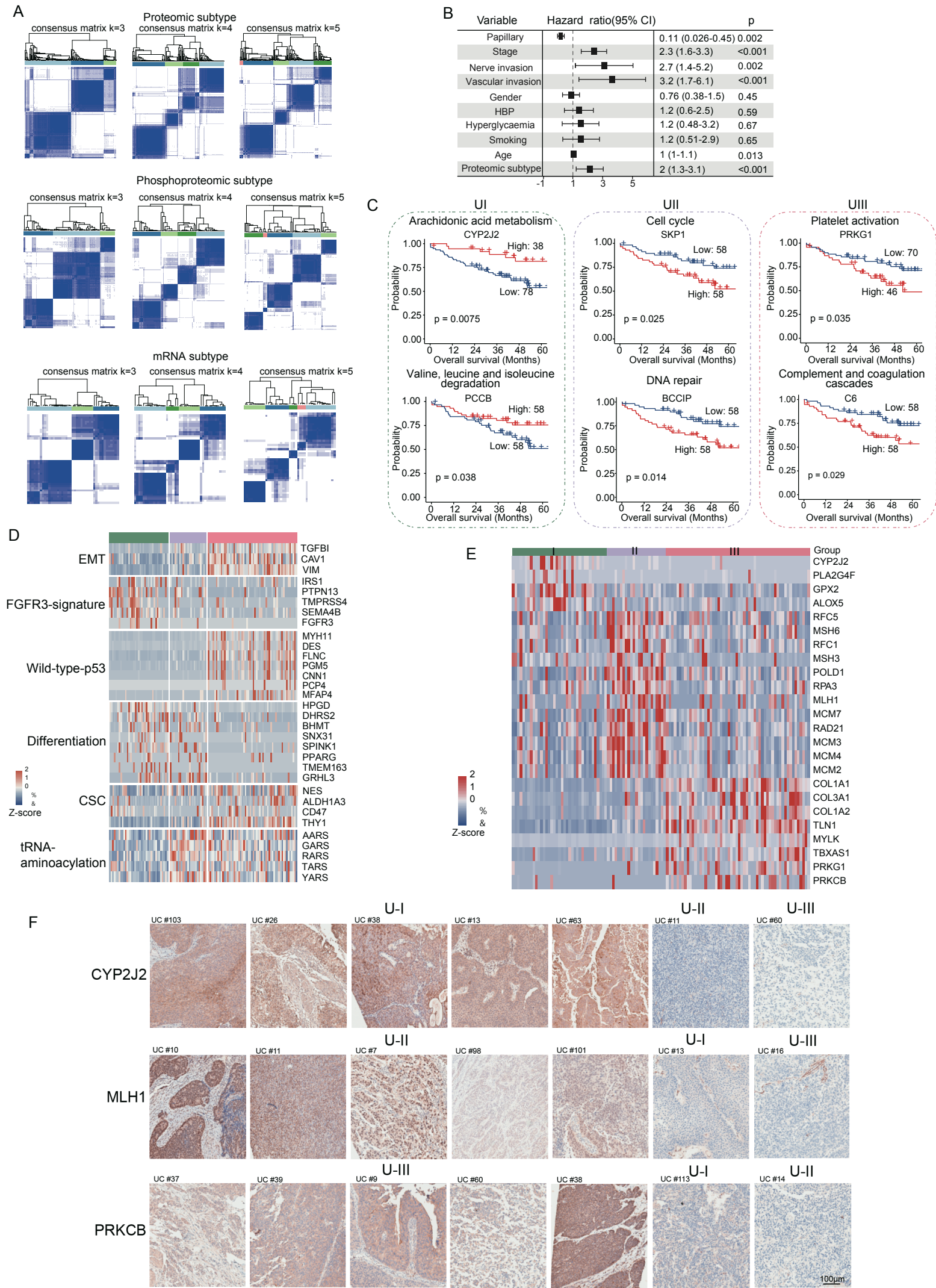

Supplement: Supplementary file 6 — Additional file 6. Fig. S6: Proteomic subtypes of UC and signature proteins. Related to Figure 5. (A) Consensus matrices of identified clusters (K=3), top: Clusters based on proteomic analysis data; median: clusters based on RNA data; bottom: clusters based on phosphoproteomic data. (B) Forest plot for multivariate Cox regressions for papillary, stage, never invasion, vascular invasion, gender HBP, hyperglycemia, smoking, age, and proteomic subtype, with the reference variable for each covariate set to the best-survival subtype. The main effects are presented as hazard ratios with 95% confidence intervals. (C) Signature proteins of each proteomic subtype and their association with prognosis (overall survival) (p value from log-rank test). (D) Gene expression signature scores. (Kruskal–Wallis test, p < 0.05). (E) Proteins that were differentially expressed in the three proteomic subtypes. (F) IHC profiling of proteomic subtype markers in UC. FFPE sections were stained for CYP2J2, MLH1 and PRKCB protein markers in UC tumor tissues. The scale bar indicates 100 μm. [file 13045_2022_1291_MOESM6_ESM.pdf]

# Supplementary Figure 7

A

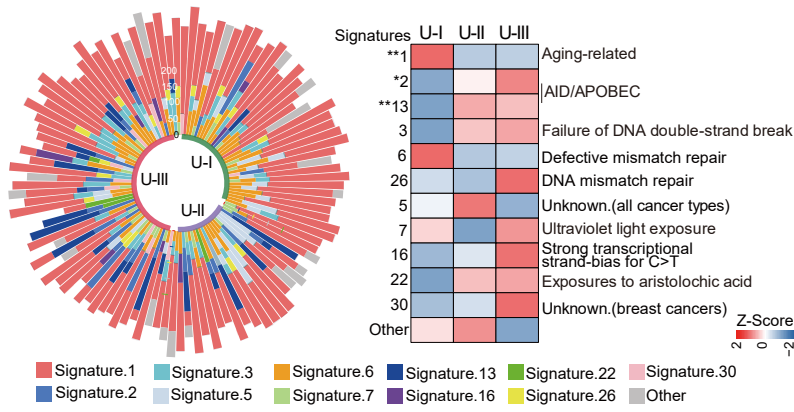

B

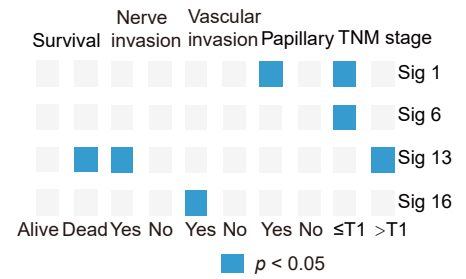

C

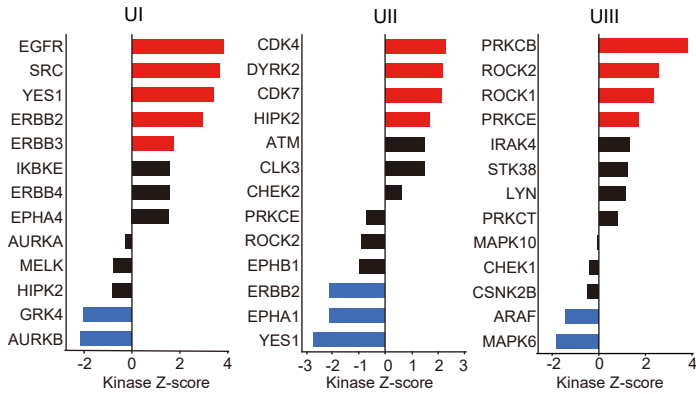

D

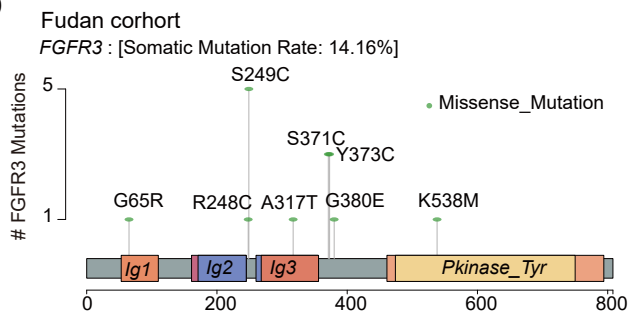

E

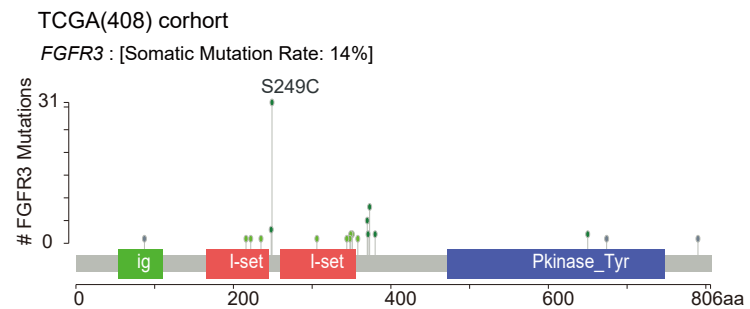

Supplement: Supplementary file 7 — Additional file 7. Fig. S7: Proteomic subtypes of UC and signature proteins. Related to Figure 5. (A) Distributions of main COSMIC signatures in the proteomic subtypes across 116 UC patients. Right: Heatmap of the comparison between different proteomic subtypes. (B) Association of mutational signatures with clinical features (Wilcoxon rank-sum test, p < 0.05). (C) Evaluation of kinase activities by KSEA in tumors across the three proteomic subtypes. (D) Mutational hotspots of FGFR3 in this study. (E) Mutational hotspots of FGFR3 in TCGA cohort. [file 13045_2022_1291_MOESM7_ESM.pdf]

Supplementary Figure 8

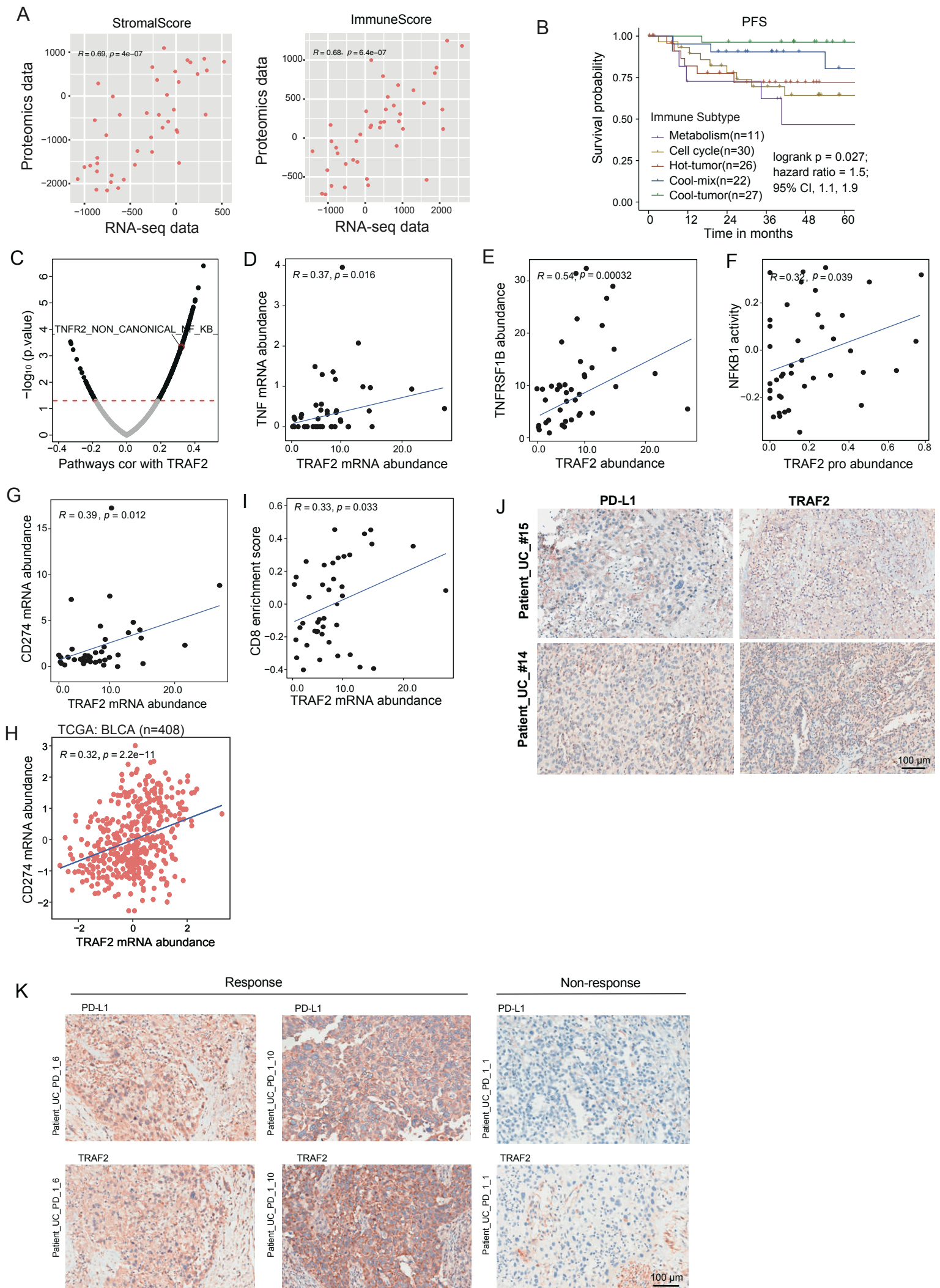

Supplement: Supplementary file 8 — Additional file 8. Fig. S8: Immune cell infiltration in UC tumors. Related to Figure 6. (A) Comparison between RNA-seq and global proteomics for estimating immunity (right) and stromal cell (left) infiltration based on 43 UC tumor samples. (B) Kaplan–Meier curves for progression-free survival of different immune clusters (p value from log-rank test). (C) Volcano plot showing the correlation between enrichment pathway and TRAF2 protein Abundance. (D) Scatter plot showing the correlation between the mRNA abundance change of TRAF2 and the mRNA abundance change of TNF. (E) Scatter plot showing the correlation between the mRNA abundance changes in TRAF2 and TNFRSF1B. (F) Correlation between average mRNA abundance and estimated NFKB1 activity change. (G) Scatter plot showing the correlation between the mRNA abundance changes in TRAF2 and CD274. (H) Correlation between TRAF2 mRNA abundance and CD274 protein change changes in the TCGA BLCA cohort. (I) Scatter plot showing the correlated between the mRNA abundance change of TRAF2 and CD8 enrichment score. (J) TRAF2 identified in patients with PD-L1-positive by IHC in Fudan cohort. The scale bar indicates 100 μm. (K) IHC of TRAF2 and PD-L1 in PD-L1-response and non-response. The scale bar indicates 100 µm. [file 13045_2022_1291_MOESM8_ESM.pdf]

Supplementary Figure 9

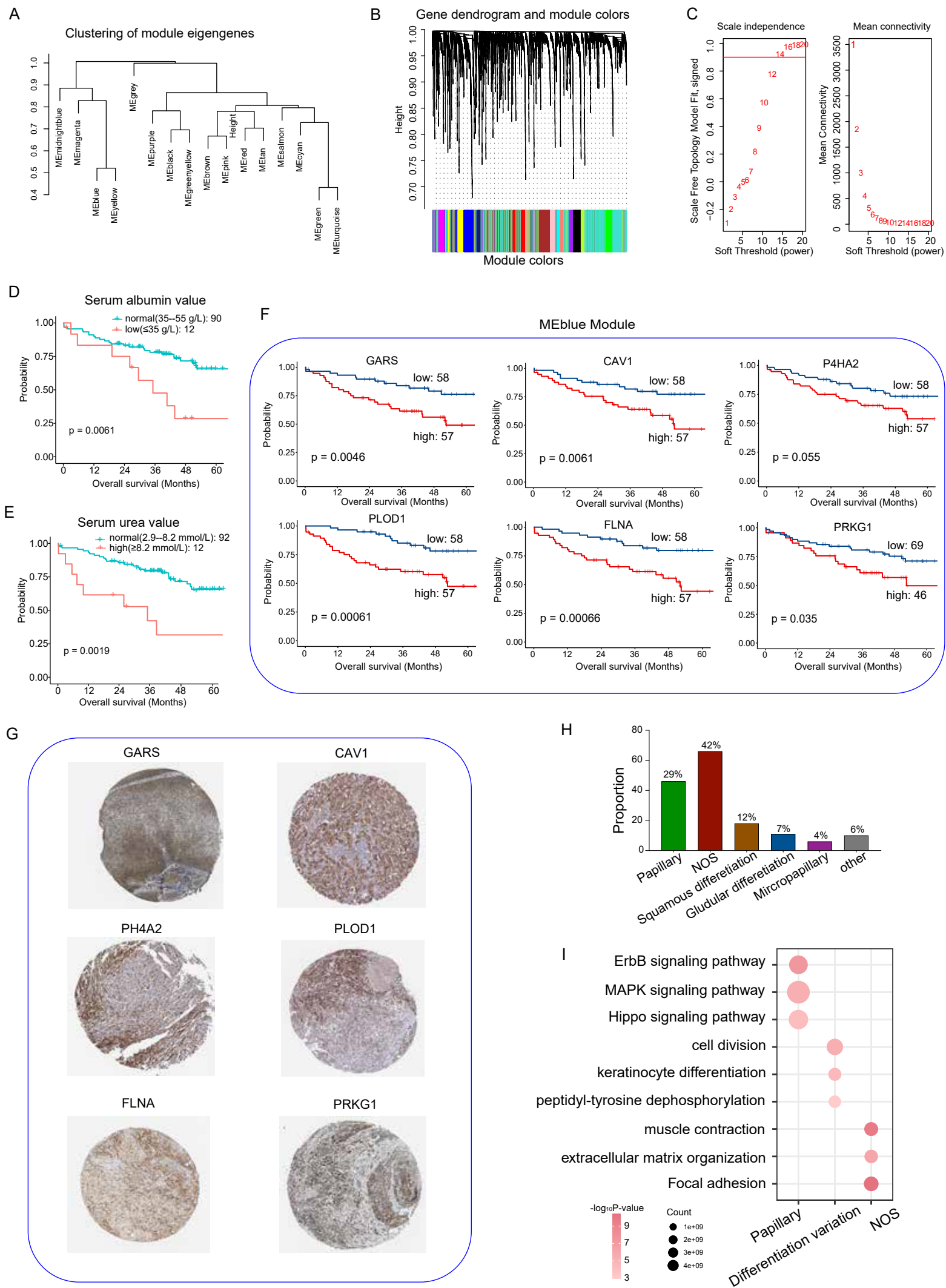

Supplement: Supplementary file 9 — Additional file 9. Fig. S9: Clinical outcomes associated with proteomic and phosphoproteomic profiles. Related to Figure 7. (A) Clustering dendrogram of samples based on their Euclidean distance. All samples are located in the clusters and also passed the cutoff thresholds. (B) Clustering dendrogram of genes, with dissimilarities based on topological overlap, together with assigned module colors. (C) left: the scale-free fit index (y-axis) as a function of the soft-thresholding power (x-axis); right: mean connectivity (degree, y-axis) as a function of soft-thresholding power (x-axis). (D–E) Clinical features (Serum albumin value and urea value) associated with prognosis (overall survival) (p value from log-rank test). (F) Representative proteins from the blue module and their association with prognosis (overall survival) (p value from log-rank test). (G) IHC images for GARS, CAV1, PH4A2, PLOD1, FLNA, and PRKG1 proteins in HPA database. (H) The distribution of different histological variations. (I) Pathways were enriched in different histological variations, based on phosphoproteome (Wilcoxon rank-sum p < 0.05). [file 13045_2022_1291_MOESM9_ESM.pdf]

Supplementary Figure 10

A

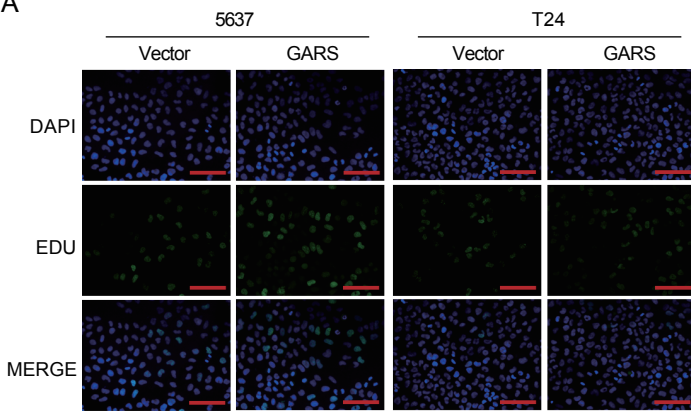

B

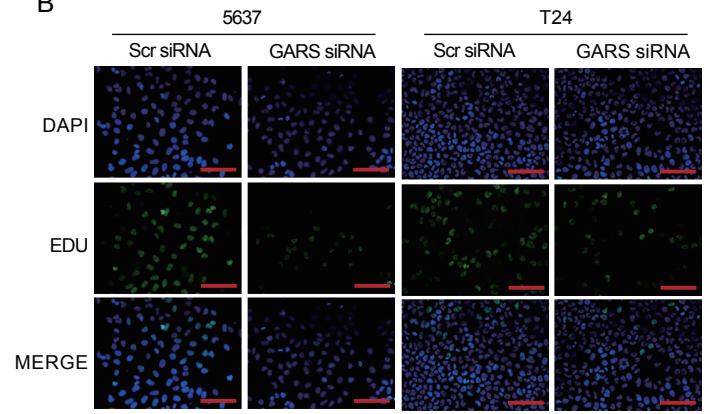

C

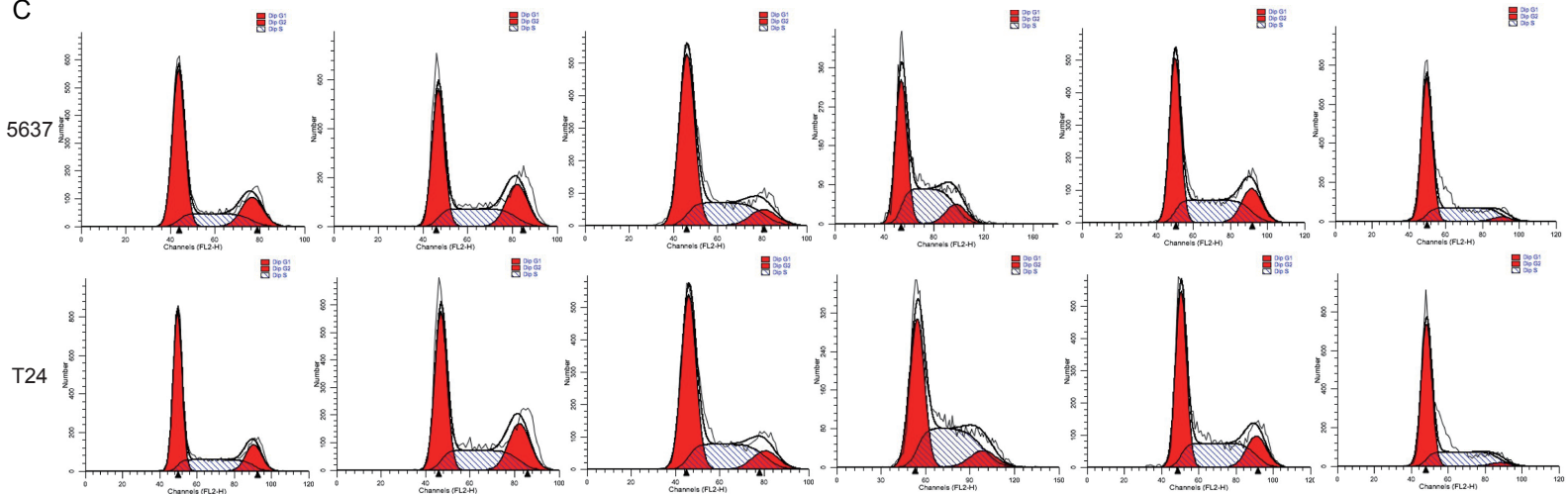

D

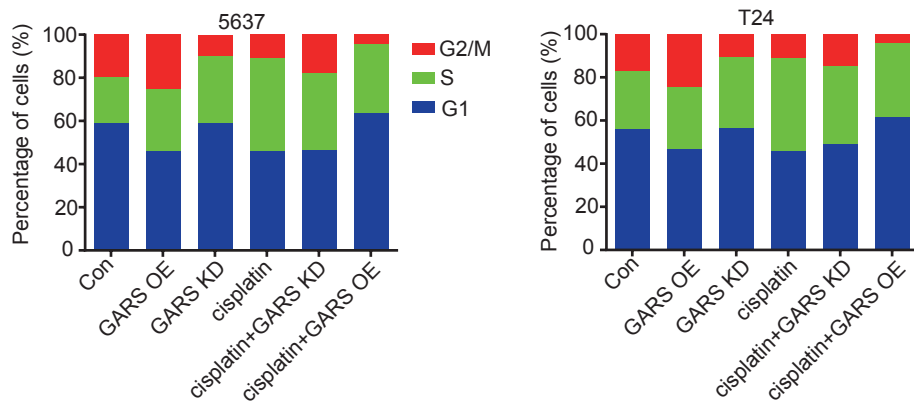

E

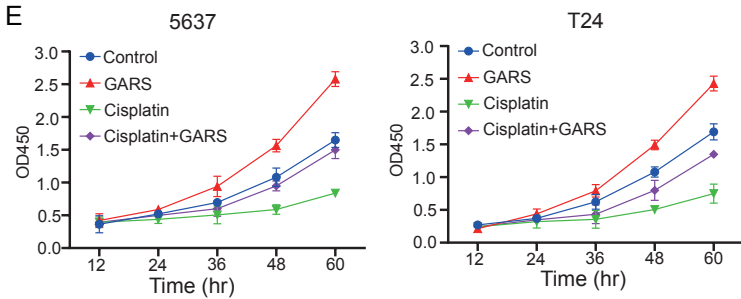

F

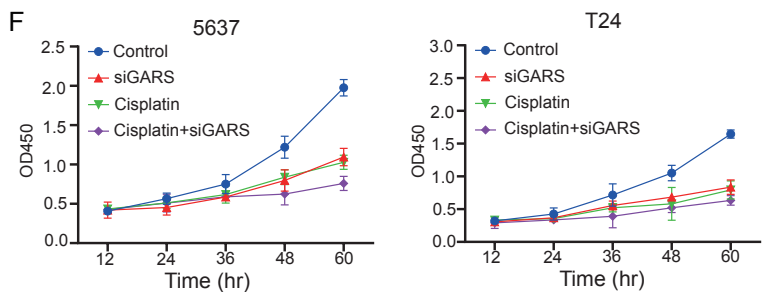

Supplement: Supplementary file 10 — Additional file 10. Fig. S10: GARS promotes bladder cancer cell proliferation through non-canonical function. Related to Figure 8. (A)–(B) DNA synthesis. Scale bar=100 µm. (C)–(D) Cell cycle progression. (E)–(F) in T24 and 5637 cell lines subjected to GARS-overexpressing or knocking down. Scale bar=100 µm. [file 13045_2022_1291_MOESM10_ESM.pdf]

Supplementary Figure 11

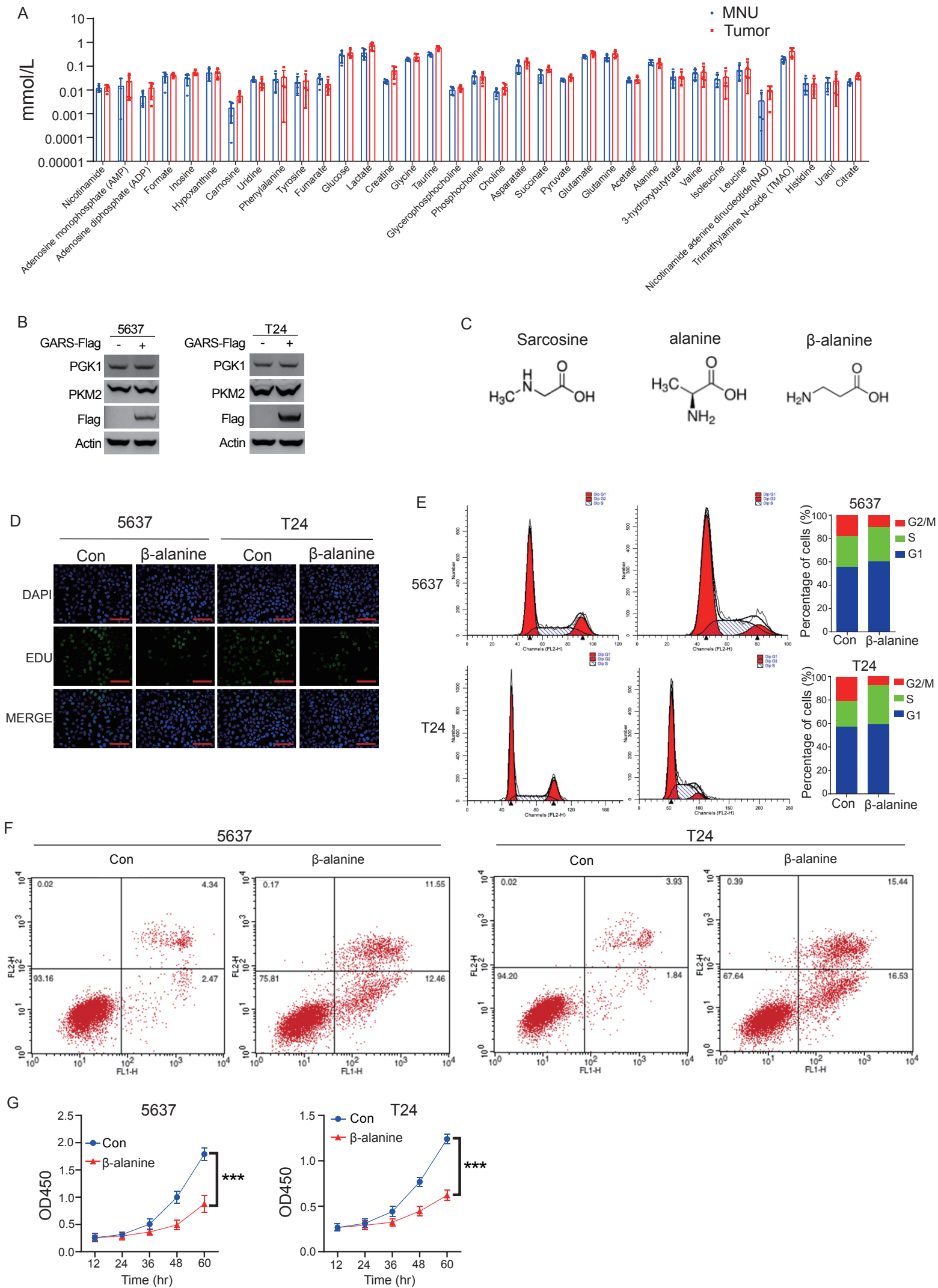

Supplement: Supplementary file 11 — Additional file 11. Fig. S11: GARS promotes bladder cancer cell proliferation through non-canonical function. Related to Figure 8. (A) Metabolite levels of tumor and normal tissues of bladder cancer, as detected by NMR. (B) Levels of PGK1 and PKM2 in both 5637 and T24 GARS-overexpressing cells. (C) The structural analogs of glycine. (D–G) The effects of beta-alanine on DNA synthesis (D), cell cycle progression (E), cell apoptosis (F), and slowed down the cell proliferation (G), in both 5637 and T24 cells; Scale bar=100 µm. [file 13045_2022_1291_MOESM11_ESM.pdf]

Supplementary Figure 12

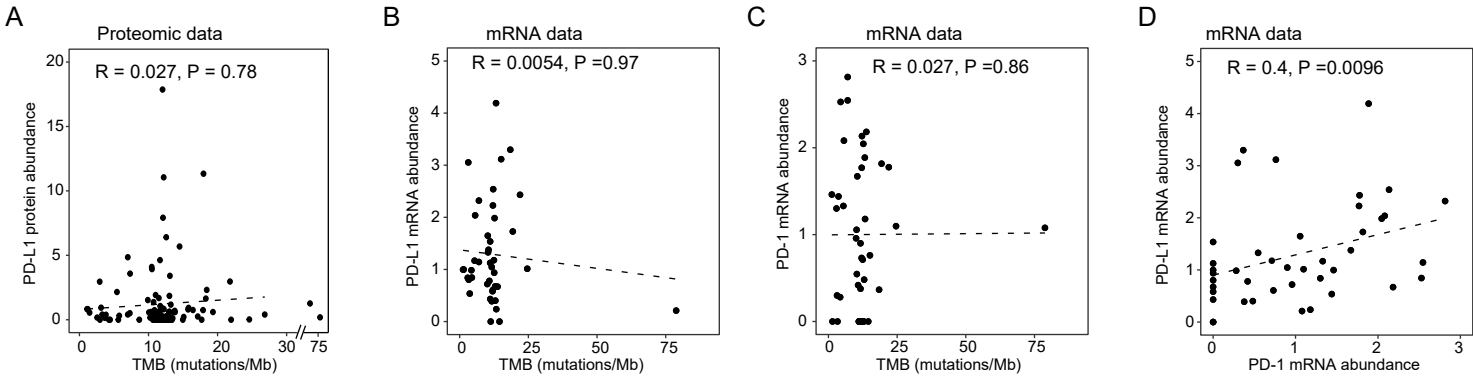

Supplement: Supplementary file 12 — Additional file 12. Fig. S12: Relationships among PD-L1 expression, PD-1 expression, and TMB. (A) Correlation analysis between PD-L1 expression and TMB in proteomic data. (B) Correlation analysis between PD-L1 expression and TMB in mRNA data. (C) Correlation analysis between PD-1 expression and TMB in mRNA data. (D) Correlation analysis between the expression of PD-L1 and PD-1 in mRNA data. The p values (A)–(D) were calculated by Spearman's correlation test. [file 13045_2022_1291_MOESM12_ESM.pdf]
